# Distribution of global sea turtle nesting explained from regional-scale coastal characteristics

**DOI:** 10.1038/s41598-023-50239-5

**Published:** 2024-01-08

**Authors:** Jakob C. Christiaanse, José A. A. Antolínez, Arjen P. Luijendijk, Panagiotis Athanasiou, Carlos M. Duarte, Stefan Aarninkhof

**Affiliations:** 1 Department of Hydraulic Engineering, Delft University of Technology, Delft, Netherlands; 2 Deltares , Delft, Netherlands; 3 Biological Sciences and Engineering Division, King Abdullah University of Science and Technology (KAUST), Thuwal, Saudi Arabia

**Keywords:** Environmental sciences, Ocean sciences, Civil engineering, Ecology

## Abstract

Climate change and human activity threaten sea turtle nesting beaches through increased flooding and erosion. Understanding the environmental characteristics that enable nesting can aid to preserve and expand these habitats. While numerous local studies exist, a comprehensive global analysis of environmental influences on the distribution of sea turtle nesting habitats remains largely unexplored. Here, we relate the distribution of global sea turtle nesting to 22 coastal indicators, spanning hydrodynamic, atmospheric, geophysical, habitat, and human processes. Using state-of-the-art global datasets and a novel 50-km-resolution hexagonal coastline grid (Coastgons), we employ machine learning to identify spatially homogeneous patterns in the indicators and correlate these to the occurrence of nesting grounds. Our findings suggest sea surface temperature, tidal range, extreme surges, and proximity to coral and seagrass habitats significantly influence global nesting distribution. Low tidal ranges and low extreme surges appear to be particularly favorable for individual species, likely due to reduced nest flooding. Other indicators, previously reported as influential (e.g., precipitation and wind speed), were not as important in our global-scale analysis. Finally, we identify new, potentially suitable nesting regions for each species. On average, $23\%$ of global coastal regions between $-39^\circ$ and $48^\circ$ latitude could be suitable for nesting, while only $7\%$ is currently used by turtles, showing that the realized niche is significantly smaller than the fundamental niche, and that there is potential for sea turtles to expand their nesting habitat. Our results help identify suitable nesting conditions, quantify potential hazards to global nesting habitats, and lay a foundation for nature-based solutions to preserve and potentially expand these habitats.

## Introduction

Climate change and human activity pose many different challenges to sea turtles, including the flooding and erosion of their nesting habitats—sandy beaches^1,2^. Although sea turtles have successfully evolved and adapted to habitat changes over millions of years, their slow population growth rates mean they are unable to recover quickly from population declines (recovery rates of sea turtle populations can range from several decades to 100 years^3^). This makes them particularly vulnerable to relatively fast-paced changes to their nesting habitat^4,5^, such as current human and climate-induced effects on nesting beaches (e.g., rising temperatures and sea levels)^6^.

An imminent threat is the flooding and erosion of nesting beaches during events with high water levels and/or waves (e.g., storms and tropical cyclones). Incubating nests can get inundated or even washed away, significantly decreasing hatching success^4,7^. Furthermore, storm erosion can significantly alter beach morphology, which may affect nesting over several seasons^8^. On longer time-scales, structural erosion and coastal squeeze may gradually diminish the amount of nesting habitat available to sea turtles (e.g.,^6,9,10^). These threats are expected to intensify in the future, because many nesting beaches lie in (1) the tropics^11^, the most vulnerable zone to increased future coastal flooding due to sea level rise^12^; (2) regions prone to tropical cyclone activity^4,13^; and (3) developing countries, where coastal areas are expected to become increasingly developed in the future^14^. Nature-based solutions—for example, through turtle-friendly design of sand nourishments or by adding coastal vegetation or reefs to provide coastal protection from flooding and erosion—may offer promising opportunities to preserve and even expand nesting habitats. However, we first need to understand the environmental characteristics that enable sea turtle nesting to design such solutions.

Many studies have attempted to identify preferential nesting conditions for sea turtles, but the results are often inconclusive or inconsistent among studies (e.g.,^15,16^). Generally, incubating nests require certain temperature and humidity windows^17^, and nesting females seemingly try to limit the exposure of their nests to conditions outside these windows. Hence, temperature^15,17–20^, humidity^15,17–20^, geomorphology (e.g., beach elevation and slope)^15,21–26^, hydrodynamics (e.g., waves and water levels)^16,27,28^, and human activity^25^ have all been mentioned as potentially influential factors on nesting suitability. Yet, to date there are hardly any robust ranges for the multivariate characteristics believed to enable turtle nesting. They vary significantly between nesting beaches of different species, and even populations of the same species^18,23^. The fact that all sea turtles exhibit some degree of nest site fidelity, returning to nest in the region where they hatched^29^, further complicates causation. It therefore remains uncertain how turtles select their nesting beaches^30^.

Most past studies have focused on individual (and usually popular) nesting beaches, rarely comparing them to ’non-nesting’ beaches, which may make it difficult to discern suitable conditions^30^. Moreover, focusing on individual beaches limits the analysis to a small subset of the species’ realized niche and hinders the ability to distinguish patterns on a regional level. Only few studies have tried to identify suitable characteristics for nesting on a scale that exceeds individual beaches within the same region^15,18,27,31^. Pike^18^ was the only one of these who analyzed and predicted nesting suitability over large parts of the global coastline, but his analysis was limited to variables related to temperature and precipitation, and did not include any information on hydrodynamics, geomorphology, habitat, or human activity near nesting sites.

Here, we relate sea turtle nesting activity to a broad range of environmental characteristics of global coastal regions. All five globally distributed species are considered: the loggerhead turtle (*Caretta caretta*, CC), green turtle (*Chelonia mydas*, CM), hawksbill turtle (*Eretmochelys imbricata*, EI), leatherback turtle (*Dermochelys coriacea*, DC), and olive ridley turtle (*Lepidochelys olivacea*, LO). We assess the influence of 22 coastal indicators related to hydrodynamic, atmospheric, geophysical, habitat, and human processes on the global distribution of sea turtle nesting grounds, using state-of-the-art global datasets combined with tailored machine learning techniques. We then identify spatially homogeneous patterns in the most influential indicators through a global-scale cluster analysis of coastal regions. Using the clusters, we further investigate the relationship between influential coastal indicators and nesting activity and identify new, potentially suitable nesting regions (fundamental niches) for all five species. The results (1) help identify and quantify suitable nesting conditions and main hazards in each (potential) nesting region; and (2) guide research on the design of nature-based solutions to restore and preserve nesting habitats from present and future coastal impacts, and to enable the colonization of potentially suitable nesting regions^32^.

## Methods

**Figure 1:**
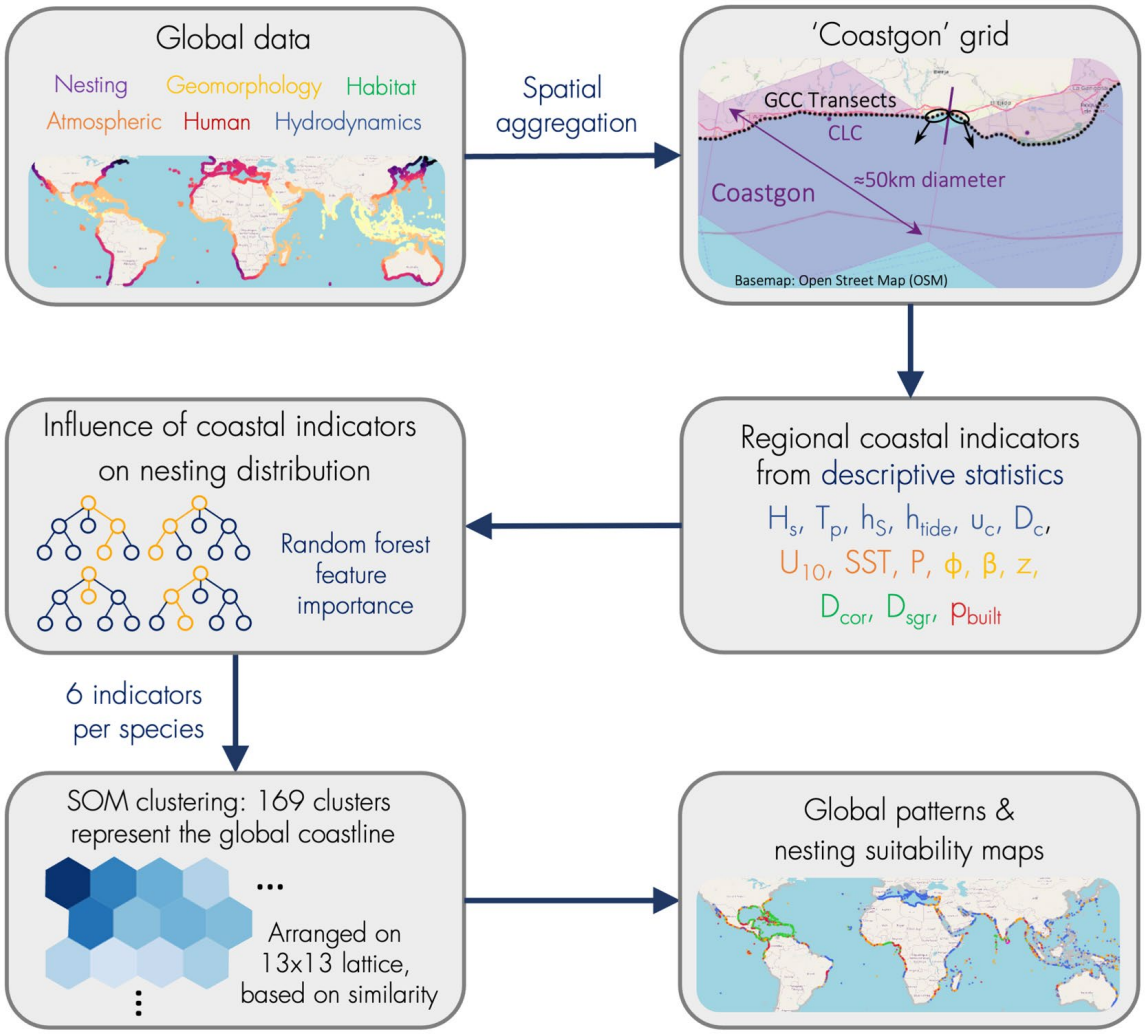
Flowchart showing the methodology of this study. First, data gathered from eight global datasets was aggregated onto the Coastgon grid. Next, we derived 22 coastal indicators for each Coastgon from these data using descriptive statistics. We then assessed the influence of each indicator on the distribution of global sea turtle nesting by fitting a random forest model. Based on the feature importance we selected a subset of six influential indicators per turtle species. Then we identified patterns in the six indicators among global sea turtle nesting regions, by clustering the Coastgons based on the six indicators selected for each species, through self-organizing maps (SOM). Finally, we identified new, potentially suitable nesting regions for each species, based on the SOM clusters.

We characterized the World’s coastline using state-of-the-art global datasets on hydrodynamic, atmospheric, geophysical, habitat, and human variables (Table 1). These data were spatially aggregated onto a novel 50-km-resolution hexagonal coastline grid, called *Coastgons*^33^, to perform our analysis on a single resolution (a Coastgon being a hexagonal coastal cell). We derived 22 indicators describing temporal and spatial variability of coastal characteristics for each Coastgon (Table 2). Next, we assessed the importance of these indicators on the global distribution of sea turtle nesting grounds through a machine learning regression technique (random forests), and selected a subset of six influential indicators for each sea turtle species. We identified patterns in the coastal characteristics of global sea turtle nesting habitats by clustering the six indicators of each species with self-organizing maps, a machine learning clustering technique. Finally, we identified new, potentially suitable nesting regions for each species, based on the clusters, and illustrated these in nesting suitability maps. The methodology is explained in more detail in the following sections and illustrated in Figure 1.

### Global data

We used global data describing sea turtle nesting activity (SWOT^34,35^ and WIDECAST^36^), waves, wind, sea surface temperature, and precipitation (ERA5^37^), tide and surge levels (GTSM^38^), ocean currents (ORAS5^39^), geomorphology and coastal land use (GCC^40^), and the distribution of coral reefs (Allan Coral Atlas^41^) and sea grass meadows (UNEP-WCMC^42^). Each dataset is briefly described below and summarized in Table 1.

- **SWOT**, the State of the World’s Sea Turtles project, provides a database of global sea turtle nesting sites since 2004 (although including earlier data), compiled from data contributions of over 550 individuals and organizations around the World and is hosted by the OBIS-SEAMAP platform^34,35^. As such, the spatial resolution and accuracy vary significantly, ranging from different sites on the same beach, to groups of beaches (e.g., a small island with multiple beaches). Each site is labeled either as ’Quantified’, ’Unquantified’, or ’No-Nesting’. Since our Coastgon resolution is large enough to aggregate the different spatial resolutions of the nesting sites and we did not weight Coastgons based on the number of nesting sites, we used all quantified and unquantified nesting sites ($N = 5383$ unique sites, many with multiple species, see Supplementary Fig. S3).
- **WIDECAST**, the Wider Caribbean Sea Turtle Conservation Network, provides a nesting atlas spanning 45 Caribbean countries and territories, compiled from data provided by more than 200 contributors^36^. There is significant overlap between the SWOT and WIDECAST datasets, but WIDECAST provides additional data for several regions where SWOT is incomplete (e.g., Brazil). As we aggregate all nesting sites to the Coastgon grid, the overlap is not a problem for the analysis, hence we used all available nesting sites ($N = 1336$ unique sites, many with multiple species, see Supplementary Fig. S3).
- **ERA5**, the ECMWF Reanalysis v5, is a gobal atmospheric reanalysis, providing hourly time series of atmospheric, land, and oceanic climate variables from 1940 to present^37^. The model is split into two coupled structured global grids, ERA5-wave at a resolution of $0.5^\circ$ for wave variables (e.g., sea and swell wave heights and periods) and ERA5-atmos at a resolution of $0.25^\circ$ for atmospheric, land, and oceanic climate variables (e.g., wind, land moisture, and sea surface temperature). We used time series of significant wave height (wind sea and swell combined), peak wave period, 10 m wind speed, sea surface temperature, and total precipitation from 1980 to 2021 (42 years).
- **GTSMv3.0**, the Global Tide and Surge Model, solves tidal propagation and was forced with wind and sea level pressure fields from ERA5 to provide a 10-minute interval time series of global tide and storm surge levels^38^. The spatial resolution of the output nodes varies around the globe, but is generally around $20-50$ km near the coast, with higher resolution along European coastlines ($<10$ km). We used tide and surge data computed for 1985 to 2014 (30 years).
- **ORAS5**, the Global Ocean ReAnalysis System, is a global, eddy-permitting ocean and sea ice ensemble reanalysis, based on five members^39^. It provides global monthly mean values of ocean data from 1958 to present, on a structured grid with $0.25 ^\circ$ horizontal resolution and at 75 depth levels up to 5500 m deep. We used time series of ocean current velocities at 0.5 m below the surface, from 1980 to 2021.
- **GCC**, Global Coastal Characteristics, is a dataset of hydrodynamic, geophysical, and socioeconomic indicators along the global coastline^40^. Data is provided at shore-normal transects which follow the coastline at 1-km intervals. We used data on geomorphology (slopes and topography), shoreline orientation, and built environment in the coastal zone.
- The **Allan Coral Atlas** provides a global coral reef extent map, based on reef occurrence probabilities computed through a convolutional neural network^41^. We used geo-referenced polygons of coral reefs covering all ocean basins.
- **UNEP-WCMC** provides a global distribution of seagrass meadows^42^, which is regularly updated since 2003, to reflect present conditions. We used geo-referenced polygons of seagrass meadows covering all ocean basins.

While SWOT and WIDECAST provide invaluable sources of nesting data, we are aware that these datasets are not complete and that some developing countries in particular are underrepresented in the database (see also Discussion). We therefore added some additional nesting sites to our analysis—specifically in regions where we know SWOT is incomplete—based on information provided by Shimada et al. (Red Sea)^43^, Shanker and Chowdhury (India and Pakistan)^44^, and Laloë and Hays (global)^45^. An overview of all nesting sites used for this study is presented in Supplementary Fig. S3.

Moreover, the variability in accuracy and completeness of the SWOT nesting data and the use of other global datasets were one of the reasons that we chose the Coastgon approach (see next section) with a resolution (50km) suited to regional analysis of coastal characteristics. A single nesting site in SWOT will lead to a corresponding ‘nesting Coastgon’, regardless of how many turtles nest there, or how many other nesting sites there are close by. This mitigates the issue of underreported and missing nesting sites.

**Table 1:** Summary of the global datasets used for this study.

Dataset	Data	Refs.	Type of data	Spatial dimensions	Temporal dimensions
SWOT	Nesting grounds	^34,35^	Observations	Geolocations, variable	n/a
WIDECAST	Nesting grounds	^36^	Observations	Geolocations, variable	n/a
ERA5-wave	Hydrodynamics	^37^	Reanalysis	Structured grid, $0.5 ^\circ$	1980–2021, hourly
ERA5-atmos	Meteorology	^37^	Reanalysis	Structured grid, $0.25 ^\circ$	1980–2021, hourly
GTSM	Tide and surge	^38^	Hindcast	Unstructured grid, variable	1980–2018, 10 min
ORAS5	Ocean currents	^39^	Reanalysis	Structured grid, $0.25 ^\circ$	1980–2021, monthly
GCC	Geomorphology	^40^	Mixed	Transects, 1 km	variable, single times
Allan Coral Atlas	Coral reef extent	^41^	Modelled	Geopolygons, variable	2020, single time
UNEP-WCMC	Seagrass locations	^42^	Observations	Geopolygons, variable	Since 2003

### Coastgons

We created a novel $\approx 50$-km-resolution hexagonal coastline grid, called *Coastgons*, dividing the Earth’s coastline between $-\,39^\circ$ and $48^\circ$ latitude into distinct coastal regions^33^. The latitude limits were chosen by adding a buffer to the latitudes of the most northern and southern known sea turtle nesting sites in the SWOT database^34^. We opted for a hexagonal cell grid over a point-based transect system as it better represents the contiguous nature of coastal regions. Additionally, the geometric properties of hexagons, such as uniform distance between neighboring cell centers and equitable partitioning of space, make them efficient and suitable for geospatial analyses and visualisation.

We used the H3 hexagonal hierarchical geospatial indexing system^46^ as a basis for the Coastgons. H3 covers the Earth’s surface with a hexagonal cell grid at 16 hierarchically leveled resolutions. The H3 grid was created by covering the 20 planar faces of an icosahedron with hexagonal cells and then projecting each face onto Earth’s surface using a gnomonic projection^46^. Each hexagon is then defined by the latitude/longitude coordinates (WGS84) of its vertices.

We created the Coastgons by overlaying the H3 grid (resolution 4) over the centroids of the coastline transects used in the GCC dataset^40^. The geospatial overlay selected every H3 hexagon that covered at least one GCC transect centroid, leading to many Coastgons that represented very short sections of coastline (*O*(10*km*)). To mitigate this issue, we refined the grid by filtering Coastgons based on their number of transects and neighboring Coastgons, while ensuring that the resulting coastline grid would not be interrupted by gaps. The GCC transects that fell into eliminated Coastgons, were subsequently matched to the nearest remaining Coastgon, up to a maximum distance of 100 km. Hence, in the final grid ($N = 5848$ Coastgons), every coastline transect within 100 km of the grid is represented by one Coastgon. Finally, we assigned a representative *coastline centroid* (CLC) to each Coastgon, given by the centroid of all transects linked to it.

The challenges of projecting a global grid over the Earth’s surface mean that not all H3 hexagons are regular (equilateral and equiangular), and they can vary in size (although the size is not correlated with the latitude due to the gnomonic projection used in H3)^46^. For the Coastgon grid, this results in a mean cell area of 1775 $\textrm{km}^2$ (standard deviation 242 $\textrm{km}^2$) and a mean diameter (distance between opposing vertices) of 52 km (standard deviation 3.7 km). We accepted this property, given our analysis did not involve any indicators that are directly linked to the the Coastgon size. The chosen resolution is similar to that of the coarsest global dataset used (ERA5-wave at $0.5^\circ \approx 55$ km at the equator). Hence, small-scale coastal features, like sheltered or embayed beaches, might not be resolved but regionally aggregated. We deemed this an acceptable trade-off, given our aim to identify regional patterns of spatio-temporal characteristics of coastal systems.

### Regional coastal indicators

We assumed that each Coastgon represents a spatially homogeneous coastal region, with a binary state regarding nesting activity: if it covered any known nesting sites, it was considered as a nesting region for the corresponding species. Each Coastgon’s coastal characteristics were represented by a set of 22 indicators derived from the global data, divided into five categories: hydrodynamic, atmospheric, geophysical, habitat, and human (Table 2). The number of data points from which each Coastgon’s indicators were computed depends on the dataset. Each GCC transect was linked to one Coastgon during the creation of the grid, so we used all transects linked to a Coastgon to compute its geophysical and human indicators. For gridded datasets (ERA5, GTSM, and ORAS5), the *k* nearest nodes to each CLC were used, up to a maximum of 100 km distance, where *k* depended on the resolution of the dataset ($k=1$ for ERA5-wave, $k=2$ for ERA5-atmos and ORAS5, and $k=3$ for GTSM). Distances were computed with the Haversine formula (shortest distance between two points on the surface of a sphere). If no node was within 100 km of a Coastgon’s CLC, a ’Not a Number’ (NaN) was assigned to that Coastgon.

The hydrodynamic and atmospheric indicators were computed from historical time series (42 years from 1980–2021 for ERA5 and ORAS5; 30 years from 1985–2014 for GTSM), while the geophysical, habitat and human indicators represent current or recent conditions (Table 1). When time series from multiple nodes were used for one Coastgon, we first computed the indicators separately from each time series, before averaging over the nodes to yield one value per indicator and Coastgon. Most indicators were derived through descriptive statistics, like the median (50th percentile) as a measure of center and the 95th percentile as a measure of extremes. For the peak wave period ($T_p$) we also included the standard deviation, as $T_p$ is often characterized by a bimodal distribution of swell and wind waves (a larger standard deviation indicating a bimodal wave climate). We included the standard deviation of the shoreline angle as a measure of shoreline complexity—a large standard deviation indicating many different shoreline orientations, hence a more complex coastline, (e.g., islands and embayed beaches). For indicators representing distances ($D_{c,03}$, $D_{cor}$, and $D_{sgr}$), calculations were based on the CLC of each Coastgon. A detailed explanation of how each indicator was derived from the global datasets is provided in the Supplementary Material, Section S1. The final dataset of 5848 Coastgons, characterized by 22 coastal indicators, is available through the 4TU.ResearchData repository^33^.

**Table 2:** Overview of the 22 regional coastal indicators for each coastgon, derived from the global datasets in Table 1.

Category	Variable	Indicator	Symbol	Unit	Dataset
Hydrodynamic	Significant waveheight	Median	$H_{s,med}$	m	ERA5 Ocean^37^
		$95^{th}$ percentile	$H_{s,p95}$	m	
	Peak wave period	Median	$T_{p,med}$	s	
		$95^{th}$ percentile	$T_{p,p95}$	s	
		Standard deviation	$T_{p,std}$	s	
	Surge level	Median	$h_{S,med}$	m	GTSM^38^
		$95^{th}$ percentile	$h_{S,p95}$	m	
	Tidal range	Mean	$h_{tide}$	m	
	Ocean current velocity	Median	$u_{c,med}$	m/s	ORAS5^39^
	Ocean current proximity	Distance to nearest current $\ge 0.3 m/s$	$D_{c,03}$	km	
Atmoshperic	10m wind speed	Median	$U_{10,med}$	m/s	ERA5 Atmos^37^
		$95^{th}$ percentile	$U_{10,p95}$	m/s	
	Sea surface temperature	Median	$SST_{med}$	$^{\circ }{C}$	
	Total precipitation	Median	$P_{med}$	mm/y	
Geophysical	Shoreline angle	Standard deviation	$\phi _{std}$	$^\circ$	GCC^40^
	Nearshore slope	Median	$\beta _{ns,med}$	–	
	Backshore slope	Median	$\beta _{bs,med}$	–	
	Max coastal elevation within 1km of shoreline	Median	$z_{max,med}$	m + msl	
		Standard deviation	$z_{max,std}$	m + msl	
Habitat	Coral reef proximity	Distance to nearest coral reef habitat	$D_{cor}$	km	Allan Coral Atlas^41^
	Seagrass meadow proximity	Distance to nearest seagrass habitat	$D_{sgr}$	km	UNEP-WCMC^42^
Human	Built env. in coastal zone	Mean % built environment	$p_{built}$	%	GCC^40^

They are divided into five categories: hydrodynamic, atmospheric, geophysical, habitat, and human.

### Influence of coastal indicators on sea turtle nesting distribution

To assess the influence of the 22 coastal indicators on the distribution of global sea turtle nesting, we employed random forests (RF)^47^. RF is a machine learning regression technique that constructs an ensemble of uncorrelated decision trees which predict a sample’s class, and returns the majority prediction of all trees. We selected RF because it is capable of capturing complex, non-linear relationships in the data. Furthermore, the RF model computes the contribution of each variable to the predictive accuracy of the decision trees and converts these to a relative feature importance. We also tested linear discriminant analysis and logistic regression models, but these could not achieve adequate model performance, hence we only used RF.

We fitted one RF model for each species, distinguishing between nesting (1) and non-nesting (0) Coastgons. We assessed the model performance by letting the trained model predict the category of each Coastgon (nesting vs. non-nesting) and computing three performance scores: (1) precision, which quantifies the proportion of correct ’nesting’ predictions out of all ‘nesting’ predictions; (2) recall, which quantifies the proportion of nesting Coastgons that is predicted correctly by the model; and (3) the F1 score, which is the harmonic mean of precision and recall, serving as a balanced measure of model performance. Based on the RF feature importance and our informed judgment, we then selected a subset of six influential indicators for each species for further examination in the cluster analysis.

Although RF is commonly used for predictive regression, here we used it as a dimensionality reduction technique. The aim was to identify patterns in the existing data, not to create the best generalized model to predict new, unlabeled data. Therefore, we decided to train and test the final RF models on the entire dataset. To test the robustness of our RF model we carried out a four-fold cross validation: we split the data into four equally sized partitions and trained four RF ‘sub-models’, each on a unique combination of three partitions (75% of the data). We then compared the RF feature importance of the four sub-models with the one trained on the full dataset. The feature importance remained consistent between the five models within each species (see Supplementary Fig. S2).

### Global patterns and nesting suitability maps

To identify patterns in the coastal characteristics of global sea turtle nesting habitats, we performed a cluster analysis on the six indicators selected for each species. First, the data was normalized using a custom percentile scaler, which scales each indicator to the range [0, 1[, such that 0 represents the minimum and 1 represents the $99.9\textrm{th}$ percentile (i.e., scaled values above the $99.9\textrm{th}$ percentile were larger than 1). We applied this custom scaler instead of more conventional methods, like *MinMax* or *standard* scaling, because it is more robust to outliers and doesn’t assume normally distributed data.

Next, we clustered the Coastgons based on the selected indicators, using *self-organizing maps* (SOM). SOM is a type of unsupervised neural network that groups high-dimensional data into *k* clusters and automatically projects these onto a two-dimensional lattice, preserving the topological properties of the data as much as possible^48^. Each cluster of Coastgons is represented by one neuron, which is a point in the six-dimensional parameter space. The algorithm starts with *k* predefined initial neurons, and iteratively adjusts these during the learning process to yield *k* distinct clusters. An intuitive, metaphorical description of the method is that one throws a fishing net over the data, and then moves each node (neuron) of the net to cover the data as best as possible.

The number of clusters, *k*, is predefined by the user and is often determined iteratively by evaluating SOMs for different values of *k*. We used SOMs with $k=169$ clusters, arranged on a 13 $\times$ 13 lattice. This number was determined iteratively through visual inspection of SOMs for different *k*, and using intra- and inter-cluster variance metrics (e.g., quantization error, silhouette score, and boxplots of each cluster). As initial neurons, we selected a subset of 169 Coastgons through a maximum dissimilarity algorithm. This algorithm ensures that the initial neurons are as dissimilar from each other as possible, meaning they span the parameter space more uniformly (i.e., the fishing net is stretched to the limits of the parameter space)^49^. We then computed the final 169 neurons using the MiniSom python library^50^.

To explain the distribution of global sea turtle nesting from the selected coastal indicators, we created a separate SOM for each species (i.e., for each subset of coastal indicators). Each cluster represents a group of coastgons with similar indicators. We then computed Spearman’s rank correlation coefficient, $\rho$, between the cluster medians of each indicator and the percentage of nesting Coastgons in each cluster. A positive (negative) correlation coefficient therefore implies that regions with larger (lower) values for a given indicator contain a higher percentage of nesting Coastgons.

We mapped representative sea turtle nesting regions around the globe for each of the five species, by classifying the Coastgons into three categories: ‘observed nesting’ (O), ‘potentially suitable’ (S), and ‘unsuitable’ (U). The latter two categories encompass all Coastgons without observed nesting. These Coastgons were labeled ‘potentially suitable’ if they were part of a cluster containing at least 10% observed nesting Coastgons, and otherwise ‘unsuitable’. To assess whether the distributions of the six indicators differed significantly across the three Coastgon categories, we applied the two-sample Kolmogorov-Smirnoff (KS) test, a non-parametric test suitable for non-normal data, to each pair-wise combination of categories. We adopted the common significance level of $\alpha = 0.05$ and adjusted the three p-values for each indicator using the Benjamini-Hochberg method, to limit inflated Type I errors due to multiple comparisons on the same indicator.

## Results

### Indicators influencing the global distribution of sea turtle nesting

**Figure 2:**
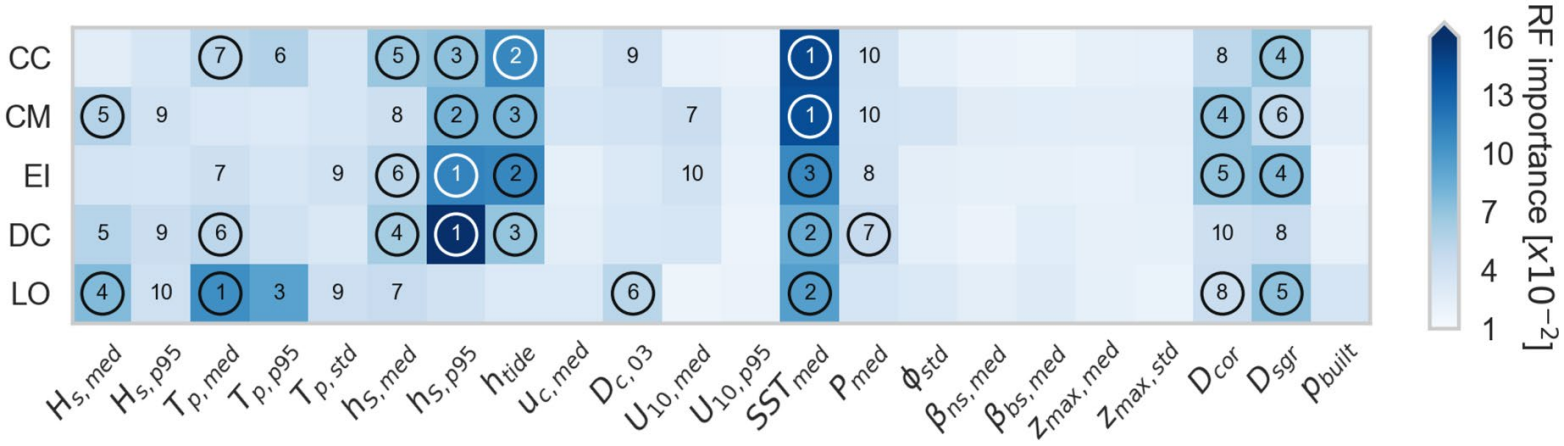
Matrix plot showing the RF feature importance of the 22 coastal indicators (Table 2), in separating nesting from non-nesting Coastgons. Each square represents one species/indicator pair and the blue scale indicates the magnitude of the feature importance. The numbers in the squares show the ranks of the ten most important indicators and the circles highlight the six selected indicators for each species, which are further examined in the cluster analysis. Figure created with Python 3.10.8.

We ranked the 22 coastal indicators based on their RF feature importance (see numbers of the top 10 ranks in Fig. 2). Sea surface temperature was the only indicator with consistently high rankings across all species. Other important indicators varied more among the species but mainly consisted of hydrodynamics and distance to the nearest coral/seagrass habitats. Notably, the geophysical indicators had low importance across all species. For loggerheads (CC), green turtles (CM), hawksbills (EI), and leatherbacks (DC), extreme surge ($h_{S,p95}$) and tidal range ($h_{tide}$) were important (top three ranks). For olive ridleys (LO), on the other hand, the wave climate (particularly the wave period) was more important than the water levels. Distance to the nearest seagrass habitat ranked in the top six for CC, CM, EI, and LO. Distance to the nearest coral reef was mainly important for CM and EI.

We selected a subset of six indicators for each species based on the RF feature importance and our informed judgment (circled indicators in Fig. 2). The subset does not strictly correspond to the top six ranks, as lower ranks sometimes had similar importance values (e.g., ranks 5–8 for DC). Additionally, a known characteristic of RF models is that feature importance may be spread over correlated variables, which doesn’t necessarily mean that both variables together are important, but an underlying process is. For example, the median ($T_{p,med}$) and extreme wave period ($T_{p,p95}$) have the highest feature importance for LO, but are strongly positively correlated (Spearman’s $\rho = 0.92$, see Supplementary Fig. S1). We therefore only selected $T_{p,med}$ to include the wave period, but leave room for other indicators in the clustering. This selection is not meant to imply that the indicators which were not selected are unimportant. However, we opted to limit the clustering to six indicators per species, to reduce the dimensionality of the analysis.

The performance scores of the random forest (RF) model were similar across the five species, with a mean F1 score of 0.9 (standard deviation 0.01). Such high scores may point to a slightly over-fitted model. We deemed this acceptable, though, given the robustness of our models in the cross-validation (Supplementary Fig. S2) and our aim to understand the patterns present in the underlying data, not predict new, unlabeled Coastgons.

### Patterns in coastal indicators of global sea turtle nesting habitats

**Figure 3:**
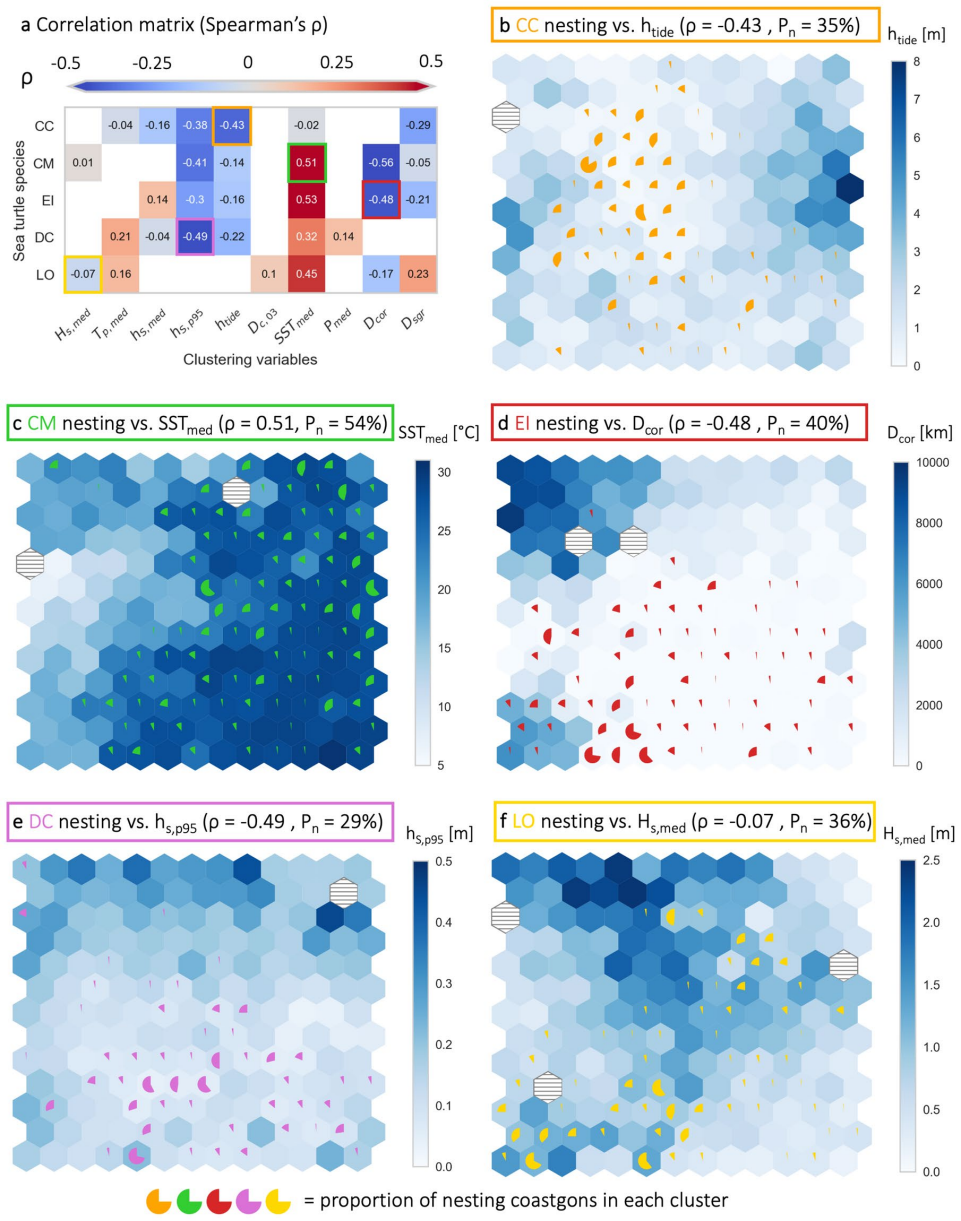
Relationship between coastal indicators and nesting distribution. (**a**) Overview of Spearman rank correlation ($\rho$) between cluster medians (blue scale in panels **b**–**f**) and nesting percentages (pie charts) for each species/indicator pair. Panels (**b**–**f**) visualize the correlation for five species/indicator pairs (highlighted by the colored outlines in the correlation matrix). Each 13-by-13 hexagon lattice shows a separate SOM, created for one species. Each hexagon in the lattices represents a cluster of Coastgons with similar indicators. The clusters are arranged on the lattices based on similarity. Together, the 169 clusters (hexagons) in each lattice represent the global coastline between $-\,39 ^\circ$ and $48 ^\circ$ latitude. The blue color-scale indicates the cluster medians for the given indicator, while the pie charts show the proportion of nesting Coastgons per cluster. Due to the SOM algorithm, some computed clusters may not represent any actual Coastgons (horizontally striped hexagons). Finally, $P_n$ is the percentage of clusters containing any nesting Coastgons per species. All panels created with Python 3.10.8.

To identify patterns in the coastal indicators of global sea turtle nesting habitats, we correlated the SOM cluster medians to the proportion of nesting Coastgons in each cluster using Spearman’s $\rho$ (Fig. 3a). For example, the correlations illustrated in Fig. 3b–f imply that nesting Coastgons are typically found in regions with relatively low tidal ranges (CC), high sea surface temperatures (CM), near coral reef habitats (EI), low extreme surge levels (DC), and across a certain range of wave heights (LO) (see Supplementary Material Section S5 for the SOM lattices of all species/indicator pairs). Although correlation magnitudes were limited to 0.56, several significant relationships emerged. To summarize these, we categorized absolute values of $\rho$ smaller than 0.1 as insignificant, between 0.1 and 0.3 as weak, and larger or equal to 0.3 as significant, revealing the following correlations:

- **Loggerhead turtles (CC)**—Nesting Coastgons significantly correlated negatively with tidal range ($h_{tide}$, Fig. 3b) and extreme surge levels ($h_{S,p95}$). Weak negative correlations were observed with distance to the nearest seagrass habitat ($D_{sgr}$) and median surge levels ($h_{S,med}$). No significant correlations were found with the median wave period ($T_{p,med}$) and sea surface temperature ($SST_{med}$).
- **Green turtles (CM)**—Nesting Coastgons significantly correlated negatively with extreme surge levels ($h_{S,p95}$) and distance to the nearest coral habitat ($D_{cor}$), while weak negative correlations were found with tidal range ($h_{tide}$). Significant positive correlations were observed with sea surface temperature ($SST_{med}$, Fig. 3c). No significant correlation with the median wave height ($H_{s,med}$) and distance to nearest seagrass habitat ($D_{sgr}$).
- **Hawksbill turtles (EI)**—Nesting Coastgons significantly correlated negatively with extreme surge levels ($h_{S,p95}$) and distance to the nearest coral habitat ($D_{cor}$, Fig. 3d), and weak negative correlations were found with distance to the nearest seagrass habitat ($D_{sgr}$) and tidal range ($h_{tide}$). Significant positive correlations were observed with sea surface temperature ($SST_{med}$), and a weak positive correlation with the median surge level ($h_{S,med}$).
- **Leatherback turtles (DC)**—Nesting Coastgons significantly correlated negatively with extreme surge levels ($h_{S,p95}$, Fig. 3e) and a weak negative correlation was observed with the tidal range ($h_{tide}$). Significant positive correlation was found with sea surface temperature ($SST_{med}$) and weak positive correlations with the median wave period ($T_{p,med}$) and median total precipitation ($P_{med}$). No significant correlation found with median surge levels ($h_{S,med}$).
- **Olive ridley turtles (LO)**—Nesting Coastgons showed a weak negative correlation with distance to the nearest coral habitat ($D_{cor}$). Significant positive correlation was observed with sea surface temperature ($SST_{med}$) and weak positive correlations with the median wave period ($T_{p,med}$), distance to ocean currents above 0.3 m/s ($D_{c,03}$), and distance to the nearest seagrass habitat ($D_{sgr}$). No significant correlation found with the median wave height ($H_{s,med}$, Fig. 3f).

### Representative sea turtle nesting regions

**Figure 4:**
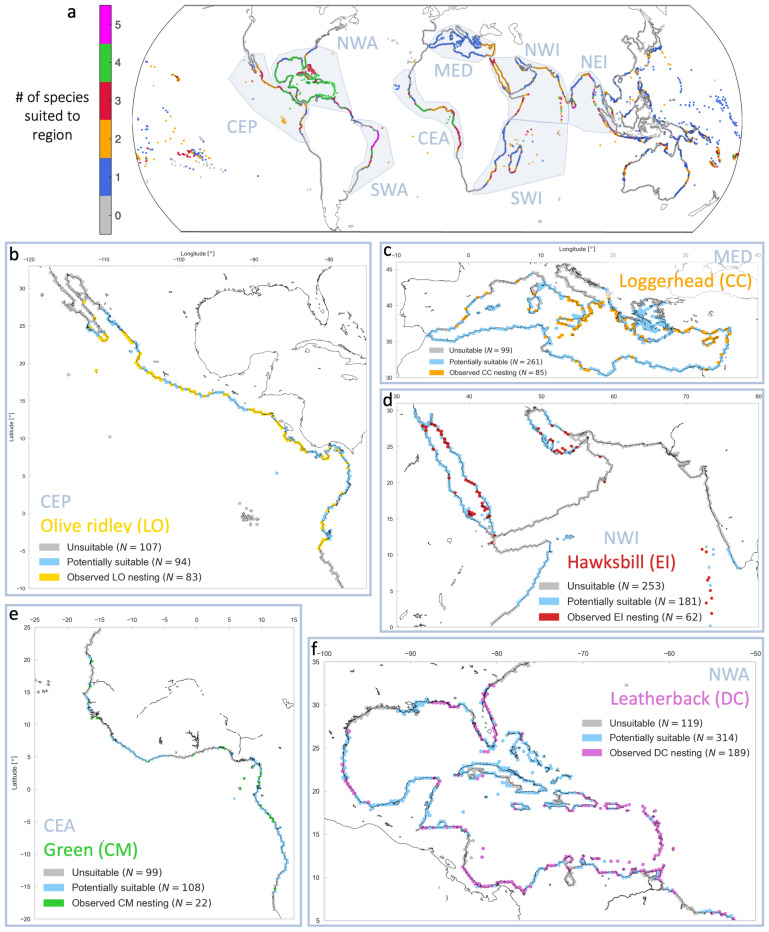
Global overview of sea turtle nesting suitability, based on the selected coastal indicators for each species. (**a**) Global map showing the number of species for which each Coastgon is classified as either ‘Observed nesting’ or ‘Potentially suitable’. The grey-blue polygons show the eight considered regions, inspired by Wallace et al.^51^. Panels (**b**–**f**) show nesting suitability maps for five region/species pairs: (**b**) Olive ridley (LO, yellow) in the Central East Pacific (CEP), (**c**) Loggerheads (CC, orange) in the Mediterranean (MED), (**d**) Hawksbills (EI, red) in the North West Indian (NWI), (**e**) Green turtles (CM, green) in the Central East Atlantic (CEA), and (**f**) Leatherbacks (DC, purple) in the North West Atlantic (NWA). In each map, grey indicates unsuitable Coastgons, light blue denotes potential suitability, and other colors represent observed nesting by the corresponding species. Figure created with Python 3.10.8.

To map representative sea turtle nesting regions, Coastgons were categorized into three categories: observed nesting, potentially suitable (no observed nesting, but part of a cluster with at least 10% observed nesting Coastgons), and unsuitable. We zoomed into eight particular regions, inspired by the regional management units for sea turtles from Wallace et al.^51^ (Fig. 4a): Central East Pacific (CEP), North West Atlantic (NWA), South West Atlantic (SWA), Central East Atlantic (CEA), Mediterranean (MED), North West Indian (NWI), South West Indian (SWI), and North East Indian (NEI). For each region, as well as for the global coastline (all Coastgons), we created nesting suitability maps using the three categories (see examples in Fig. 4b–f), and computed two representative statistics: (1) the percentage of observed nesting Coastgons, $P_{O}$; and (2) the percentage of observed nesting and potentially suitable Coastgons, $P_{S}$ (Table 3). Nesting suitability maps and accompanying box-plots for all region/species pairs are provided in the Supplementary Material, Section S6.

On a global scale, the percentage of suitable Coastgons ranges from $P_{S} = 12.9\%$ for olive ridleys to $P_{S} = 36.9\%$ for green turtles (mean of all species $P_{S} = 23.2\%$). As expected, $P_{S}$ is highest for green turtles, who have the most nesting sites of any species^34^ and are known to nest across a variety of environmental conditions around the world^15^. Suitability percentages are on average 3.3 times higher than observed nesting percentages (mean $P_{O} = 7\%$, ranging from $P_{O} = 4.7\%$ for LO to $P_{O} = 10.1\%$ for CM). This suggests that there are opportunities for turtles to expand their global nesting habitats.

Among individual regions, the North West Atlantic (which includes the wider Caribbean) emerged as the most suitable region, with an average observed nesting percentage of $P_{O} = 34\%$ and potential suitability of $P_{S} = 82.7\%$, across all species except olive ridleys, who do not nest there (Table 3). The Central East Atlantic ($P_{S} = 58.4\%$) and Central East Pacific ($P_{S} = 44\%$) also had high average suitability percentages. The Mediterranean is quite unique in that it currently only supports loggerheads (CC, $P_{O} = 19.1\%$), and to a lesser extent green turtles (CM, $P_{O} = 3.1\%$). The Indian Ocean regions are generally suited to all species, although with lower suitability percentages than other regions ($26.8\%< P_S < 34.1\%$). Finally, the South West Atlantic also had relatively low observed nesting and suitability percentages ($P_{O} = 7.1\%$ and $P_{S} = 35.2\%$), although a large stretch of the Brazilian coastline appears suitable for all five species (Fig. 4a).

There were also significant differences across species. For loggerheads (CC), the North West Atlantic ($P_{S} = 88.7\%$) and Mediterranean ($P_{S} = 77.8\%$) clearly emerged as the most suitable (and most used) regions. For green turtles (CM), suitability percentages were high across regions, with $P_{S} > 35\%$ for all regions except the Mediterranean. Hawksbills (EI) also had high $P_{S}$ values for most regions (they do not nest in the Mediterranean), with the North West Atlantic a clear favorite ($P_{S} = 74.8\%$). Leatherbacks (DC) appeared to be more suited to the Pacific and Atlantic regions, with consistently higher $P_{S}$ values than the Indian Ocean regions. Finally, olive ridley (LO) nesting suitability was highest for the Central East Pacific ($P_{S} = 62.3\%$) and Central East Atlantic ($P_{S} = 72.1\%$). Consistent with the results from the RF model, olive ridleys appeared to have more unique nesting preferences compared to the other four species.

**Table 3:** Overview of the two computed statistics for regional and global nesting suitability.

	CC		CM		EI		DC		LO		Mean	
Region	$P_O$	$P_S$	$P_O$	$P_S$	$P_O$	$P_S$	$P_O$	$P_S$	$P_O$	$P_S$	$P_O$	$P_S$
Central East Pacific	–	–	14.1	39.1	9.2	34.5	12	40.1	29.2	62.3	16.1	44.0
North West Atlantic	28.8	88.7	35.5	86.3	41.2	74.8	30.4	80.9	–	–	34	82.7
South West Atlantic	10.7	40.7	6	37.3	6.7	22.7	5.3	36.0	6.7	39.3	7.1	35.2
Central East Atlantic	–	–	9.6	56.8	7	33.2	17.5	71.6	16.6	72.1	12.7	58.4
Mediterranean	19.1	77.8	3.1	12.6	–	–	–	–	–	–	11.1	45.2
North West Indian	0.6	12.1	11.7	54.6	12.5	49.0	–	–	9.3	20.8	8.5	34.1
South West Indian	5.3	17.3	13.2	74.5	6.2	23.9	2.9	9.5	1.6	8.6	5.8	26.8
North East Indian	2.2	6.4	7.7	39.0	8.6	35.8	6.4	21.7	16.9	39.0	8.4	28.4
Global	6.4	21.9	10.1	36.9	8.2	23.9	5.7	20.3	4.7	12.9	7	23.2

Rows represent regions (see geographical overview in Fig. 4a) and columns show the percentage of observed nesting coastgons ($P_O$) and the percentage of observed nesting and potentially suitable coastgons ($P_S$) for each species and averaged per region.

**Figure 5:**
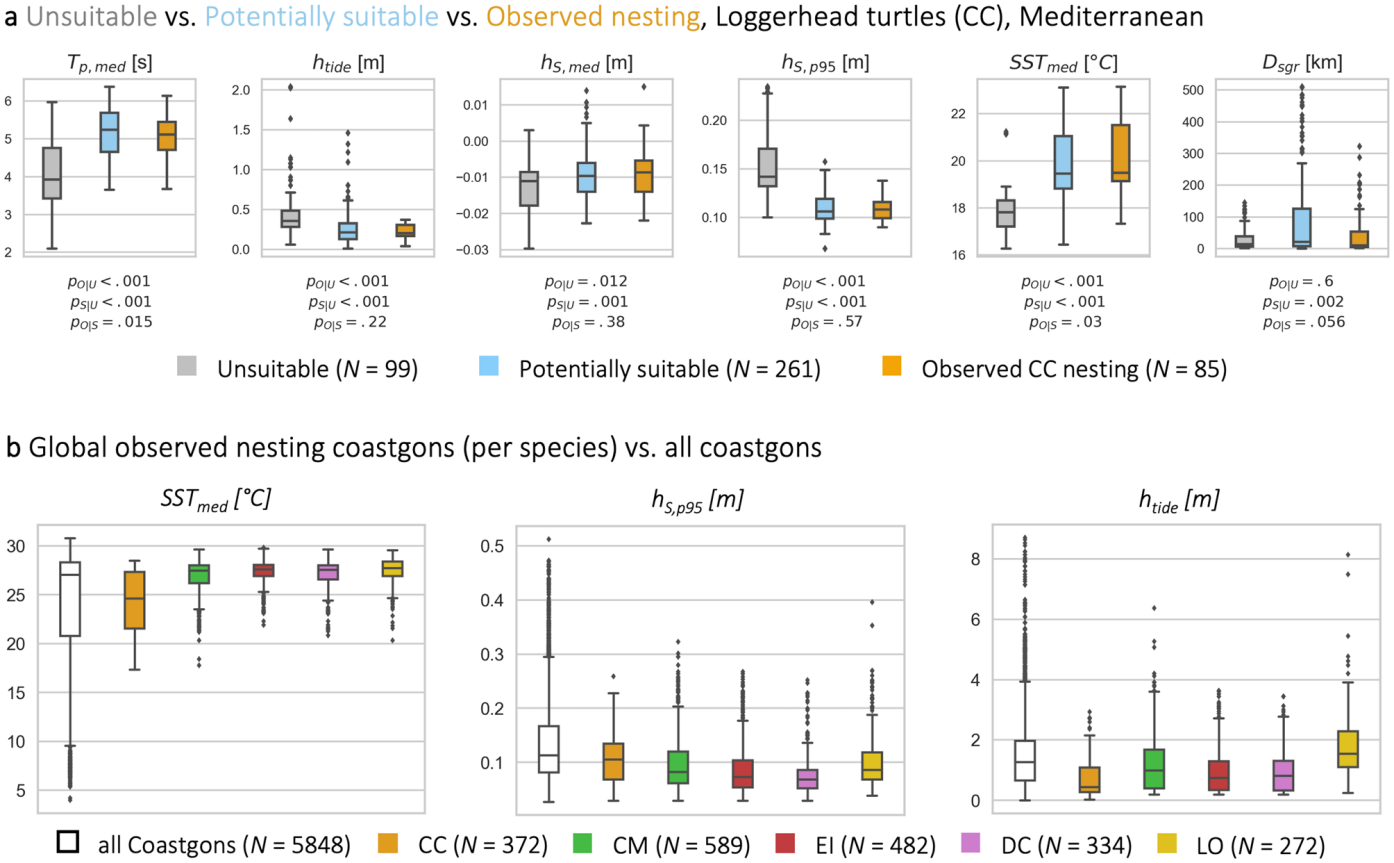
Box-plots showing the distribution of several indicators over sea turtle nesting regions. (**a**) Box-plots for the six indicators selected for loggerhead turtles (from left to right: $T_{p,med}$, $h_{S,med}$, $h_{s,p95}$, $SST_{med}$, $h_{tide}$, $D_{sgr}$) for the three suitability categories along the Mediterranean coastline (Fig. 4): unsuitable (‘U’, grey), potentially suitable (‘S’, blue), and observed nesting (‘O’, orange). Below each box-plot are the p-values from two-sample KS tests between the three category pairs, denoted by their subscript (e.g., $p_{O|S}$ is the p-value between the observed nesting and potentially suitable categories). (**b**) Box-plots for $SST_{med}$ (left), $h_{S,p95}$ (middle), and $h_{tide}$ (right) for all Coastgons (grey) and for the observed nesting Coastgons of each individual species (colors). Boxes represent the interquartile range and whiskers extend to a maximum of 1.5 times the interquartile range. Coastgons beyond the whiskers are shown as markers. Figure created with Python 3.10.8.

To give an example for a regional analysis, in the Mediterranean (nesting map in Fig. 4c), ‘observed nesting’ ($N = 85$) and ‘potentially suitable’ ($N = 261$) Coastgons for loggerhead turtles (CC) generally exhibit higher sea surface temperatures ($17< SST_{med} < 23.5 ^\circ C$) and median wave periods ($3.5< T_{p,med} < 6.3 s$), and lower tidal ranges ($h_{tide} < 0.5 m$) and extreme surge levels ($h_{s,p95} < 0.15 m$) compared to ‘unsuitable’ ($N=99$) Coastgons (Fig. 5a). The p-values of the two-sample KS tests for comparisons between the ‘observed nesting’ and ‘potentially suitable’ categories ($p_{O|S}$) were above the 0.05 threshold for $h_{tide}$, $h_{S,med}$, $h_{s,p95}$ and $D_{sgr}$, and just below 0.05 for $SST_{med}$ and $T_{p,med}$, indicating insignificant or marginal statistical differences for these indicators. In contrast, most p-values for KS-tests involving the ‘unsuitable’ category ($p_{O|U}$ and $p_{S|U}$) were multiple orders of magnitude smaller ($p \ll 0.001$), indicating strong statistical differences with the suitable and observed Coastgons (except for $D_{sgr}$). Based on these indicators, approximately $78\%$ of Mediterranean Coastgons could potentially be suitable for nesting Loggerheads, while nesting has only been observed in 19% of Coastgons (Table 3). These results agree with recent reports of a loggerhead nesting range expansion towards the western Mediterranean, with most newly reported nesting sites lying within Coastgons classified as potentially suitable^52^.

## Discussion

The analysis of 22 regional coastal indicators on the distribution of global sea turtle nesting shows that, in line with previous studies^18,19^, sea surface temperature can be one of the most important indicators for the presence of all five species in a Coastgon. The majority of nesting grounds of all species except loggerheads are situated in regions at the upper end of the analyzed temperature range ($SST_{med} > 25 ^\circ C$; Fig. 5b). Loggerheads (CC) also colonize cooler nesting regions ($SST_{med} > 17 ^\circ C$), like the western Mediterranean and Japan, though their distribution is still influenced by temperature. In fact, had we not filtered out the World’s coldest regions by limiting the Coastgon grid to latitudes between $-39 ^\circ$ and $48 ^\circ$, $SST_{med}$ would have likely been more dominant in the RF model. Distance to the nearest coral reef habitat ($D_{cor}$) was another important indicator, although it is unclear how strongly this is biased by the negative correlation between $SST_{med}$ and $D_{cor}$ ($\rho = -\,0.65$, Supplementary Fig. S1). It would make sense for hawksbills to nest near coral reefs, which are their foraging habitats, but other species have not necessarily been linked to coral reefs in literature. Distance to seagrass ($D_{sgr}$) was mainly important for CC, EI, and LO, and notably less so for green turtles, who forage in seagrass habitats^53^ (although it still ranked sixth).

It has often been hypothesized that sea turtles try to place their nests on the beach by finding a balance between a high nest elevation against flooding, and the distance to the shoreline against predation^19^. While the spatial resolution of our study precludes conclusions regarding nest placement or predation patterns, our results suggest that individual species tend to favor nesting regions with relatively low extreme surges ($h_{S,p95} < 0.25$ m for all species) and low tidal ranges ($h_{tide} < 2.5$ m for CC, EI, and DC), possibly as a strategy to minimize the risk of nest flooding (Fig. 5b). While areas with higher extreme surges can also support nesting—as evidenced by nesting beaches in cyclone-prone regions^13^—our results indicate a significant bias towards low extreme surge environments. This bias might be enhanced by the fact that GTSMv3.0, our source for surge levels, slightly underestimates tropical cyclones, though^54^.

Our global-scale analysis also presented different findings from several relationships previously reported in literature. For instance, Santana Garcon et al.^27^ found that nesting grounds around Australia are generally more exposed to wind and waves than non-nesting beaches. We only found a weak (though not negligible) influence of the median 10m-wind speed, for three species (CM, DC, and EI; Fig. 2). Moreover, despite Putman et al.^28^ reporting a strong relationship between loggerhead nesting activity and distance to the Gulf Stream along the US east coast, we only observed a weak influence from the distance of strong ocean currents. Total precipitation also had comparatively low feature importance in the RF model, contrary to findings of others^18,20^. Perhaps most notably, no geophysical indicators were identified as important in our results. This suggests that patterns resulting from local- or regional-scale analyses do not necessarily reflect large-scale patterns and may, for instance, be confounded by factors correlated with the tested predictors.

Indicators that were not identified as important in our global-scale analysis may still influence turtle nesting for individual regions and sub-populations, or on smaller spatial scales. Our study required the use of global datasets with limited resolution and accuracy. Particularly, local topographic and bathymetric indicators remain difficult to measure, hence available datasets often lack accuracy^55,56^. For example, although Luijendijk et al.^57^ quantified the occurrence of sandy beaches along the global coastline from satellite images, we did not include this data, as their method struggled to detect narrow beaches, particularly in tropical regions with large amounts of vegetation—where many sea turtles nest^11^. Furthermore, aggregating the data onto the Coastgon grid effectively smoothed out local geomorphological features (e.g., embayed beaches), likely contributing to the low importance of the geophysical indicators in the RF results. Hydrodynamic indicators such as waves and water levels, on the other hand, are easier to quantify and likely more consistent over larger spatial scales, and may thus be better suited to large-scale analyses. Hence, we expect geophysical indicators to be more influential at the scale of individual beaches, in line with previous studies (e.g.,^15,21–23^). However, more regional analyses combined with high-resolution, accurate datasets are needed to prove this.

To assess the variability of the GCC transect-based data within each Coastgon, we computed the median absolute deviation from the median, normalized by the median itself (*MADm*)—a measure of variability that is more robust than the commonly used coefficient of variation (standard deviation normalized by the mean)^58^. For example, $MADm = 1$ implies that 50% of the samples differ from the median by more than the median itself. We did this for three geophysical indicators ($\beta _{ns,med}$, $\beta _{bs,med}$, and $z_{max,med}$) and one hydrodynamic indicator (mean higher high water from GCC, extracted from the nearest GTSMv3.0 node for every transect). It should be noted that this variability is a result of both the variability in the indicators, as well as the variable length of the coastline in each Coastgon (i.e., the number of transects linked to each Coastgon). Variability within Coastgons was significantly higher (up to $MADm = 1$) for $\beta _{ns,med}$, $\beta _{bs,med}$, and $z_{max,med}$, than for mean higher high water ($MADm < 0.1$ for $97\%$ of Coastgons). We still included the geophysical indicators in the RF model though, because their variability was also lower for a significant number of Coastgons ($MADm < 0.4$ for $30\%$ of Coastgons). One way to improve our analysis could be to only use sandy coastline transects to derive the geophysical indicators, for example through an updated version of Luijendijk et al.^57^.

Some of the influential indicators selected from the RF model did not show significant correlations in the cluster analysis (Fig. 3). This does not mean that these indicators are not influential but may be explained from the fact that Spearman’s correlation coefficient is designed to detect monotonic relationships (e.g., the lower the extreme surge levels the higher the chance for nesting leatherback turtles). If nesting grounds fall within a specific range of a given indicator, however, this relationship is not readily identified through correlation. The SOM lattices and box-plots allow a quick visual inspection of the distribution of nesting grounds for each indicator, but it remains difficult to quantify such non-monotonic relationships. For example, global loggerhead (CC) nesting Coastgons are limited to a median sea surface temperature range of $17-29\,\,^\circ$C (Fig. 5b). This range is still in the upper part of the total observed $SST_{med}$ range, but the median $SST_{med}$ for loggerhead nesting Coastgons ($24.5\,\,^\circ$C) is lower than for all Coastgons ($27.5\,\,^\circ$C). Hence no significant rank correlation is observed ($\rho = -\,0.02$; Fig. 3a), even though sea surface temperature clearly constraints the suitability for loggerhead nesting. Nonetheless, such relationships are still captured in the suitability maps, as the SOMs (from which the maps are derived) do identify complex, non-monotonic patterns.

Another way to show the complexity of nesting suitability and the effectiveness of our selected indicators is by examining the percentage of nesting clusters ($P_n$) and their distribution on the SOM lattices (Fig. 3). Ideally, a complete set of indicators would lead to a clear division of nesting Coastgons over suitable clusters, with decreasing nesting percentages around them signaling the limits of suitability. For example, Loggerhead (CC) nesting occurs in $35\%$ of clusters, and the lattice shows one main agglomeration of high nesting percentage clusters, with decreasing percentages around it (Fig. 3b). In contrast, green turtles (CM) nest in $54\%$ of clusters, with many low nesting percentages spread across the lattice (Fig. 3c). A similar pattern can be seen for hawksbills (EI), although the nesting cluster percentage is lower ($P_n = 40\%$; Fig. 3d). While green turtles are particularly known to nest across a very broad range of environmental conditions^15^, these findings underscore the hypothesis that nesting preferences involve a complex interplay of biotic and abiotic factors.

Our nesting suitability maps represent our best estimate of each species’ *fundamental niche* (the environmental range theoretically suitable for nesting^59,60^), based on a set of abiotic environmental characteristics. The observed nesting distribution can be interpreted as an approximation of the *realized niche*, where the species actually nests. The realized niche is typically more constrained than the fundamental niche, due to complex biotic interactions (e.g., predation and recruitment limitation), which are challenging to incorporate into habitat mapping^59^. Moreover, our indicator set, while comprehensive, may still miss some potentially influential abiotic factors (e.g., sandy beach occurrence and grain size characteristics^15^), thereby approximating the fundamental niche.

Given our main goal was to identify characteristics that enable nesting at any scale, we chose not to weight nesting regions based on their population sizes. However, certain rookeries around the globe are hotspots of turtle nesting. For example, Raine Island, Australia for green turtles^61^, Masirah Island, Oman for loggerheads^62^, or the mass nesting sites in Mesoamerica and India, where thousands of olive ridleys nest simultaneously during so-called *arribadas*^63,64^. Future work could benefit from incorporating such high-density nesting grounds into the analysis, which would offer a more nuanced understanding of global patterns. Additionally, we did not consider the seasonality of turtle nesting in the computation of our indicators. Sea turtles are known to venture far away from their nesting grounds outside of the nesting season, hence our analysis might benefit from filtering the time series of hydrodynamic and atmospheric data to reflect conditions during the nesting season.

A challenge for studies like this one is the availability of global sea turtle nesting data. The SWOT database is an invaluable resource for any research related to sea turtle nesting distribution, but also has limitations. Even though it contains data from over 130 countries and territories all over the globe, in some regions data availability and accuracy are limited and dependent on local programs with varying monitoring standards^34^. A significant number of false non-nesting Coastgons can bias the feature importance, as characteristics supportive of nesting are erroneously associated with non-nesting. However, our chosen Coastgon resolution (50 km) helps overcome this challenge by aggregating to regional scales. The RF feature importance remained consistent through the cross-validation, showing the model is robust to relatively small changes in the input data (Supplementary Fig. S2). Therefore, the results of this study can help identify currently undocumented nesting regions, to facilitate more reliable and accurate nesting data in the future. In Somalia, for example, there is currently no (public) data^65^, but all nearby countries support nesting and our suitability maps indicate that parts of its coastline could be suitable for up to four species (Fig. 4a).

Contrary to most previous research on sea turtle nesting characteristics, one of our main motivations behind this study is the eventual design and implementation of nature-based solutions that can help preserve and expand nesting habitats for sea turtles. Consequently, our selection of indicators was driven by a focus on abiotic indicators potentially modifiable by nature-based engineering designs, such as hydrodynamics and geomorphology. Similarly, we adopted a spatial scale that helps identify coastal regions where nature-based solutions may be suitable. Moving forward, we aim to leverage the findings of this study to identify and map coastal hazards threatening global sea turtle nesting habitats (e.g., flooding and erosion of nesting beaches) and to assess the suitability of specific nature-based solutions to mitigate these hazards.

## Conclusion

We examined the relationship between regional coastal characteristics and the global nesting distribution of five sea turtle species (loggerhead, CC; green, CM; hawksbill, EI; leatherback, DC; and olive ridley, LO) to identify suitable nesting conditions and lay a foundation for the design of nature-based solutions to protect and expand global nesting habitats. An initial set of 22 coastal indicators was considered—spanning hydrodynamic, atmospheric, geophysical, habitat, and human processes—on a hexagonal coastline grid (Coastgons) of $\approx 50$ km-resolution. We assessed the influence of these indicators on the global distribution of sea turtle nesting by fitting a random forest model to the data, which returns each indicator’s relative importance in splitting the data into nesting and non-nesting categories. Based on this importance, a subset of six important indicators per species was examined through a SOM-based cluster analysis to reveal patterns in the coastal characteristics of global nesting habitats, and identify new, potentially suitable nesting regions.

While there were differences between species, at the coarse, global scale considered here, important indicators consisted mainly of sea surface temperature ($SST_{med}$), extreme surge levels ($h_{s,p95}$), tidal range ($h_{tide}$), and the distance to the nearest coral reef ($D_{cor}$) and seagrass habitats ($D_{sgr}$). For example, individual species’ nesting grounds tend to occur in regions with relatively low tidal ranges (CC, EI, and DC), low extreme surge levels (CC, CM, EI, and DC), warm temperatures (CM, EI, DC, and LO), and near coral habitats (CM and EI). The first two observations might suggest that sea turtles select their nesting grounds in an effort to reduce the risk of (periodic) nest flooding, as has similarly been hypothesized on smaller spatial scales (e.g.,^15^). Other indicators reported as influential in literature appeared less important according to our results (e.g., precipitation^18^ and wind^27^). Notably we didn’t identify any important geophysical indicators at this scale, but expect these to be more influential for smaller spatial scales and more accurate data.

We identified new, potentially suitable nesting regions, mapping each species’ fundamental nesting niche on global and regional nesting suitability maps. Global nesting suitability ranged from 12.9% (LO) to 36.9% (CM) of Coastgons (mean 23.2%). However, observed nesting currently only occurs in 4.7% to 10.1% of Coastgons (mean 7%), suggesting that the realized niche is still significantly smaller, and that there is potential for sea turtles to expand their nesting habitats. This is a particularly important finding in the face of nature-based solutions and assisted colonization of new coastal regions^32^, as recent studies suggest that phenological shifts (earlier onset of nesting season) are unlikely to be sufficient for most sea turtle populations to stay within appropriate nesting conditions^45,66^.

This study has revealed distinct patterns in the coastal characteristics of global sea turtle nesting habitats and has identified new, potentially suitable, nesting regions for the five globally distributed sea turtle species. Even though sea turtle nesting behavior remains difficult to predict^30^, the results of this study can help identify suitable nesting conditions, quantify potential hazards to global sea turtle nesting habitats, and function as a basis for the design of nature-based solutions to preserve and potentially expand these habitats.

### Supplementary Information

Supplementary Information.

## Data Availability

The Coastgon dataset used in this study, including the 22 indicators, is publicly available through the 4TU.ResearchData repository via this https://data.4tu.nl/datasets/68377ee4-892d-40f0-a490-29f2601e6825. All global datasets used in this study are also available online, for access please refer to the corresponding references mentioned in this article. Additional data on the results (e.g., SOM clusters) can be requested from the corresponding author at j.c.christiaanse@tudelft.nl.
