# Supplementary material for: Distribution of global sea turtle nesting explained from regional-scale coastal characteristics
Source: Sci Rep. 2024 Jan 8;14:752. doi: 10.1038/s41598-023-50239-5 (PMC10774326; doi:10.1038/s41598-023-50239-5)
Supplement: Supplementary file 1 — Supplementary Information. [file 41598_2023_50239_MOESM1_ESM.pdf]

# Supplementary Material

# Distribution of global sea turtle nesting explained from regional-scale coastal characteristics

Jakob C. Christiaanse[1,*], José A. A. Antolínez[1], Arjen P. Luijendijk[1,2], Panagiotis Athanasiou[2], Carlos M. Duarte[3], Stefan Aarninkhof[1]

[1]Department of Hydraulic Engineering, Delft University of Technology, Delft, The Netherlands
[2]Deltares, Delft, The Netherlands
[3]King Abdullah University of Science and Technology (KAUST), Thuwal, Saudi Arabia

[*]Correspondence and requests for materials should be addressed to J.C. (j.c.christiaanse@tudelft.nl)

## Table of Contents Supplementary Material

*S1. Derivation of coastal indicators* ........ 2
*S2. Correlation matrix coastal indicators* ........ 5
*S3. Random Forest cross-validation* ........ 6
*S4. Nesting data overview* ........ 8
*S5. SOM lattices and nesting percentages* ........ 9
*S6. Global and regional nesting suitability maps* ........ 14
*References* ........ 53

## S1. Derivation of coastal indicators

As explained in the main paper, 22 coastal indicators were used in the analysis, divided in five categories: hydrodynamic, atmospheric, geophysical, habitat, and human. Here, we explain the derivation of the indicators for each global dataset we used.

For all indicators, we had to compute a distance metric at some stage, either when selecting the nodes from which to extract data for each Coastgon, or directly for the three distance indicators ($D_{c,03}$, $D_{cor}$, $D_{sgr}$). For all distances, we used the *Haversine* distance, which is shortest distance between two points on the surface of a sphere, given their longitudes and latitudes.

***ERA5 Ocean ($H_{s,med}$, $H_{s,p95}$, $T_{p,med}$, $T_{p,p95}$, $T_{p,std}$)***

The wave climate indicators were derived from ERA5-wave[1], which has a structured grid at a spatial resolution of 0.5° (≈ 55 km at the equator). Since this resolution is similar to the Coastgon resolution (≈ 50 km diameter), we extracted the hourly time series (42 years, 1980–2021) of the significant wave height ($H_s$) and peak wave period ($T_p$) from the closest ERA5 Ocean node to each Coastgon (i.e., we extracted a single time series for $H_s$ and $T_p$ for each Coastgon). We then simply used descriptive statistics (median, standard deviation, and 95th percentile) to derive the five indicators from the time series.

***ERA5 Atmos ($U_{10,med}$, $U_{10,p95}$, $SST_{med}$, $P_{med}$)***

All four atmospheric indicators were derived from ERA5-atmos[1], in similar fashion to the wave indicators. However, since the ERA5 Atmos grid has a higher spatial resolution (0.25° or ≈ 27.5 km at the equator), we extracted hourly time series (42 years, 1980–2021) from the two closest nodes to each Coastgon, instead of only the closest. We again used descriptive statistics (median and 95th percentile) to compute the indicators for each node, and then took the average of the two nodes associated to each Coastgon.

ERA5 provides the wind speed (and direction) as U/V components, so we first transformed the time series of each node from Cartesian to polar coordinates to derive the full wind speed. For precipitation, we used the annual total precipitation as base indicator, so we first computed the sum of the total precipitation for each year, and then took the median of all years to determine $P_{med}$, which has unit *mm/year*.

***GTSM ($h_{S,med}$, $h_{S,p95}$, $h_{tide}$)***

The GTSMv3.0[2] grid has a spatial resolution that varies geographically. Along most of the global coastline, the resolution of the output nodes is between 10–50 km, but for European coastlines it is higher (< 10 km). We decided to use one consistent way of selecting nodes for data extraction, so we used the three closest nodes to each Coastgon.

GTSM provides 10-minute interval time series of various water level components and also computed indicators for the period between 1985–2014 (30 years). We extracted the mean tidal range and the median/95th percentile of the storm surge for each GTSM node. For each Coastgon, we again averaged the obtained values of the three assigned nodes to yield one value per Coastgon.

***ORAS5 ($u_{c,med}$, $D_{c,03}$)***

ORAS5[3] provides monthly mean values of current velocities over a 0.25° resolution grid, at varying depth intervals. We extracted time series (42 years, 1980–2021) of the current velocity at 0.5 m depth. Velocities are provided in zonal and meridional components, so, as for the wind speeds in ERA5, we transformed the time series from Cartesian to polar notation to obtain the full current velocity. We then computed $u_{c,med}$ as the average of the median monthly current velocities of the three nodes assigned to each Coastgon. As a metric for the proximity of strong ocean currents, we computed the distance (Haversine) of each Coastgon to the nearest ORAS5 node where the median monthly current velocity was larger than 0.3 m/s.

***GCC ($\varphi_{std}$, $\beta_{ns,med}$, $\beta_{bs,med}$, $z_{max,med}$, $z_{max,std}$, $p_{built}$)***

The coastline transects of the GCC dataset[4] were already used to create the Coastgon grid (see methods section of the main paper), so each GCC transect was associated with one Coastgon (transects further than 100 km from any Coastgon were ignored in the analysis). So, for each of geophysical indicators we used descriptive statistics to compute the median/standard deviation over all transects linked to each Coastgon. For the percentage of built environment, we used the mean instead of the median, to obtain the total percentage of built environment for each Coastgon.

***Allan Coral Atlas and UNEP-WCMC ($D_{cor}$, $D_{sgr}$)***

The Allan Coral Atlas[5] and the UNEP-WCMC seagrass dataset[6] provide geo-referenced locations of coral reefs and seagrass meadows respectively. We computed the distance (Haversine) from each Coastgon to the nearest coral reef or seagrass meadow from the two datasets.

We first overlaid the H3 global hexagonal grid resolution 5 over the coral reef / seagrass polygons. The resolution 5 H3 hexagons have a diameter of ≈ 15 km, which is one level higher than resolution 4 (used to create the Coastgon grid). We did this because our algorithm computes the distances between the coastline centroids of the Coastgons and the centroids of the coral reef / seagrass polygons. Since some of these polygons cover large spatial areas, their centroids can sometimes be several hundred kilometers away from the border of the polygon, giving a wrong representation of the actual distance between a Coastgon and the nearest coral reef / polygon. By overlaying the resolution 5 H3 grid over the polygons, we create a layer of approximately equal size hexagons that cover the same areas as the original polygons (up to the ≈ 15-km-resolution of the H3 grid). Therefore, the distance between the Coastgon and the nearest coral reef / seagrass hexagon is never further away than the hexagon radius (≈ 6−10 km). Additionally, the computational cost of this approach is less, as very small coral reef / seagrass polygons that are grouped together the H3 hexagons, leading to a smaller total number of polygons.

***Challenges with distance indicators***

We had to constrain the three distance indicators ($D_{c,03}$, $D_{cor}$, $D_{sgr}$) to certain ocean basins to avoid distances that cross continents and are therefore much shorter than they would be through the Ocean. For relatively short island crossings ($O(10km)$) this is negligible, given the scale and resolution of our analysis, but it becomes problematic in regions like the Mediterranean, for example. When simply using a closest distance approach, the eastern Mediterranean would be

closest to the coral reefs of the Red Sea. Similarly, the nearest coral reefs to some parts of the Pacific Central American coast would be in the Caribbean. Assuming that sea turtles do not actively cross the Suez or Panama Canals to change ocean basins, however, these distances are not appropriate for our analysis. We therefore computed distances within two main basins: an Atlantic basin (including the Mediterranean, Gulf of Mexico, and Carribean) and an Indo-Pacific basin, covering the rest of the World's oceans. This way, the closest coral reefs to the Mediterranean, for example, are in the Caribbean.

This approach has worked well for $D_{c,03}$ and $D_{sgr}$. However, for $D_{cor}$ some relatively large land crossings remained in the South West Atlantic (Argentina, Uruguay, Brazil) and Southern Australia. Furthermore, at the border of the Atlantic and Indo-Pacific basins (near Capetown), the closest coral reefs are in southern Mozambique (Indo-Pacific basin), approximately 2000 km northwest of Capetown. The Coastgons in the Atlantic basin, however, were linked to the coral reefs in the Caribbean, some 9500 km away. We mitigated these challenges by recomputing $D_{cor}$ using the Searoute python package, which computes the shortest maritime distance between two points (i.e., only crossing through the sea):

- For the southern west coast of Africa (Ivory Coast to South Africa), we computed $D_{cor}$ as the minimum out of the Haversine distance to the nearest Caribbean corals and the Searoute distance to the nearest coral in southern Mozambique.
- For the East coast of South America (Brazil, Uruguay, Argentina), we used the Searoute distance to the nearest Caribbean coral reef.
- For the South coast of Australia, we used the smaller of the two Searoute distances to the nearest corals off of the east and west coasts of Australia.

## S2. Correlation matrix coastal indicators

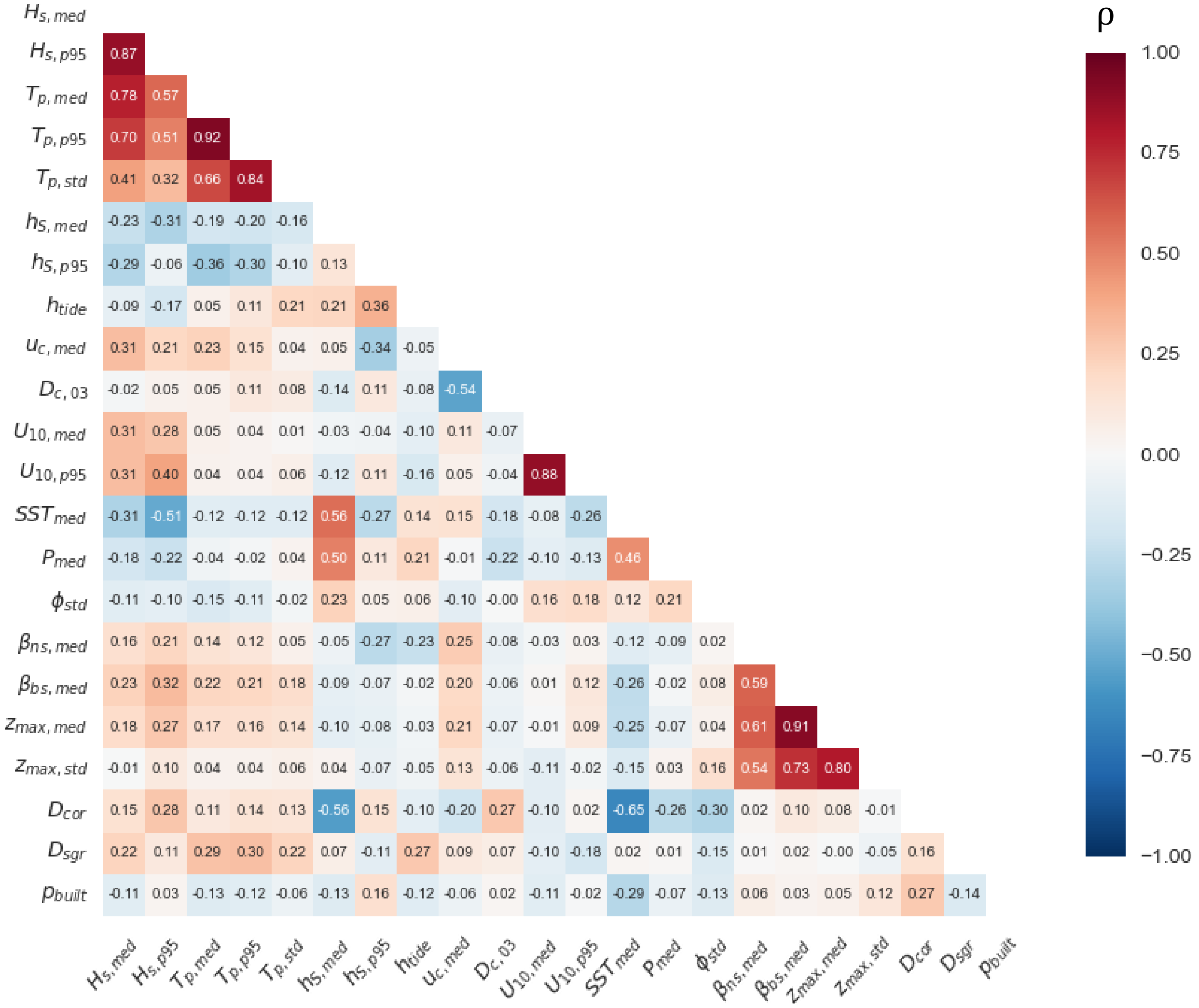


*Figure S1: Matrix plot showing Spearman's rank correlation (ρ) between each pair of the 22 coastal indicators (see Table 2 in the main paper). Red (blue) colors indicate positive (negative) correlations. Figure created with [Python 3.8.10](Python 3.8.10).*

## S3. Random Forest cross-validation

To test the robustness of the RF model used to select the indicators for the clustering, we carried out a four-fold cross validation: we split the data into four equally sized partitions (each containing 25% of the original data) and trained four RF ‘sub-models’, each on a unique combination of three out of the four partitions (75% of the data). Since the dataset has imbalanced classes (i.e., there are much less nesting Coastgons than non-nesting), we used a stratified split, meaning each individual partition had approximately the same percentage of nesting and non-nesting Coastgons as the full dataset.

As we are not doing prediction with the RF model, it would make little sense to test each sub-model’s predictive capability on the ‘unseen’ 25% (as would be the conventional cross-validation procedure for predictive regression). Rather we compared the RF feature importance of the four sub-models with the one trained on the full dataset. As can be seen in Figure S2, both the feature importance values and the ranks are very consistent between the five models within each species (4 trained on the subsets and 1 on the entire dataset), and the same dominant variables stand out. This shows that the RF model is robust to (relatively small) changes in the input data.

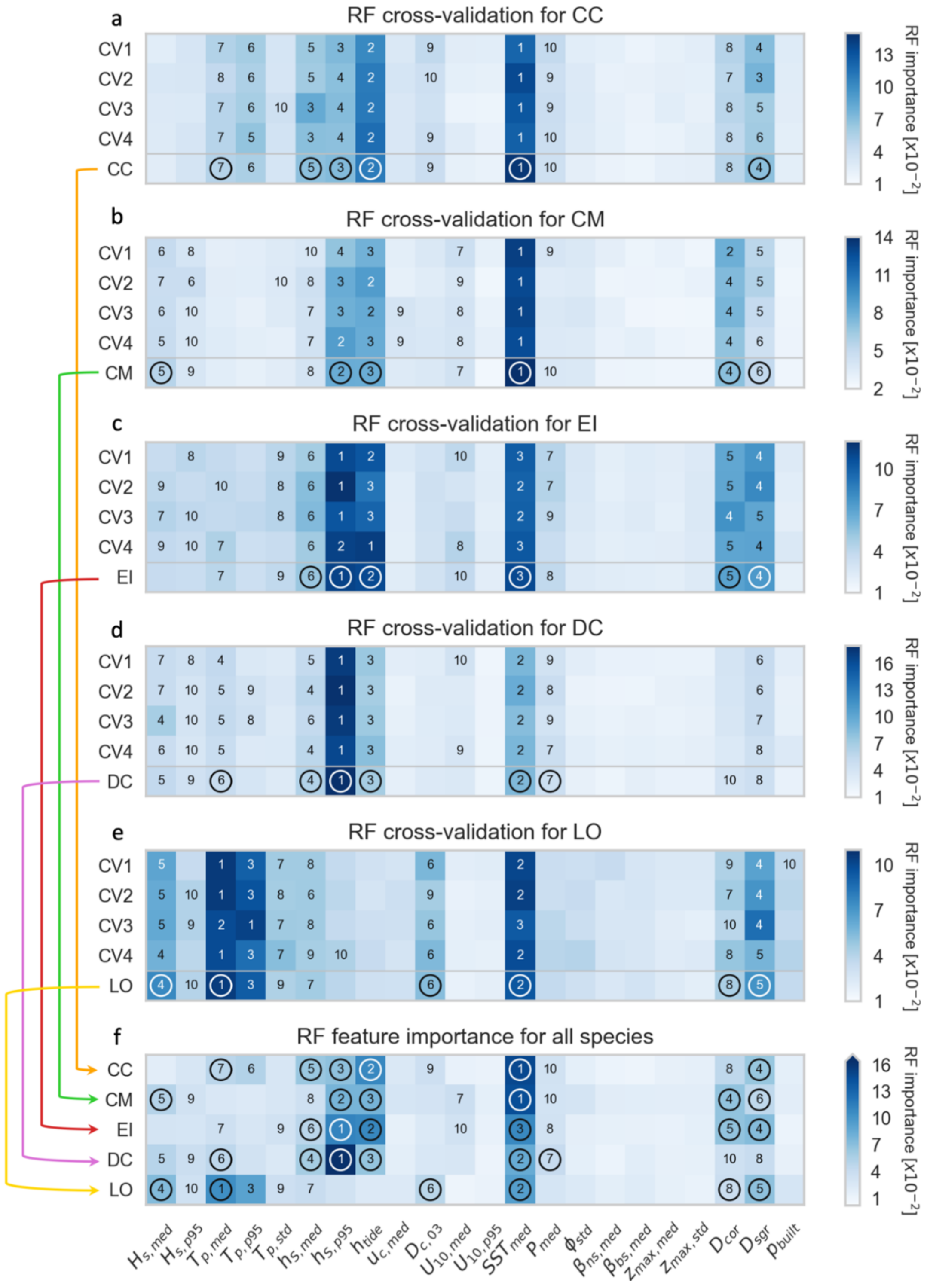


*Figure S2: Cross-validation of the RF model for each species. For each species, we split the data into four equal-size partitions and trained four 'sub-models', each on a unique 75% of the original data. The full model was trained on the full dataset. Panels a-e show the cross-validation results for each species: feature importance (blue scale) of the four sub-models on the first four rows (CV1 to CV4) and of the main model on the last row (denoted by species abbreviation). Panel f is identical to Figure 2 in the manuscript—it summarizes the full RF models for the five species, so its rows are equivalent to the last row of each panel a-e (marked by the coloured arrows). The numbers show the ranks of the ten most important indicators and the circles show the selected indicators for each species, based on the full RF models. Figure created with Python 3.8.10.*

S4. Nesting data overview

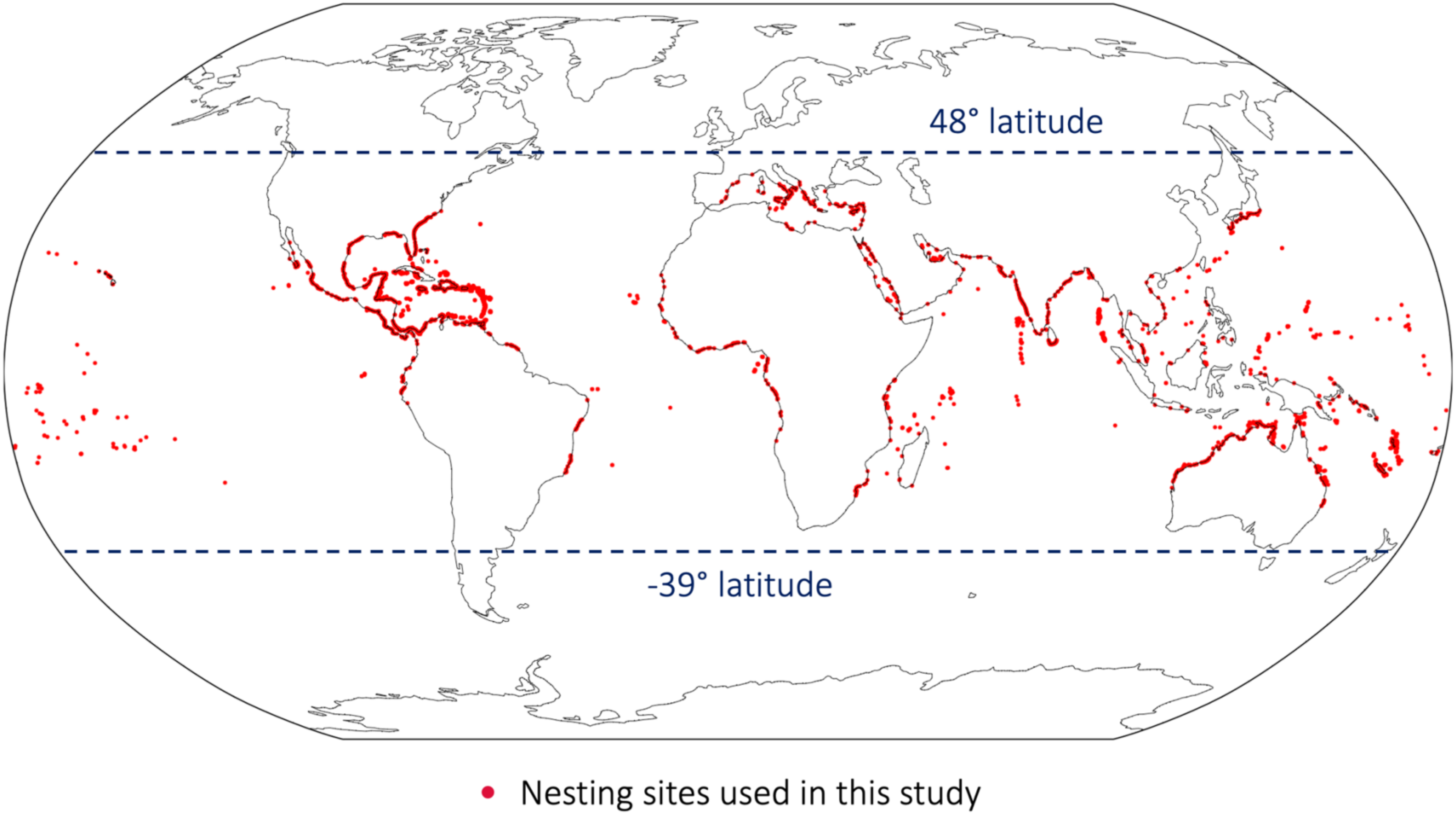


*Figure S3: Overview of all nesting sites of all species used for this study shown as red dots. The dashed lines show the latitude limits applied to the Coastgon grid.*[9]

## S5. SOM lattices and nesting percentages

This section contains all SOM lattices and nesting percentages for all species, completing the ones in Figure 3 of the main paper.

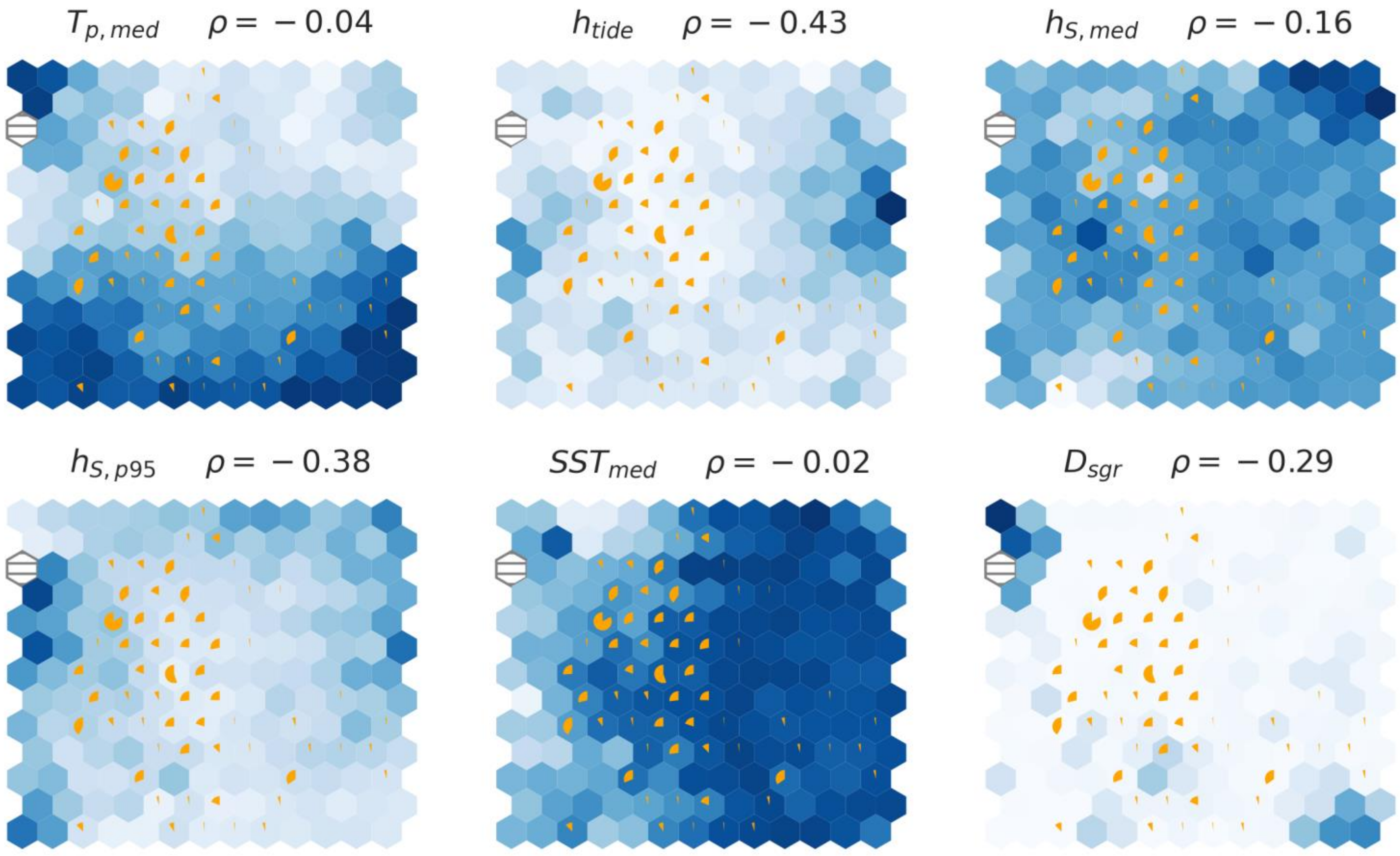


*Figure S4: SOM cluster lattices for the six indicators selected for loggerhead turtles (CC). The blue color scale indicates the cluster medians for the corresponding indicator (relative value, dark is highest, light is lowest). The orange pie charts show the proportion of nesting Coastgons in each cluster. The title of each subplot shows the indicator and the Spearman correlation (ρ) between the cluster medians and the nesting percentages. Due to the SOM algorithm, some computed clusters may not represent any actual Coastgons (indicated by horizontally striped hexagons).*

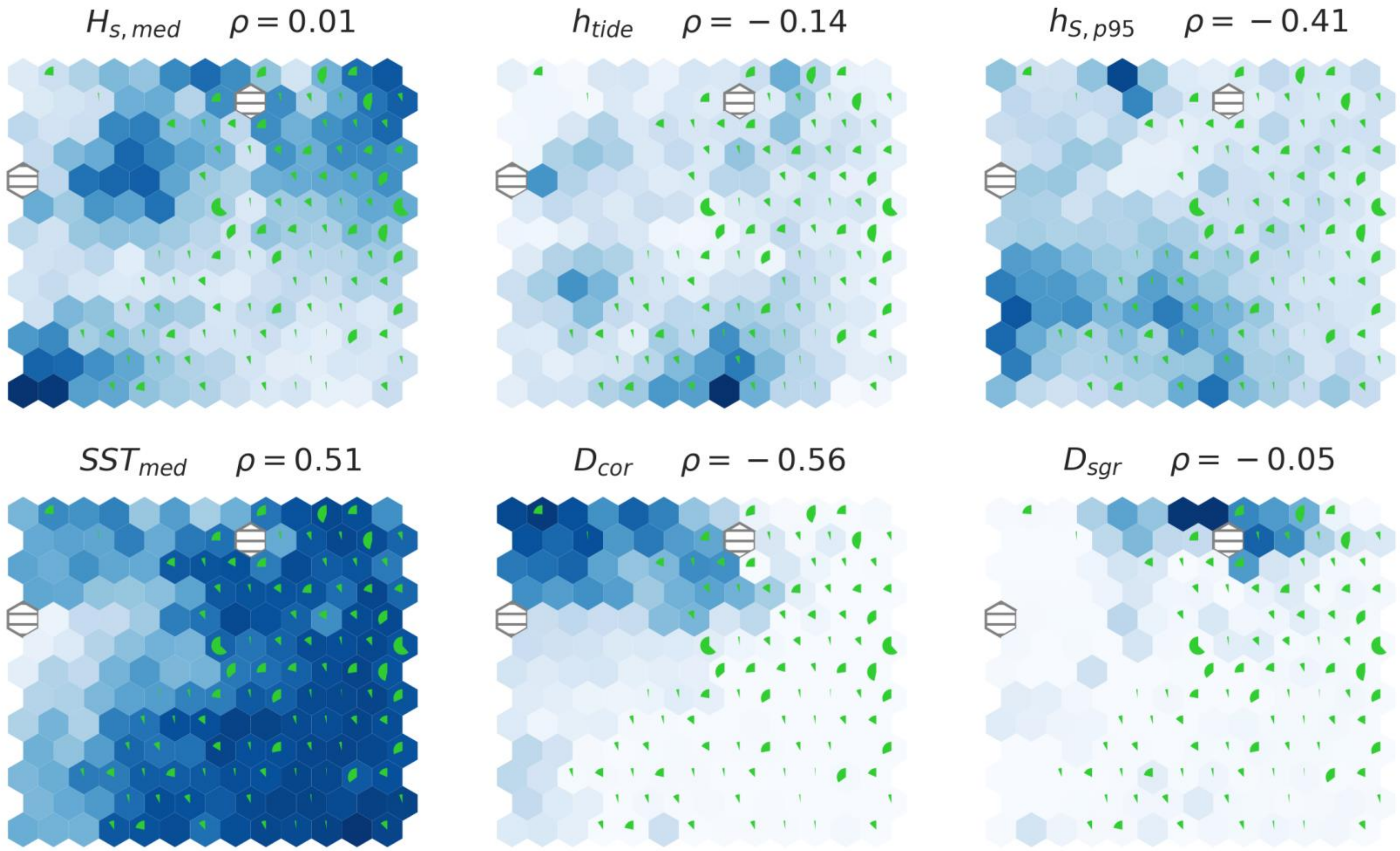


*Figure S5: SOM cluster lattices for the six indicators selected for green turtles (CM). The blue color scale indicates the cluster medians for the corresponding indicator (relative value, dark is highest, light is lowest). The orange pie charts show the proportion of nesting Coastgons in each cluster. The title of each subplot shows the indicator and the Spearman correlation (ρ) between the cluster medians and the nesting percentages. Due to the SOM algorithm, some computed clusters may not represent any actual Coastgons (indicated by horizontally striped hexagons).*

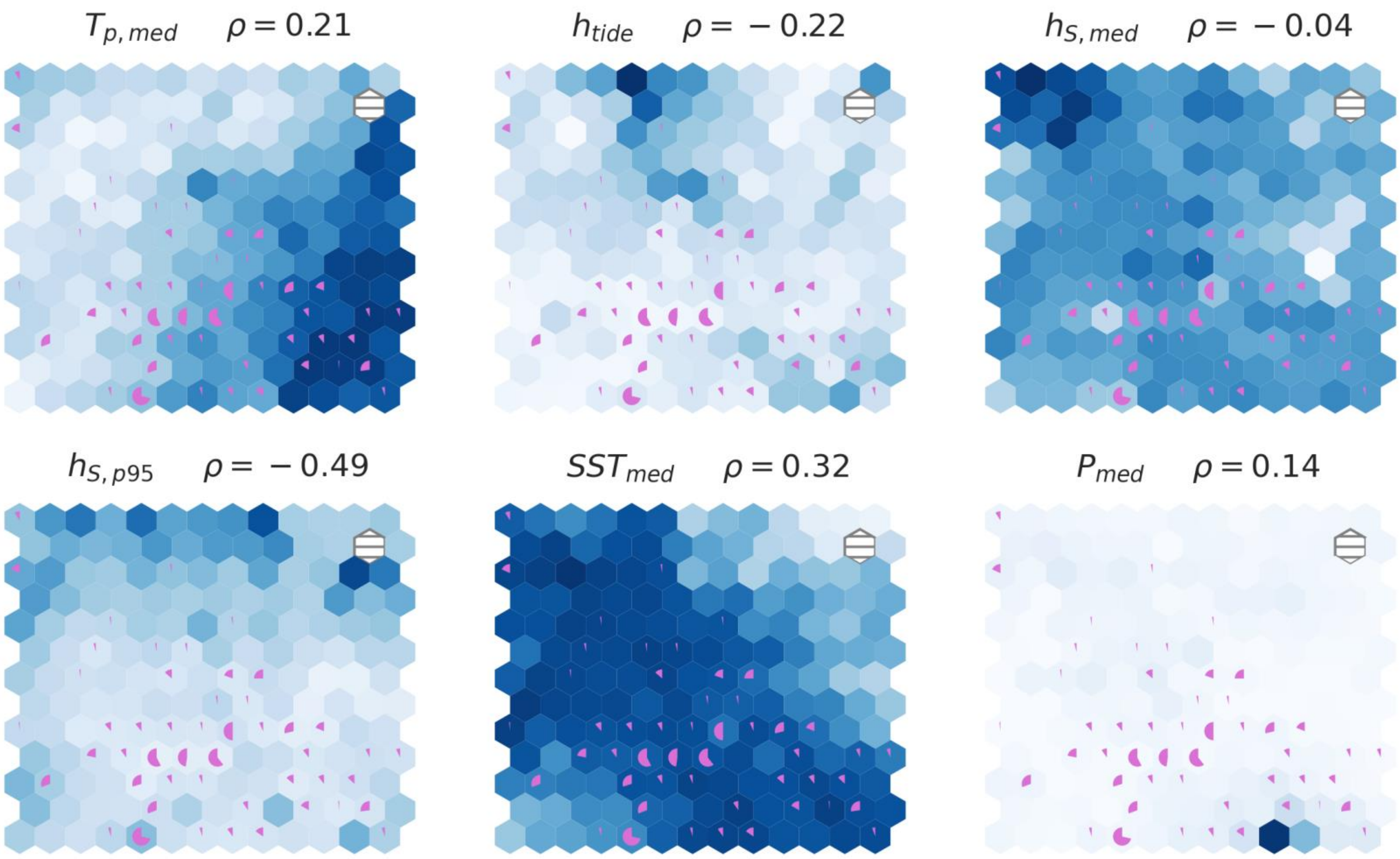


*Figure S6: SOM cluster lattices for the six indicators selected for leatherback turtles (DC). The blue color scale indicates the cluster medians for the corresponding indicator (relative value, dark is highest, light is lowest). The purple pie charts show the proportion of nesting Coastgons in each cluster. The title of each subplot shows the indicator and the Spearman correlation (ρ) between the cluster medians and the nesting percentages. Due to the SOM algorithm, some computed clusters may not represent any actual Coastgons (indicated by horizontally striped hexagons).*

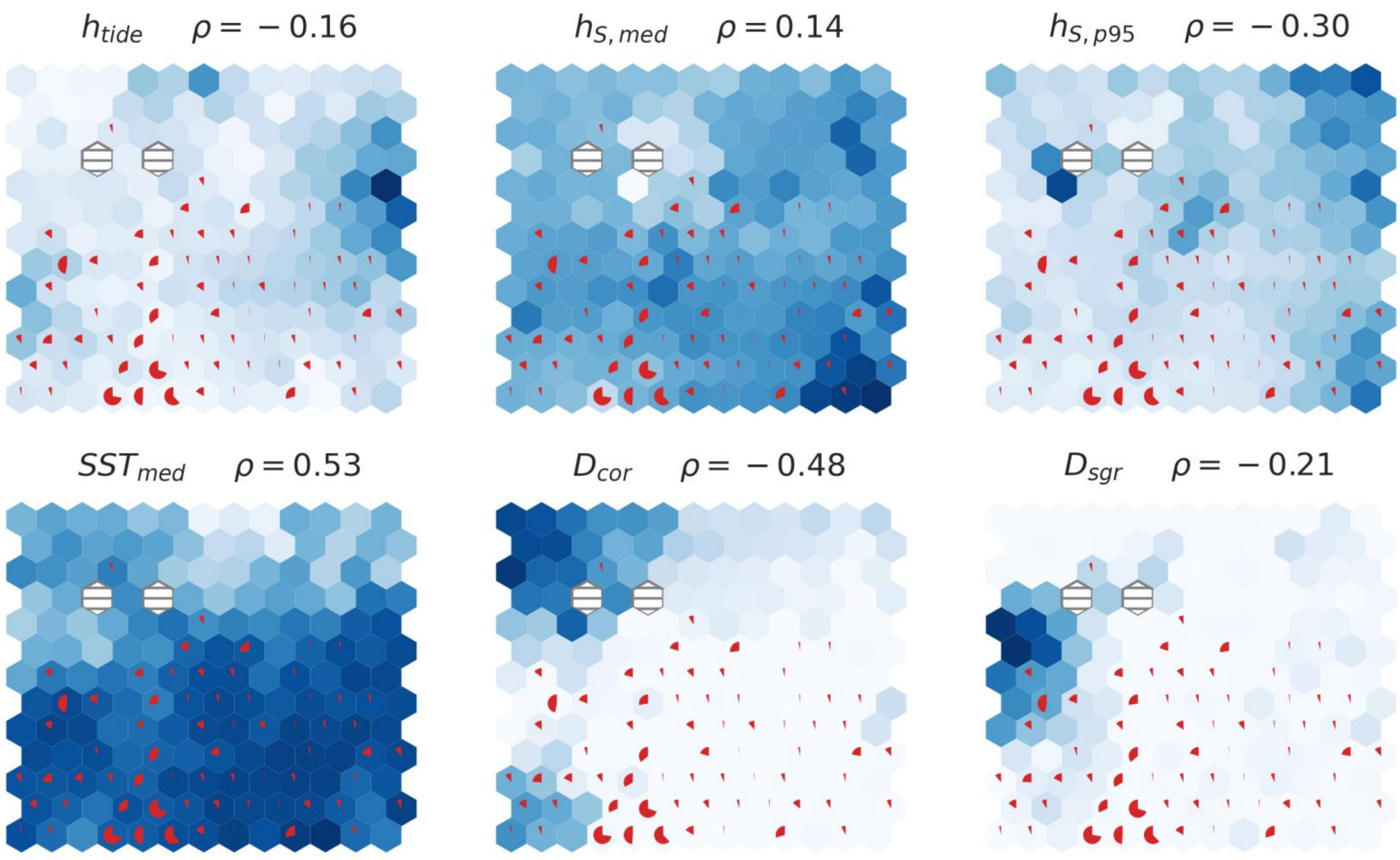


*Figure S7: SOM cluster lattices for the six indicators selected for hawksbill turtles (EI). The blue color scale indicates the cluster medians for the corresponding indicator (relative value, dark is highest, light is lowest). The red pie charts show the proportion of nesting Coastgons in each cluster. The title of each subplot shows the indicator and the Spearman correlation (ρ) between the cluster medians and the nesting percentages. Due to the SOM algorithm, some computed clusters may not represent any actual Coastgons (indicated by horizontally striped hexagons).*

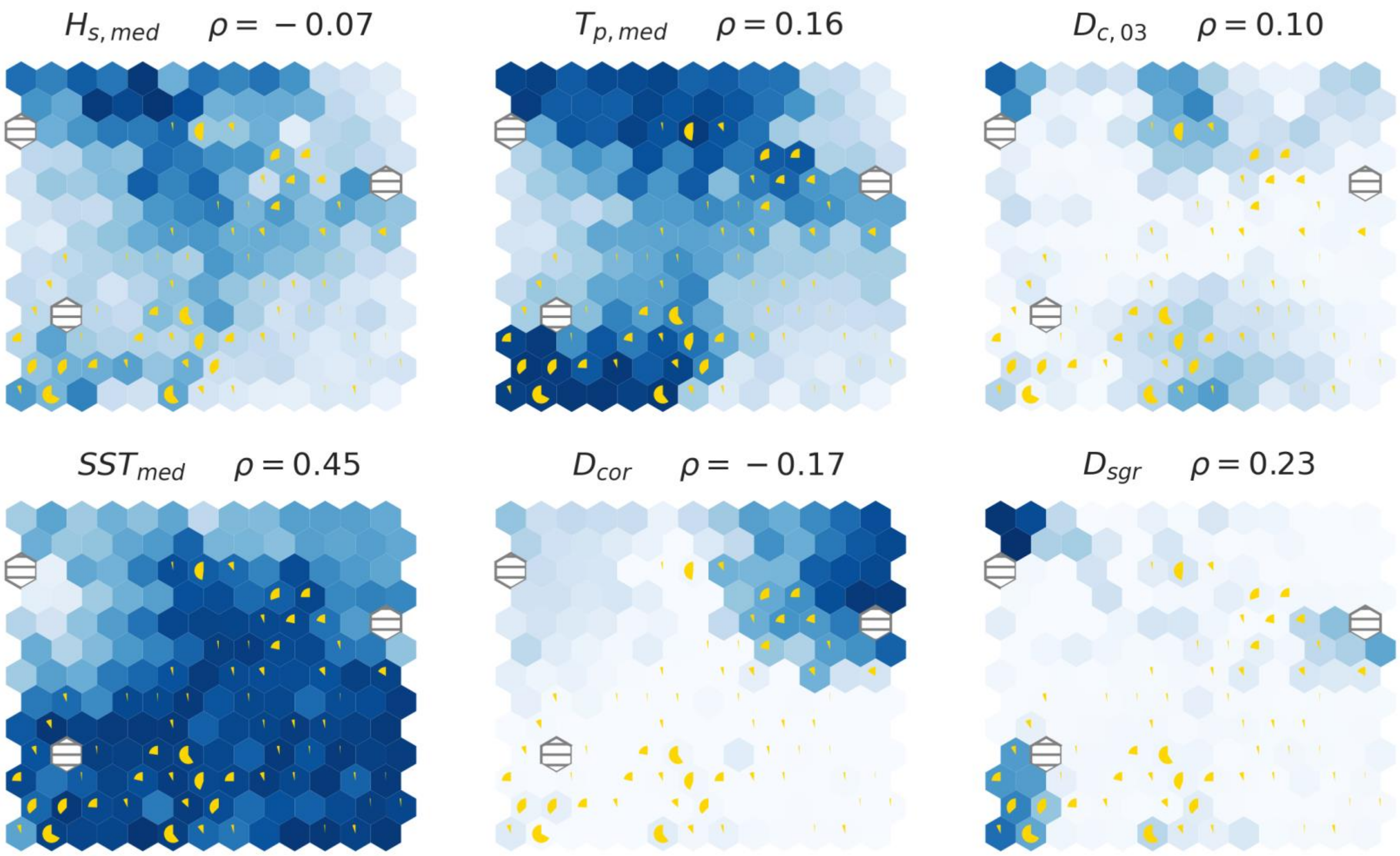


*Figure S8: SOM cluster lattices for the six indicators selected for olive ridley turtles (LO). The blue color scale indicates the cluster medians for the corresponding indicator (relative value, dark is highest, light is lowest). The yellow pie charts show the proportion of nesting Coastgons in each cluster. The title of each subplot shows the indicator and the Spearman correlation (ρ) between the cluster medians and the nesting percentages. Due to the SOM algorithm, some computed clusters may not represent any actual Coastgons (indicated by horizontally striped hexagons).*

## S6. Global and regional nesting suitability maps

This section contains all global and regional suitability maps. The maps are created for each species/region pair listed in Table 3 in the main paper, except the ones where no percentages are given (regions outside the RMUs for the species, according to Wallace et al.[10]). The regions are (see geographical overview in Fig. 4 of the main paper):

- Global (all Coastgons)
- Central East Pacific
- North West Atlantic
- South West Atlantic
- Central East Atlantic
- Mediterranean
- North West Indian
- South West Indian
- North East Indian

All figures consist of two parts, the suitability map (upper panel) and the corresponding box-plots (lower panel). The suitability maps show the World's coastline for the given region, overlayed by the Coastgons, colored according to their category:

- Unsuitable (U) Grey
- Potentially suitable (S) Light blue
- Observed nesting (O) Species color (CC: orange, CM: green, EI: red, DC: purple, LO: yellow)

Below each suitability map are six box-plots, showing the distribution of the six selected coastal indicators of each species over the three Coastgon categories. Only the Coastgons within the corresponding region are included in the box-plots (category sizes are given as well). Finally, below each box-plot are the p-values corresponding to the two-sample KS tests between each category, denoted by their subscripts (e.g., $p_{O|S}$ is the p-value for the KS test between the observed nesting and potentially suitable Coastgons). For a more detailed explanation please see the methods section of the main paper

S6.1 Global (all species)

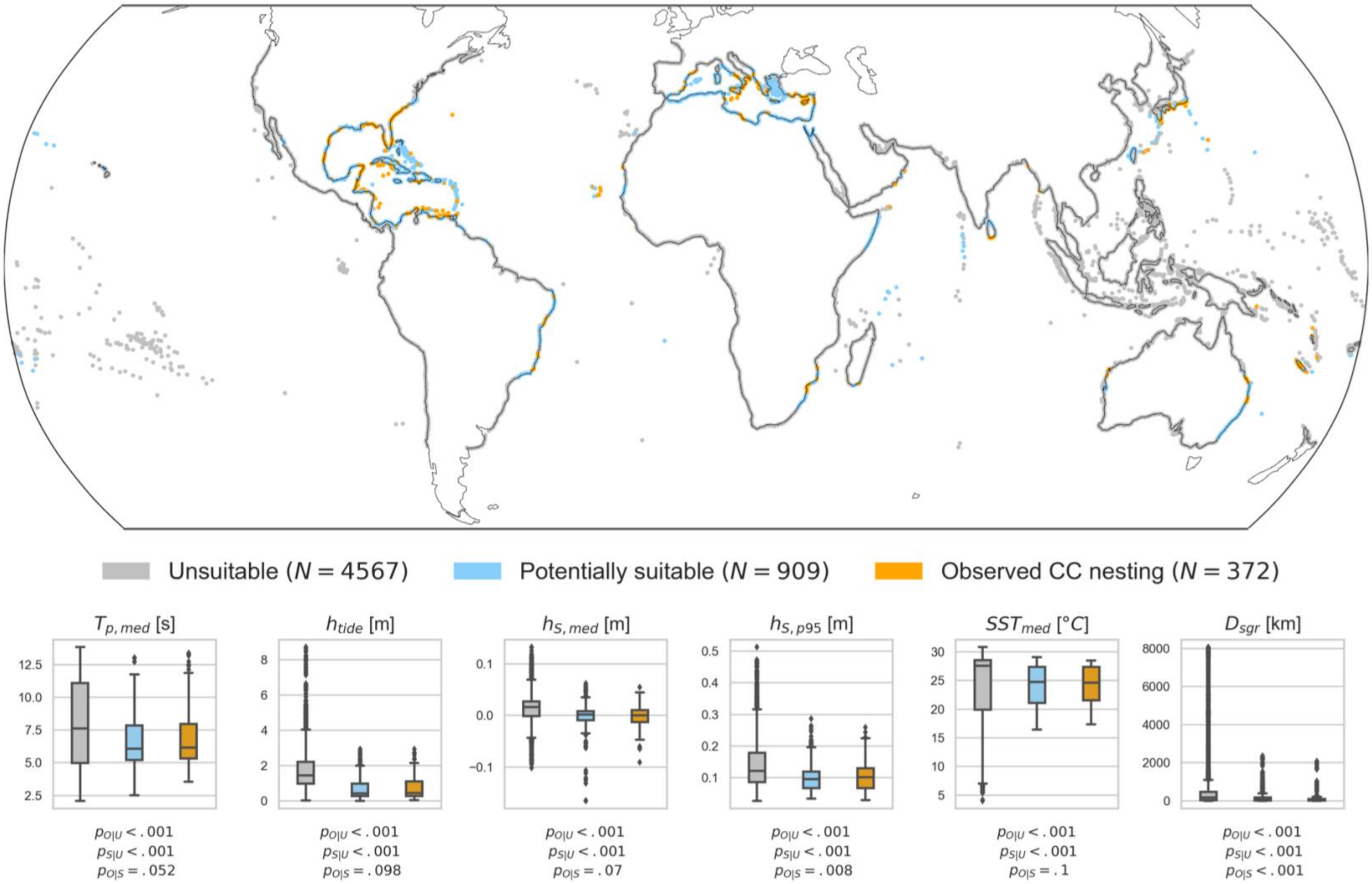


*Figure S9: Global nesting suitability map for loggerhead turtles (CC)*

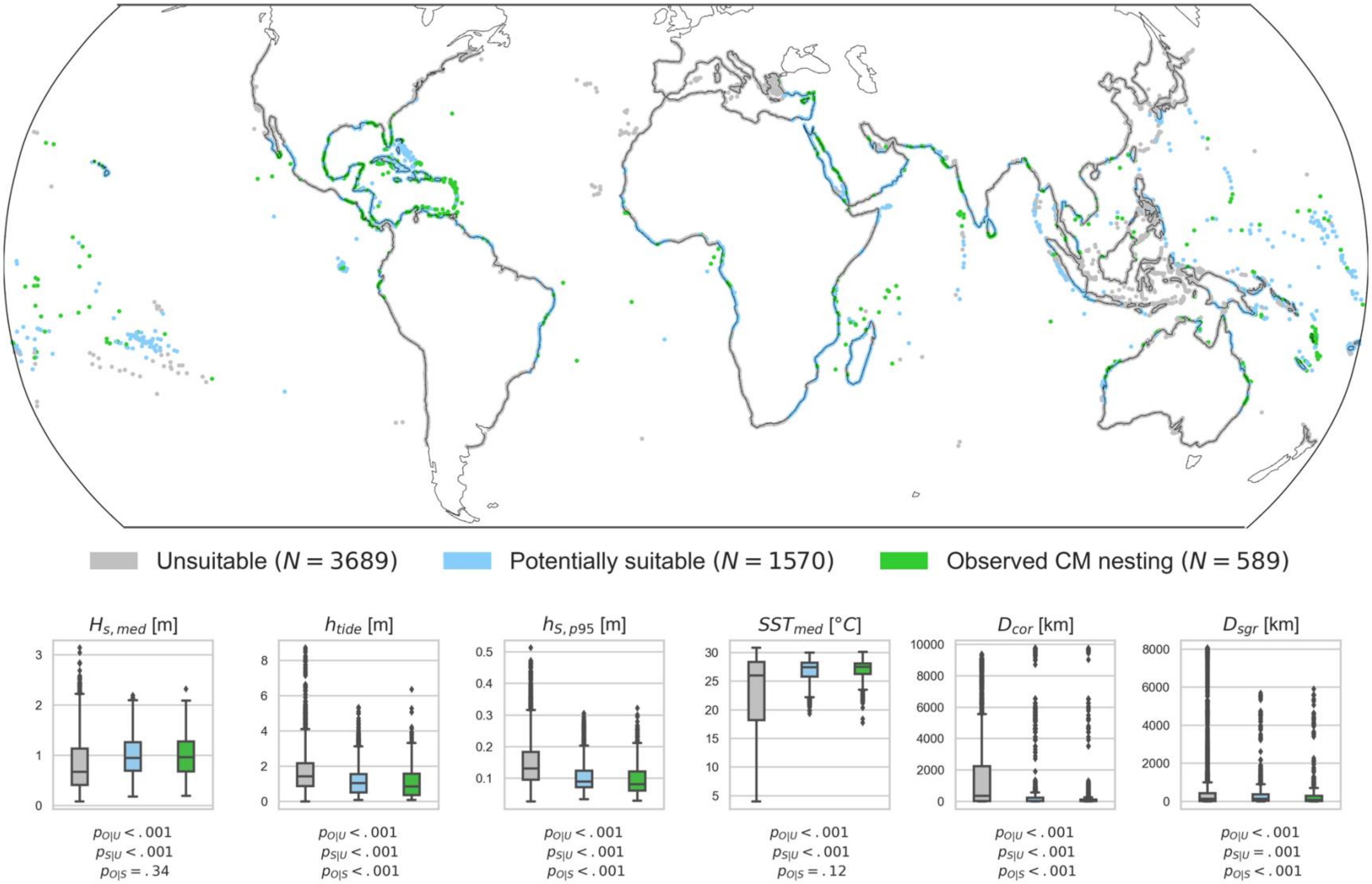


*Figure S10: Global nesting suitability map for green turtles (CM)*

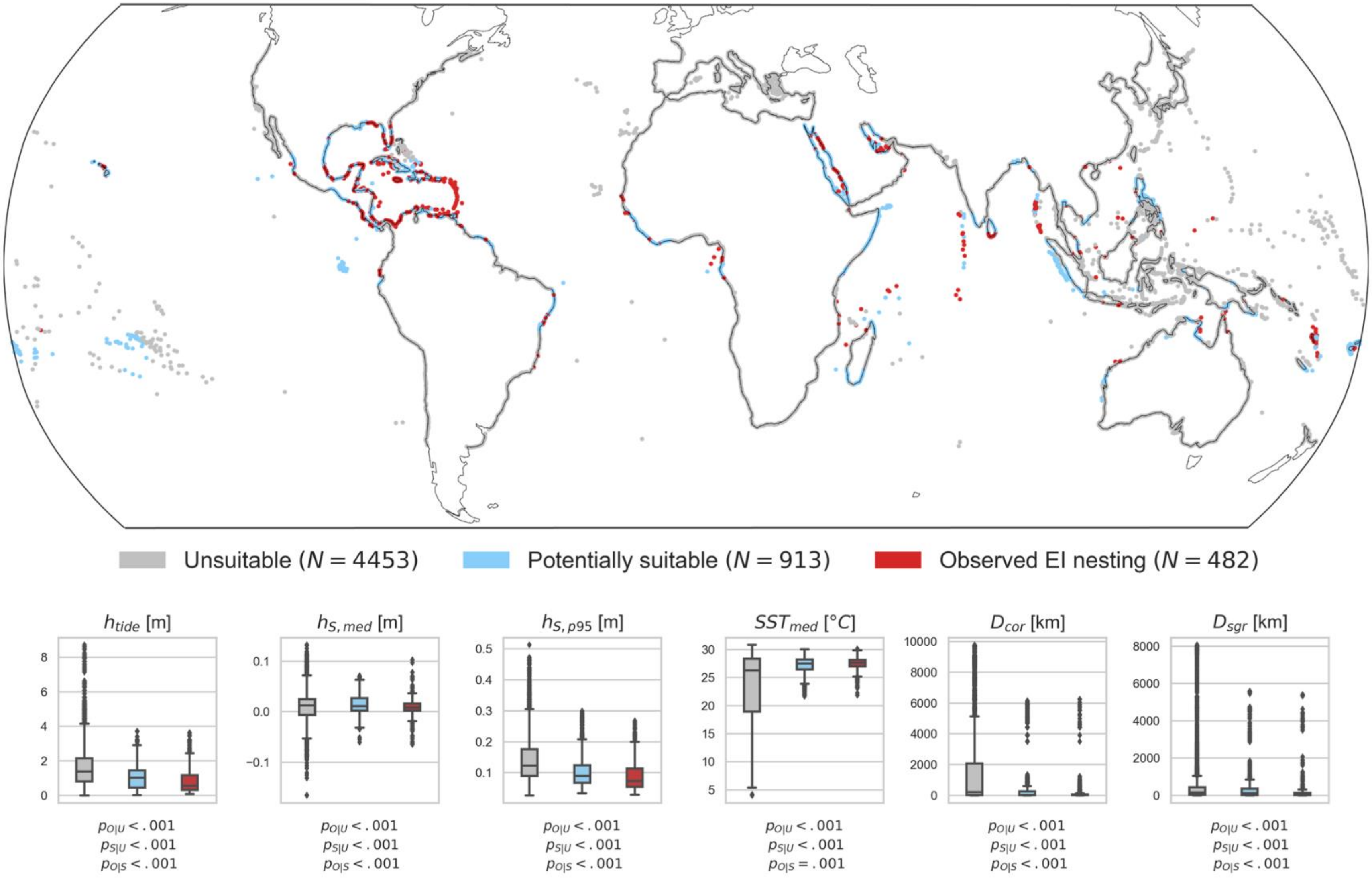


*Figure S11: Global nesting suitability map for hawksbill turtles (EI)*

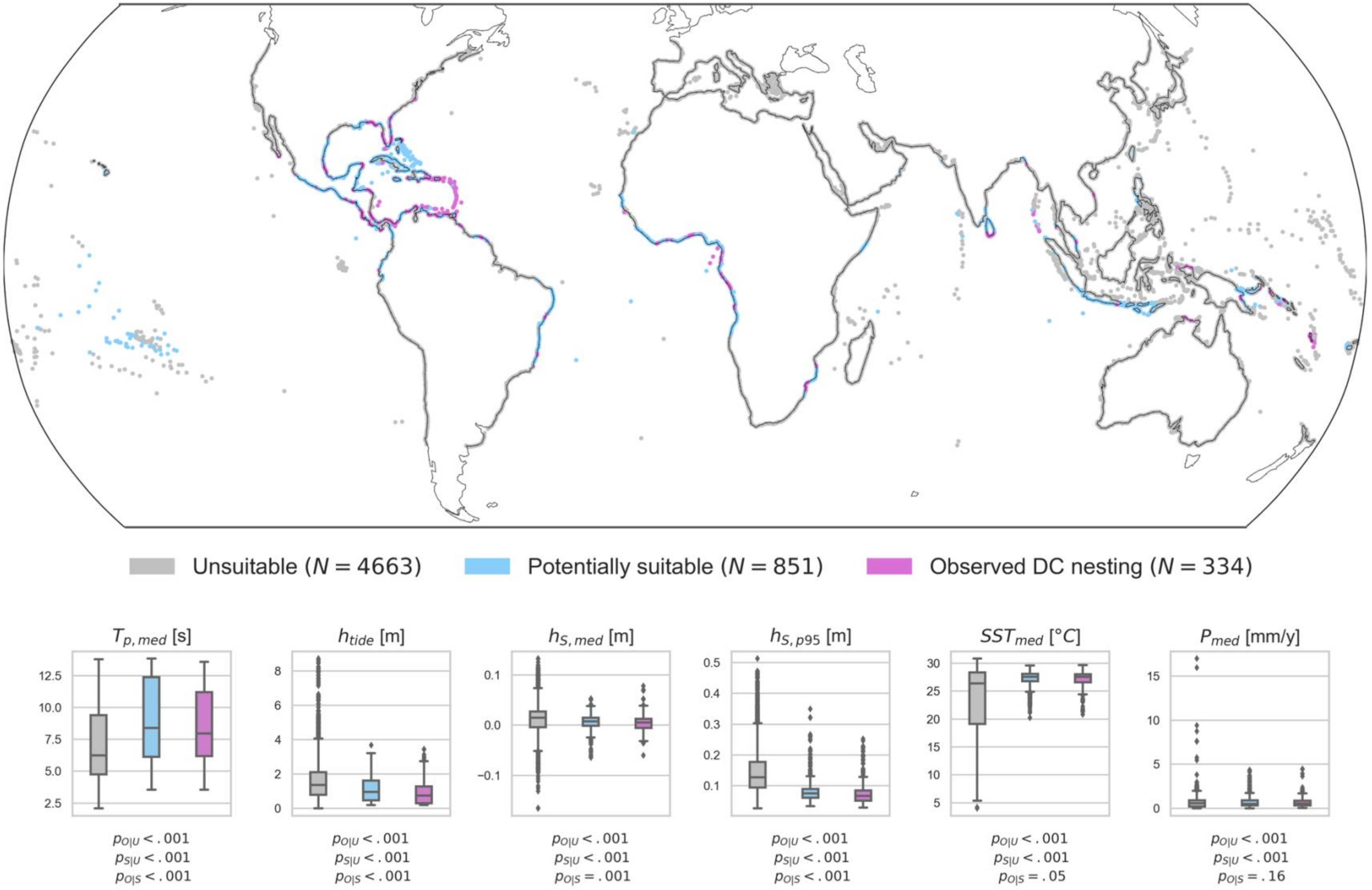


*Figure S12: Global nesting suitability map for leatherback turtles (DC)*

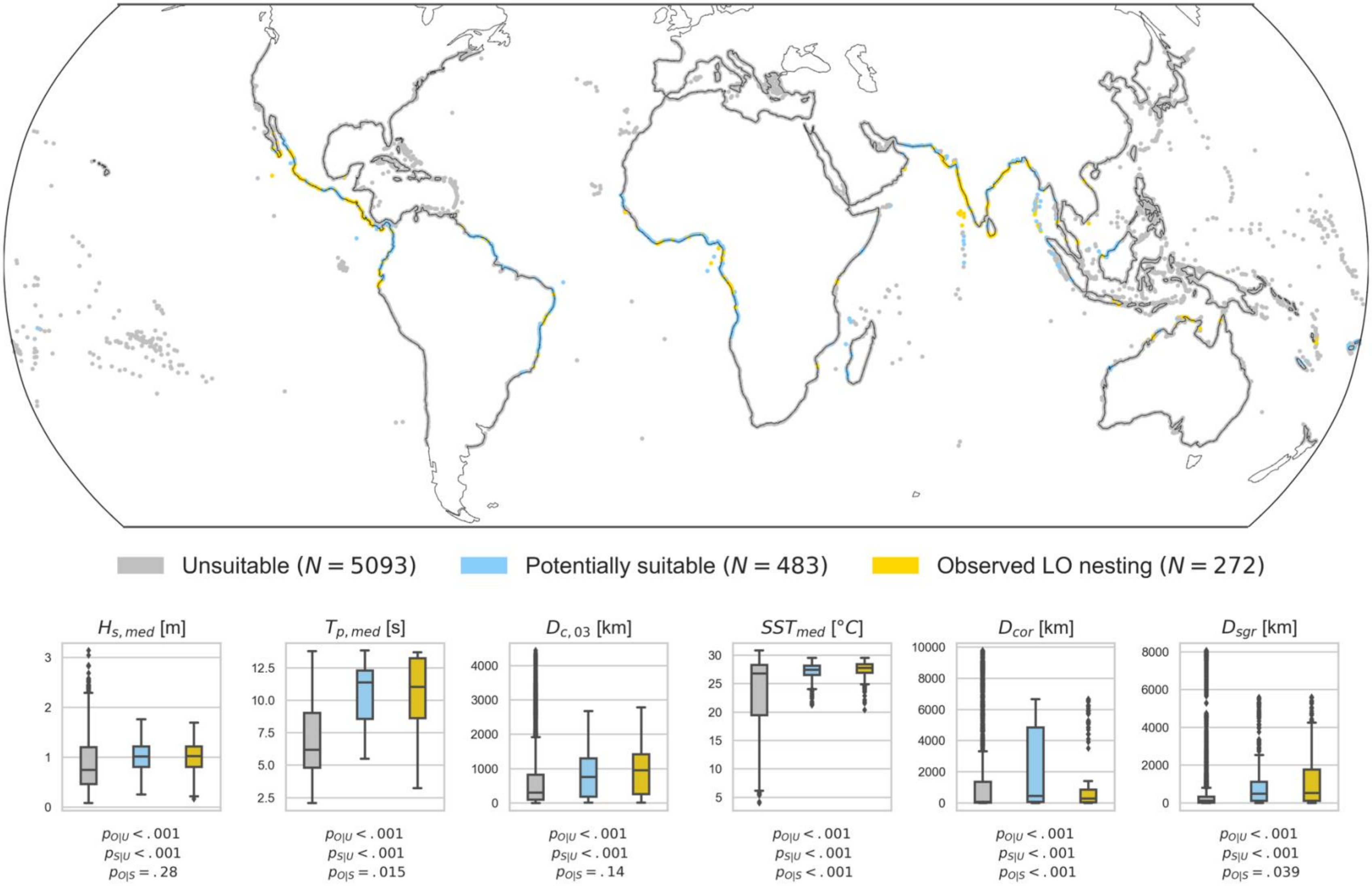


*Figure S13: Global nesting suitability map for olive ridley turtles (LO)*

## S6.2 Central East Pacific (CM, EI, DC, LO)

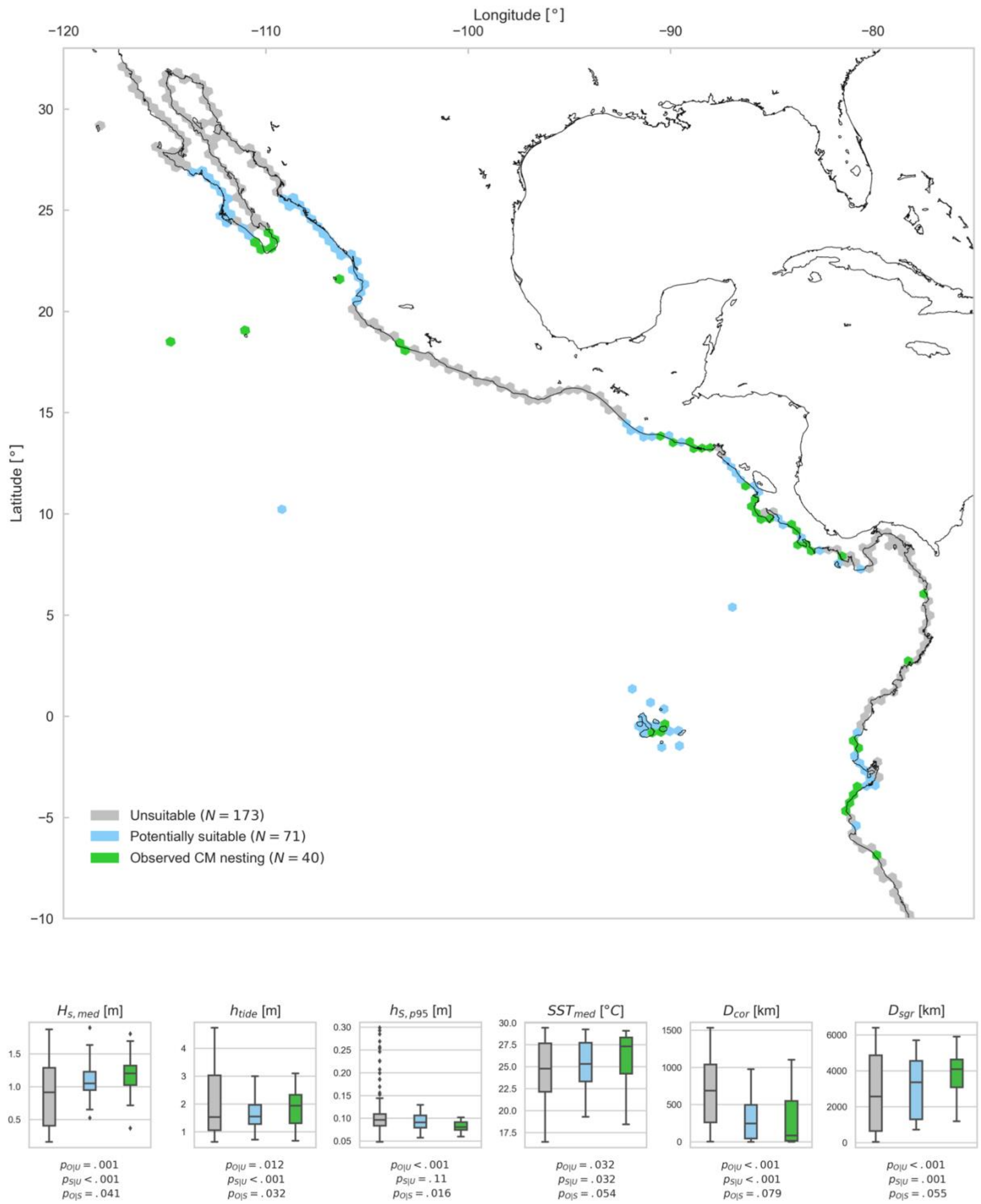


*Figure S14: Nesting suitability map for green turtles (CM) in the Central East Pacific*

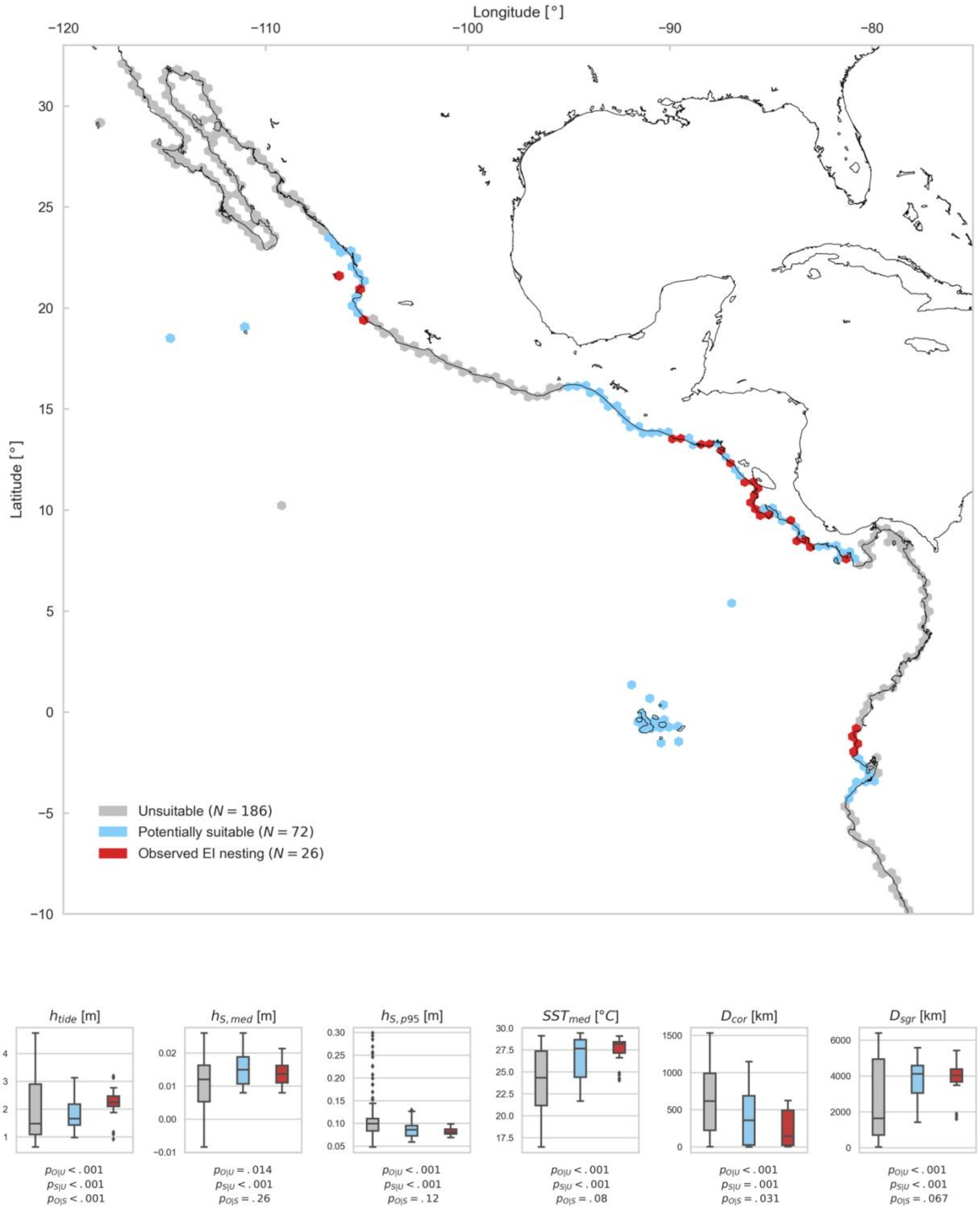


*Figure S15: Nesting suitability map for hawksbill turtles (EI) in the Central East Pacific*

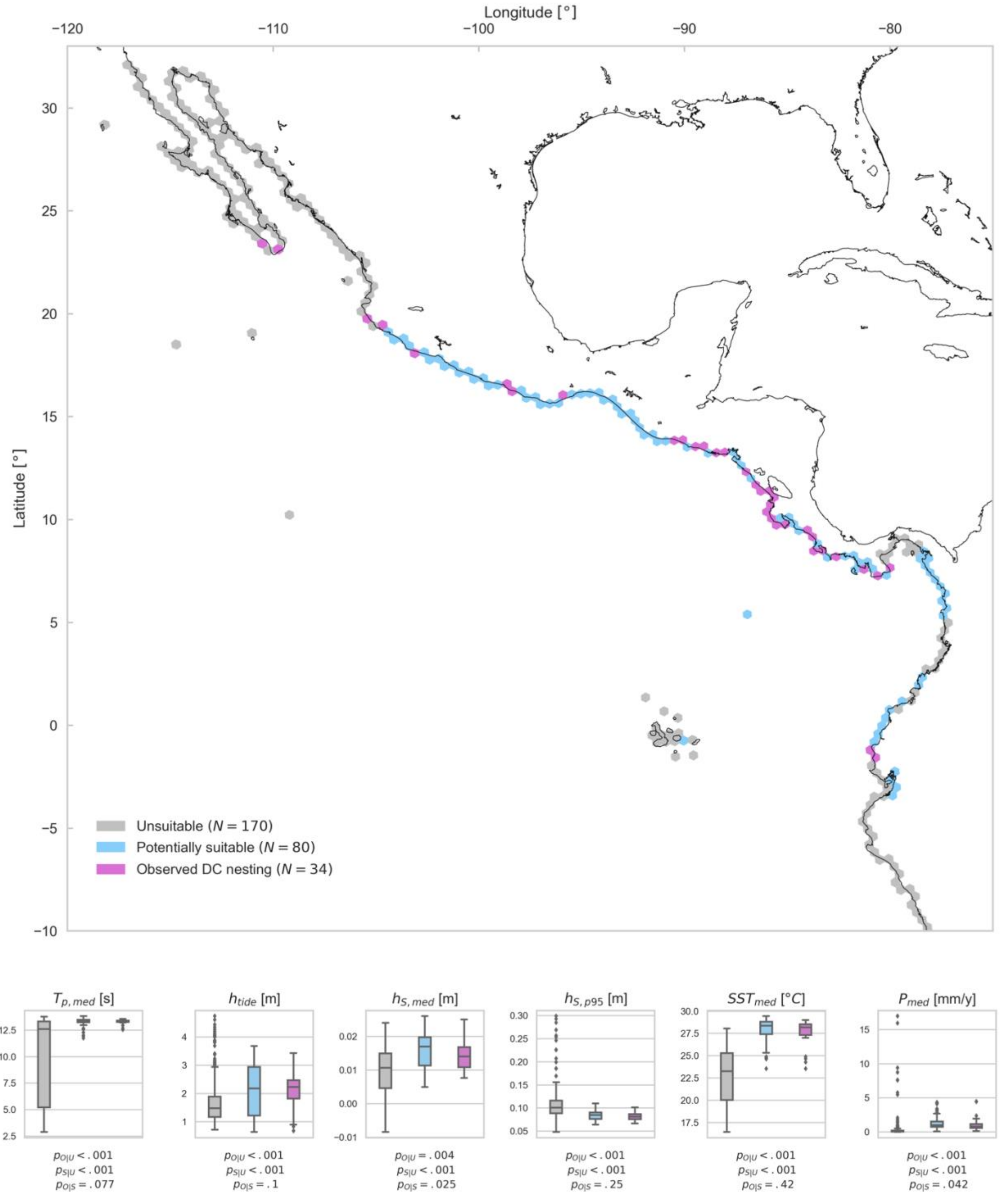


*Figure S16: Nesting suitability map for leatherback turtles (DC) in the Central East Pacific*

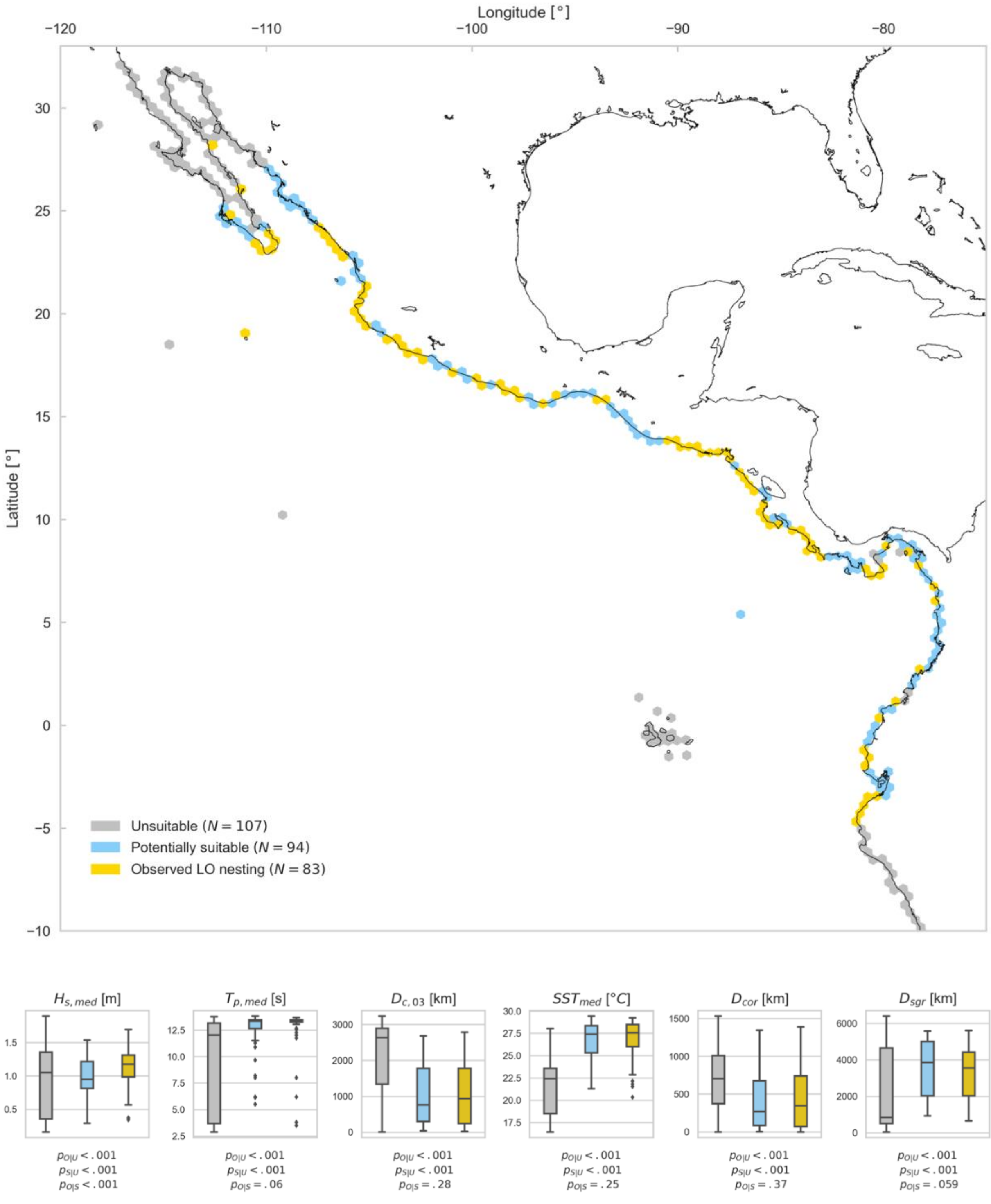


*Figure S17: Nesting suitability map for olive ridley turtles (LO) in the Central East Pacific*

## S6.3 North West Atlantic (CC, CM, EI, DC)

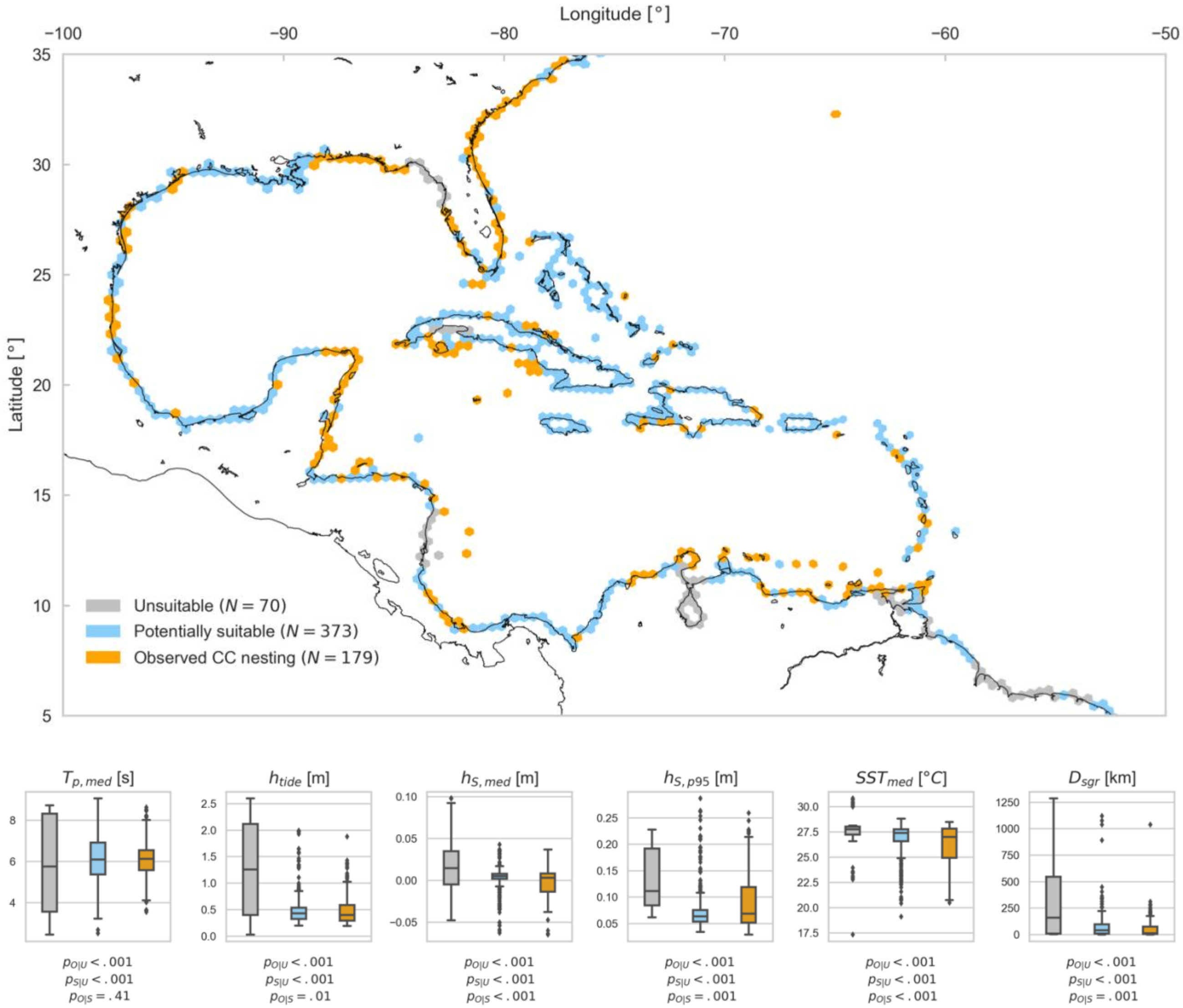


*Figure S18: Nesting suitability map for loggerhead turtles (CC) in the North West Atlantic*

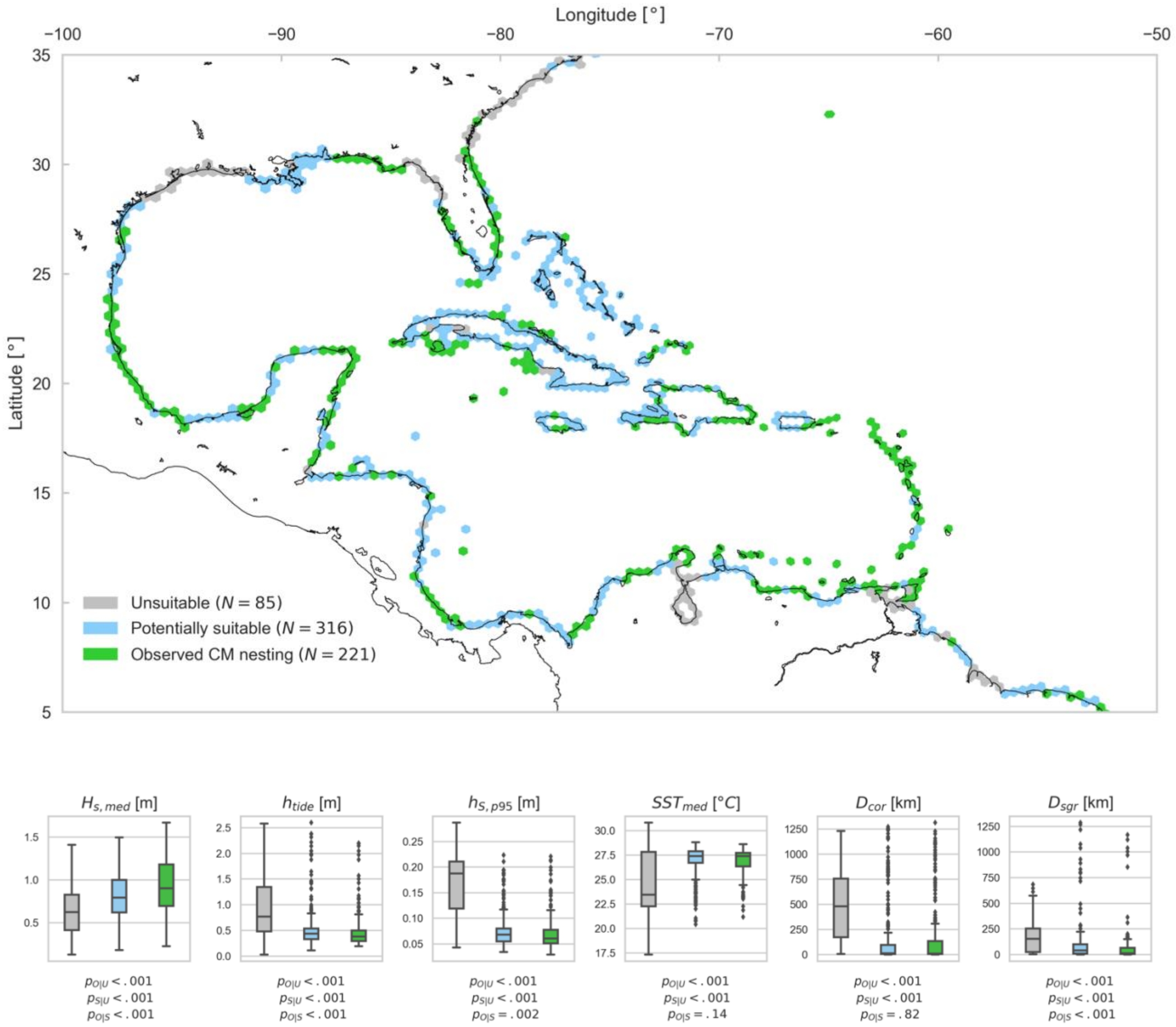


*Figure S19: Nesting suitability map for green turtles (CM) in the North West Atlantic*

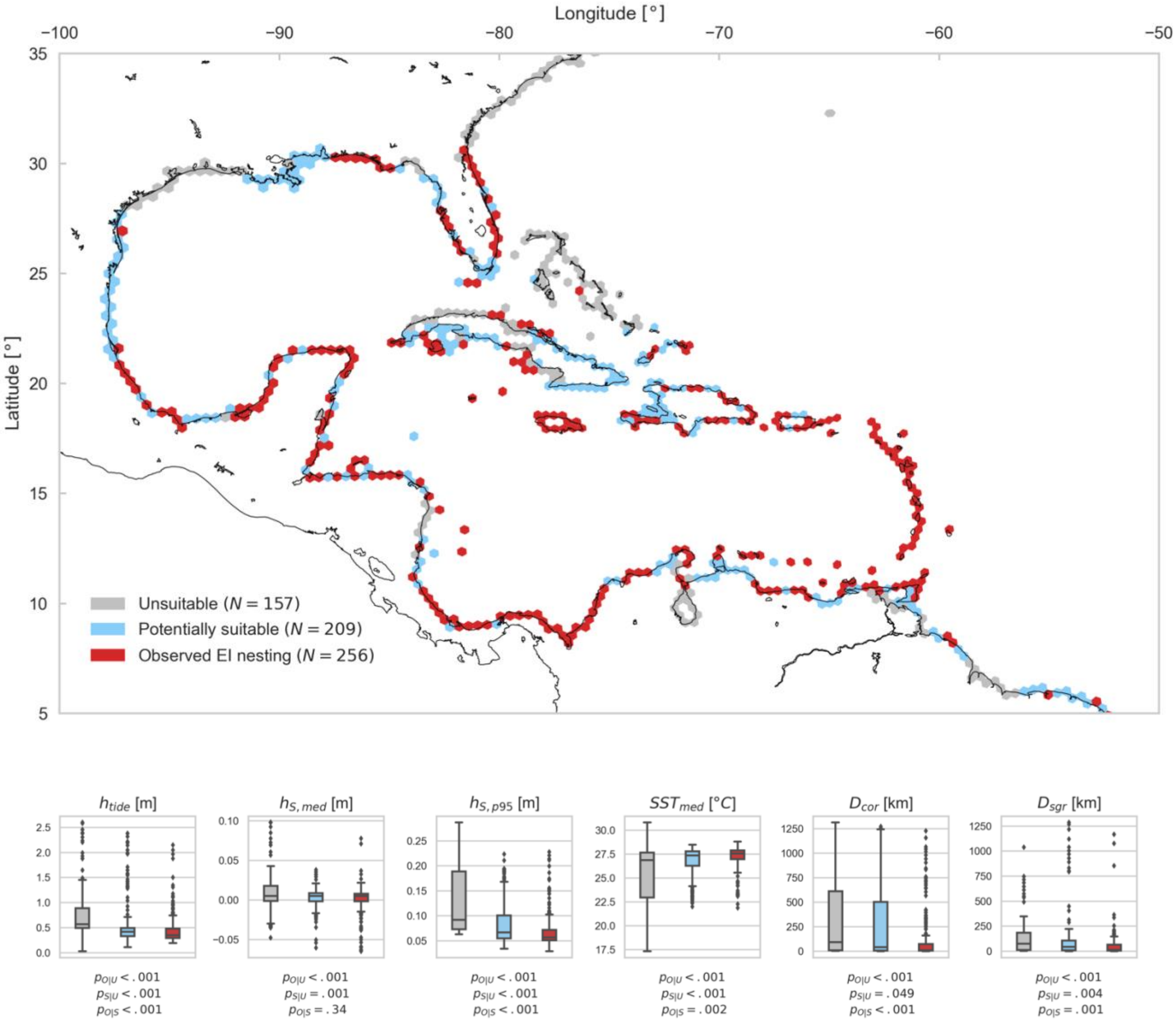


*Figure S20: Nesting suitability map for hawksbill turtles (EI) in the North West Atlantic*

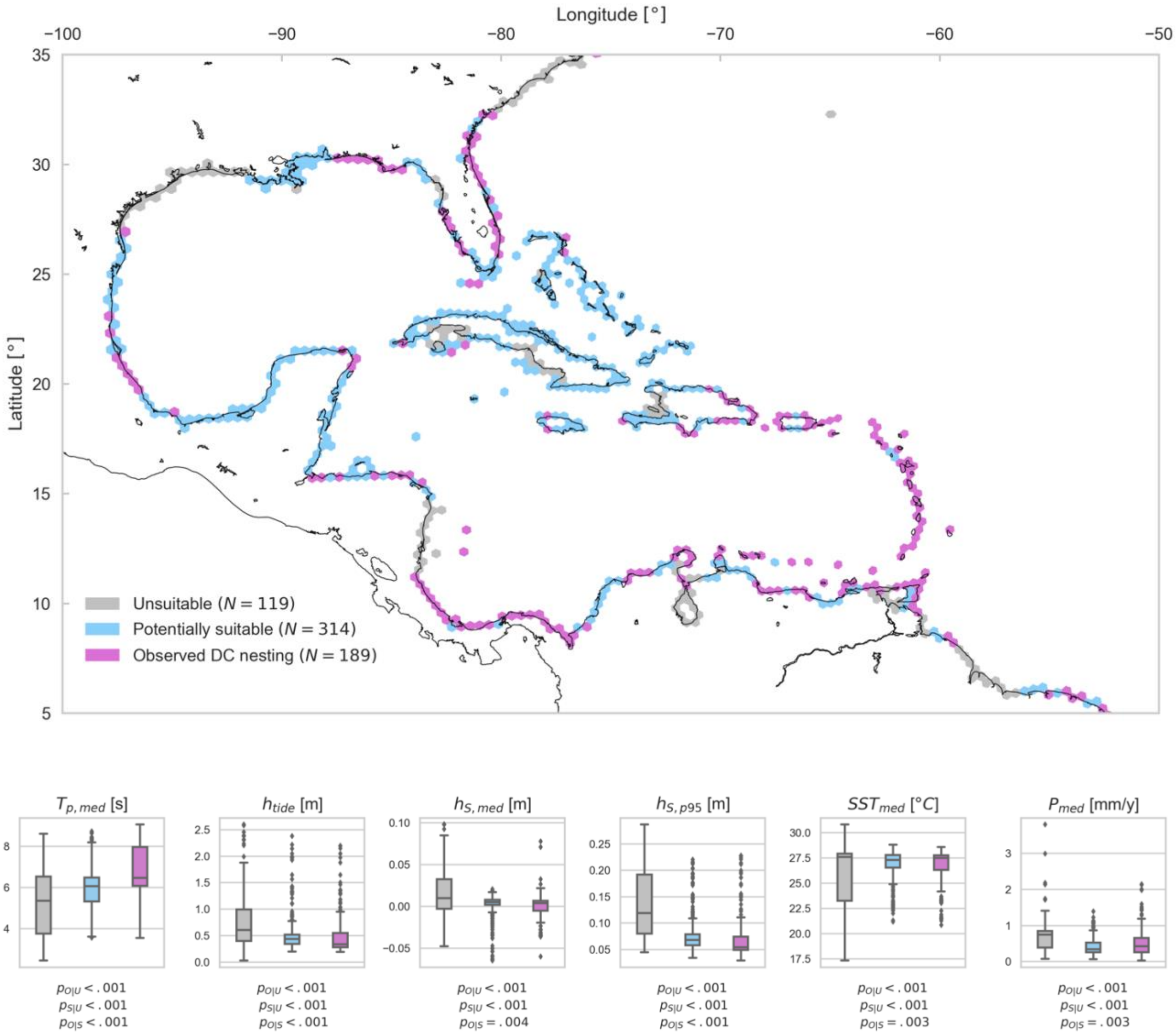


*Figure S21: Nesting suitability map for leatherback turtles (DC) in the North West Atlantic*

## S6.4 South West Atlantic (all species)

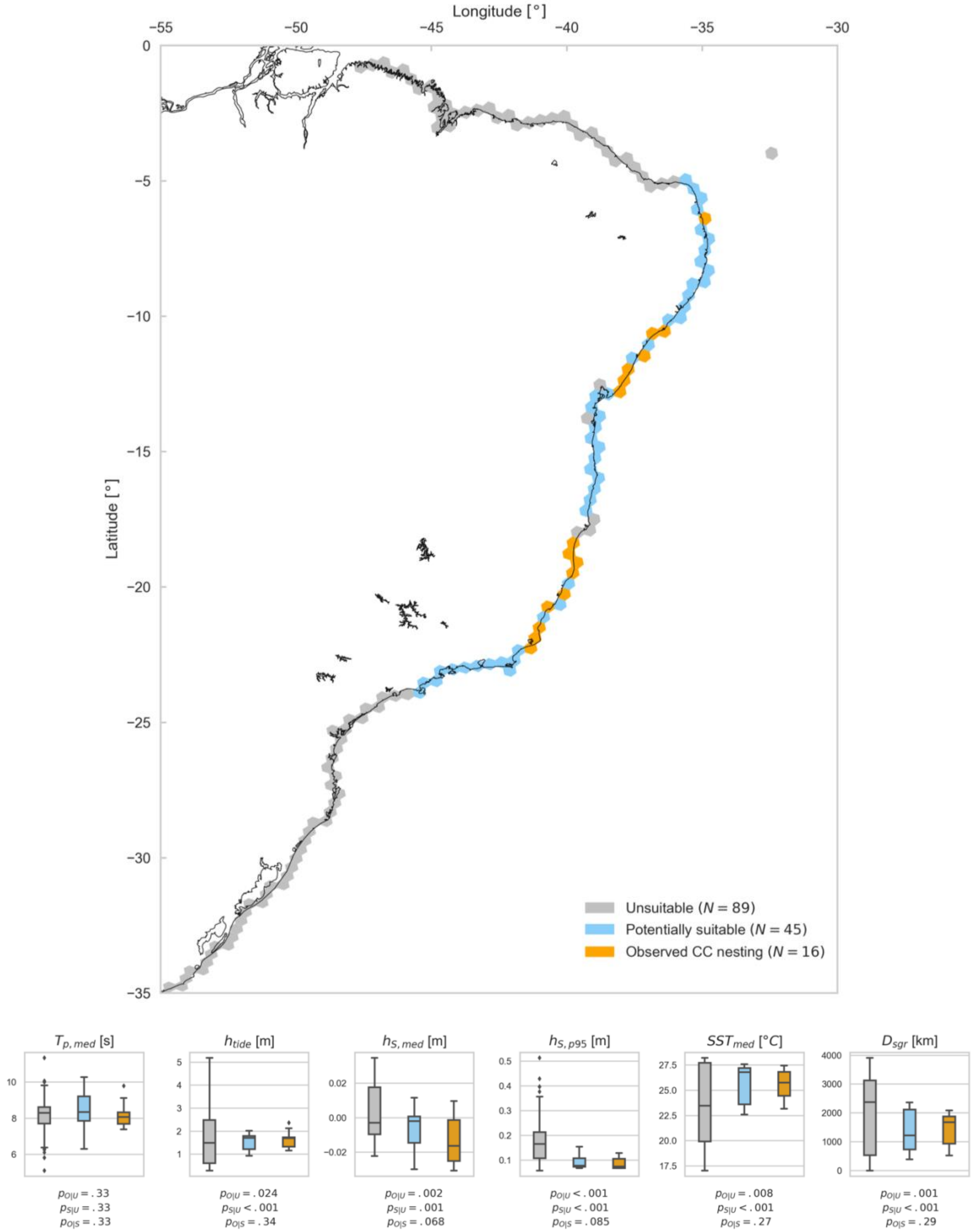


*Figure S22: Nesting suitability map for loggerhead turtles (CC) in the South West Atlantic*

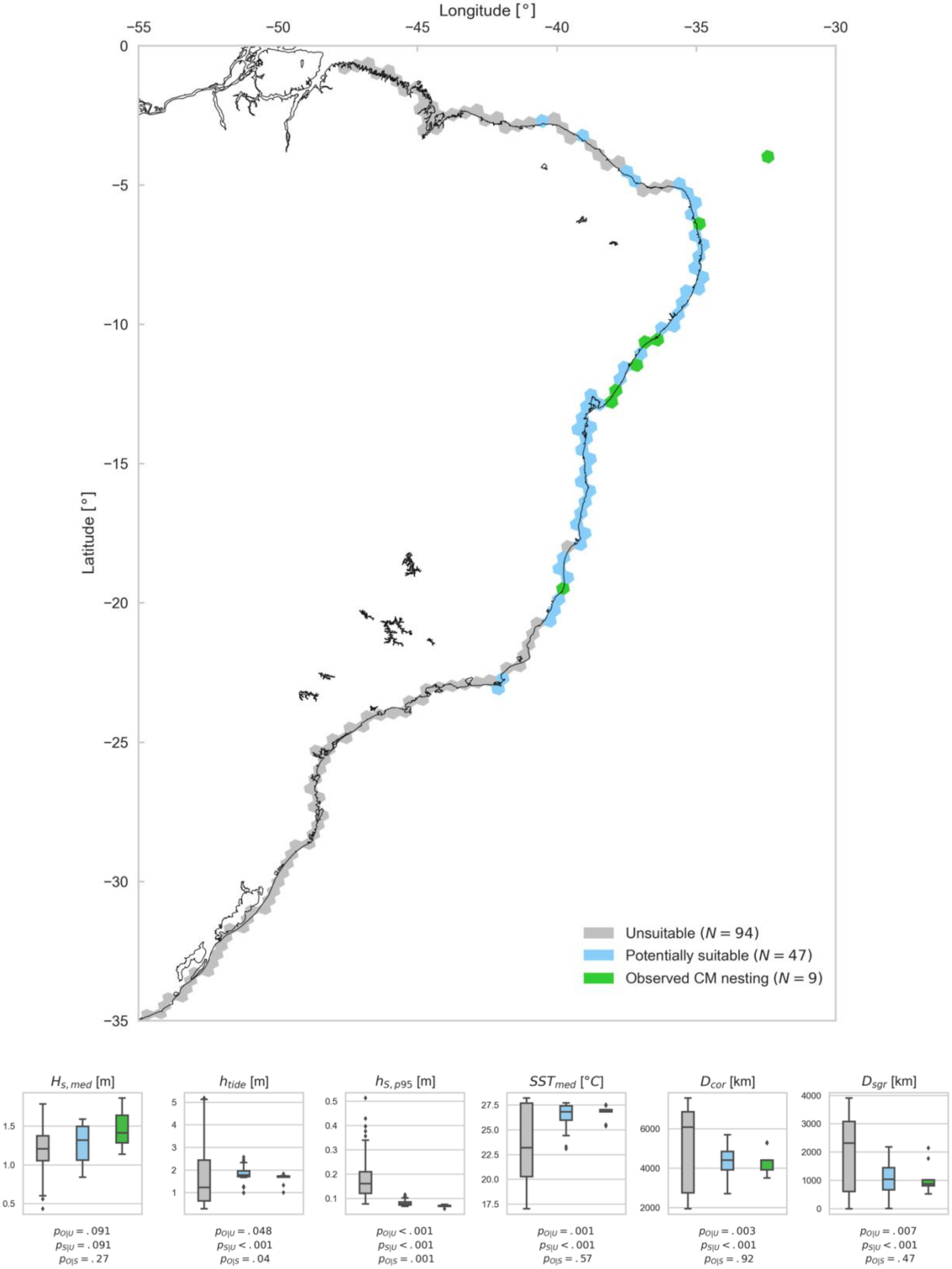


*Figure S23: Nesting suitability map for green turtles (CM) in the South West Atlantic*

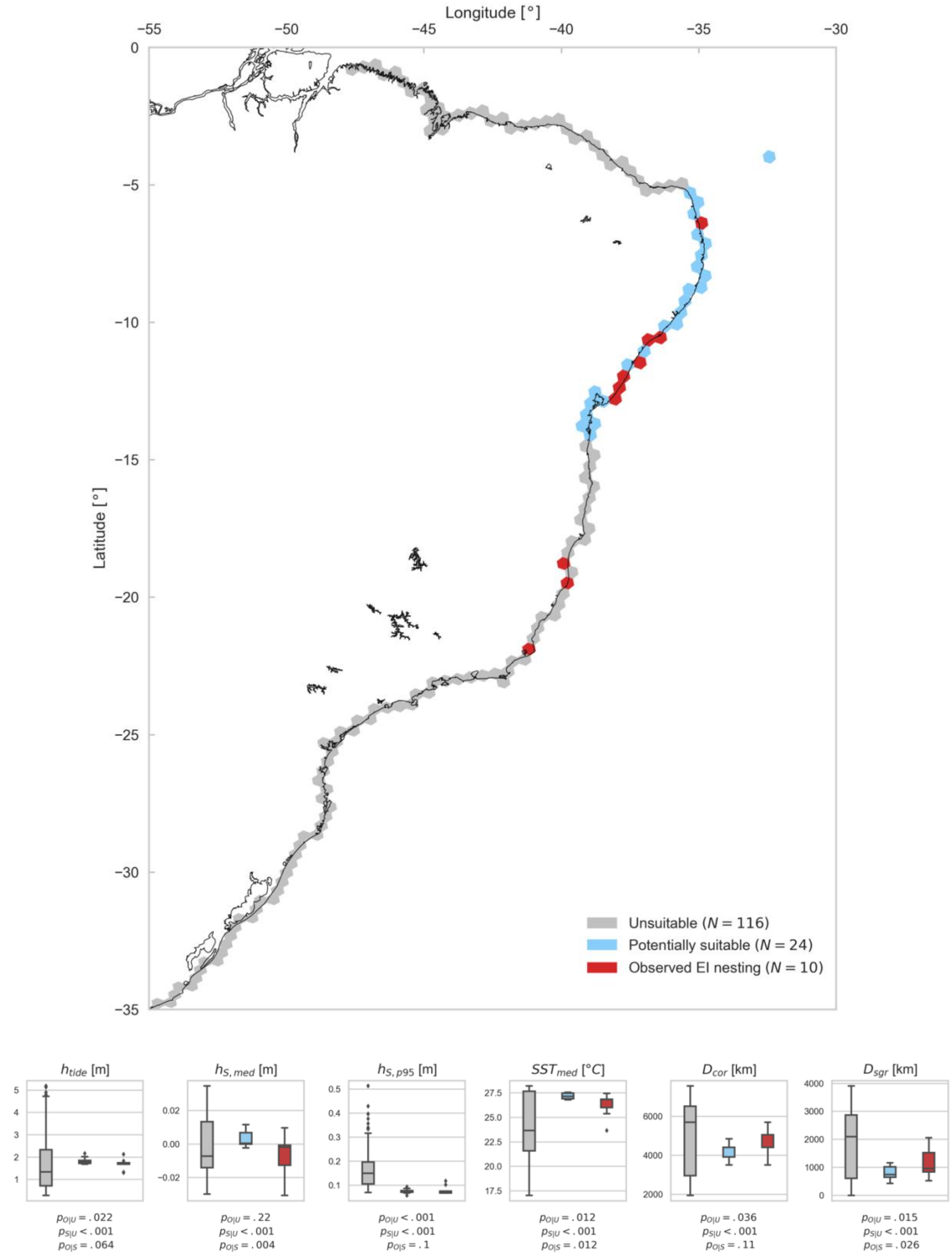


*Figure S24: Nesting suitability map for hawksbill turtles (EI) in the South West Atlantic*

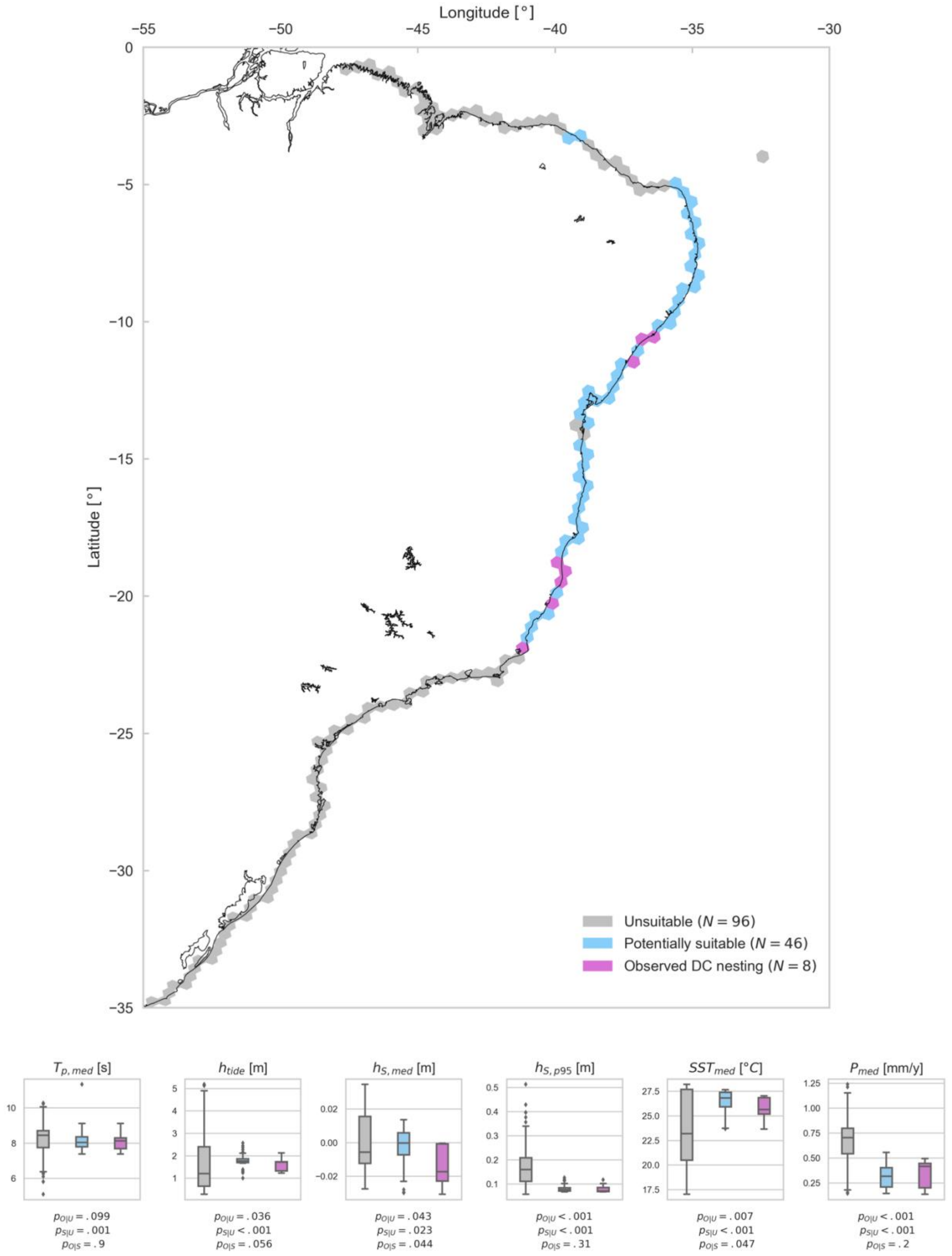


*Figure S25: Nesting suitability map for leatherback turtles (DC) in the South West Atlantic*

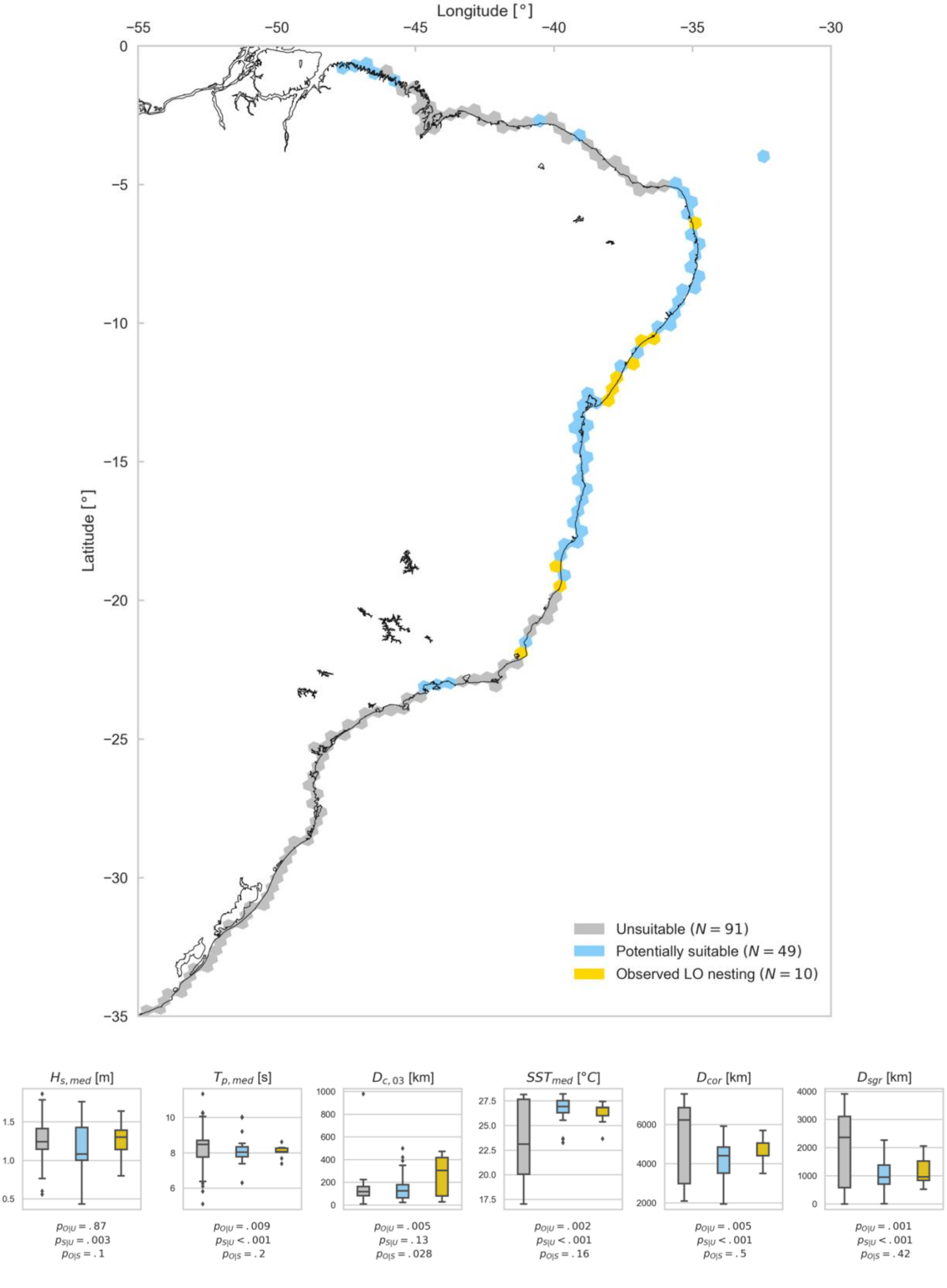


*Figure S26: Nesting suitability map for olive ridley turtles (LO) in the South West Atlantic*

S6.5 Central East Atlantic (CM, EI, DC, LO)

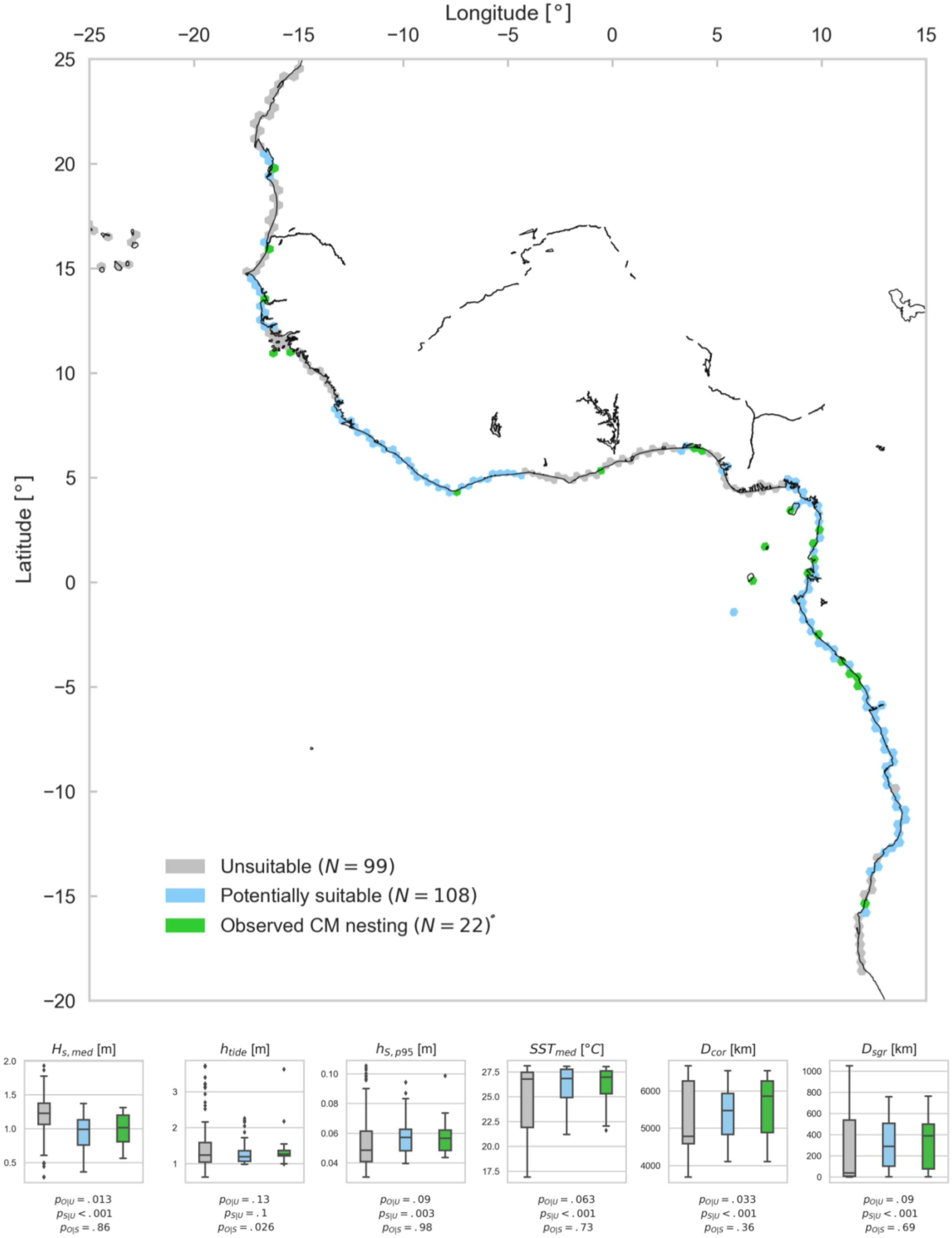


*Figure S27: Nesting suitability map for green turtles (CM) in the Central East Atlantic*

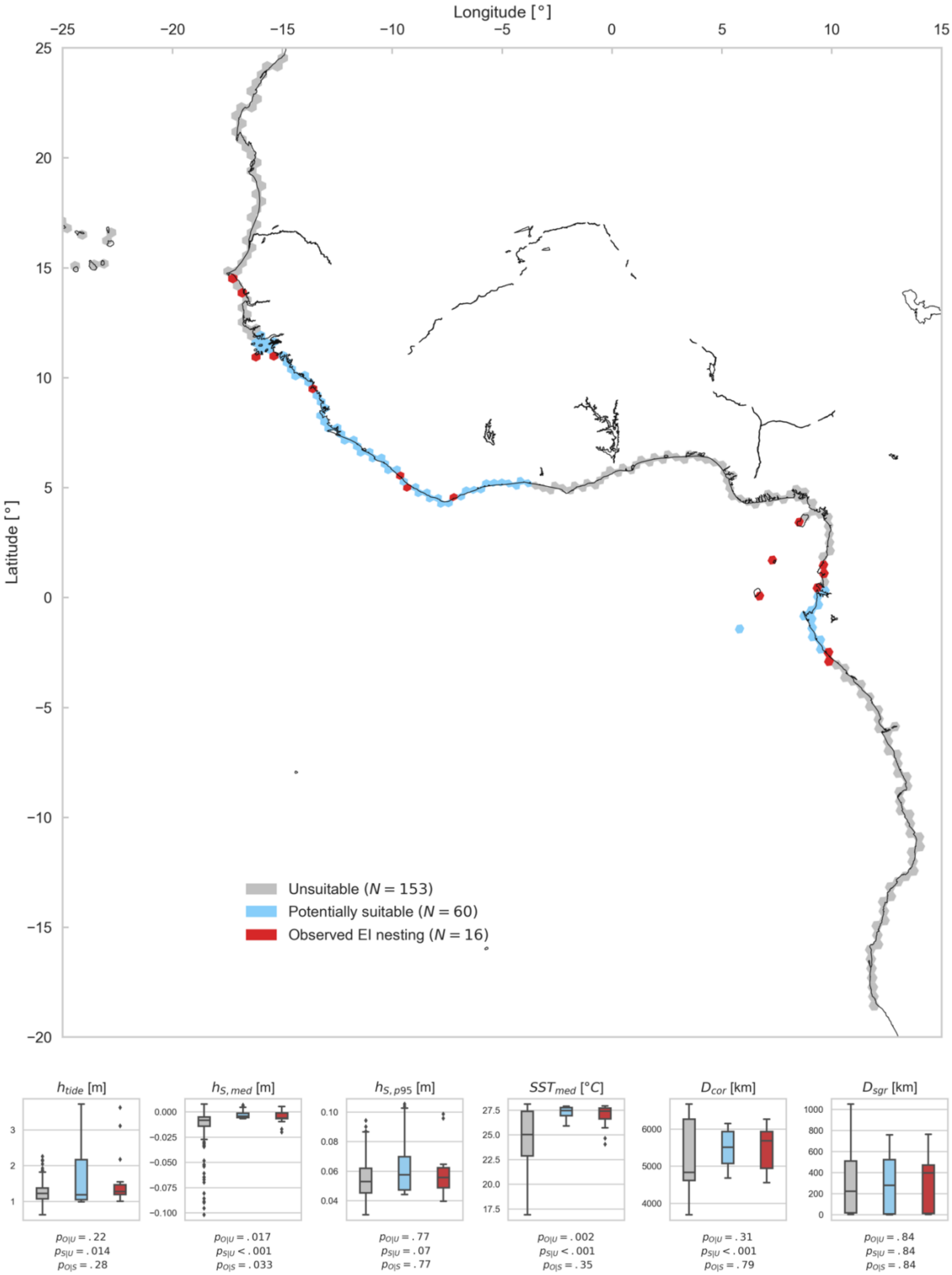


*Figure S28: Nesting suitability map for hawksbill turtles (EI) in the Central East Atlantic*

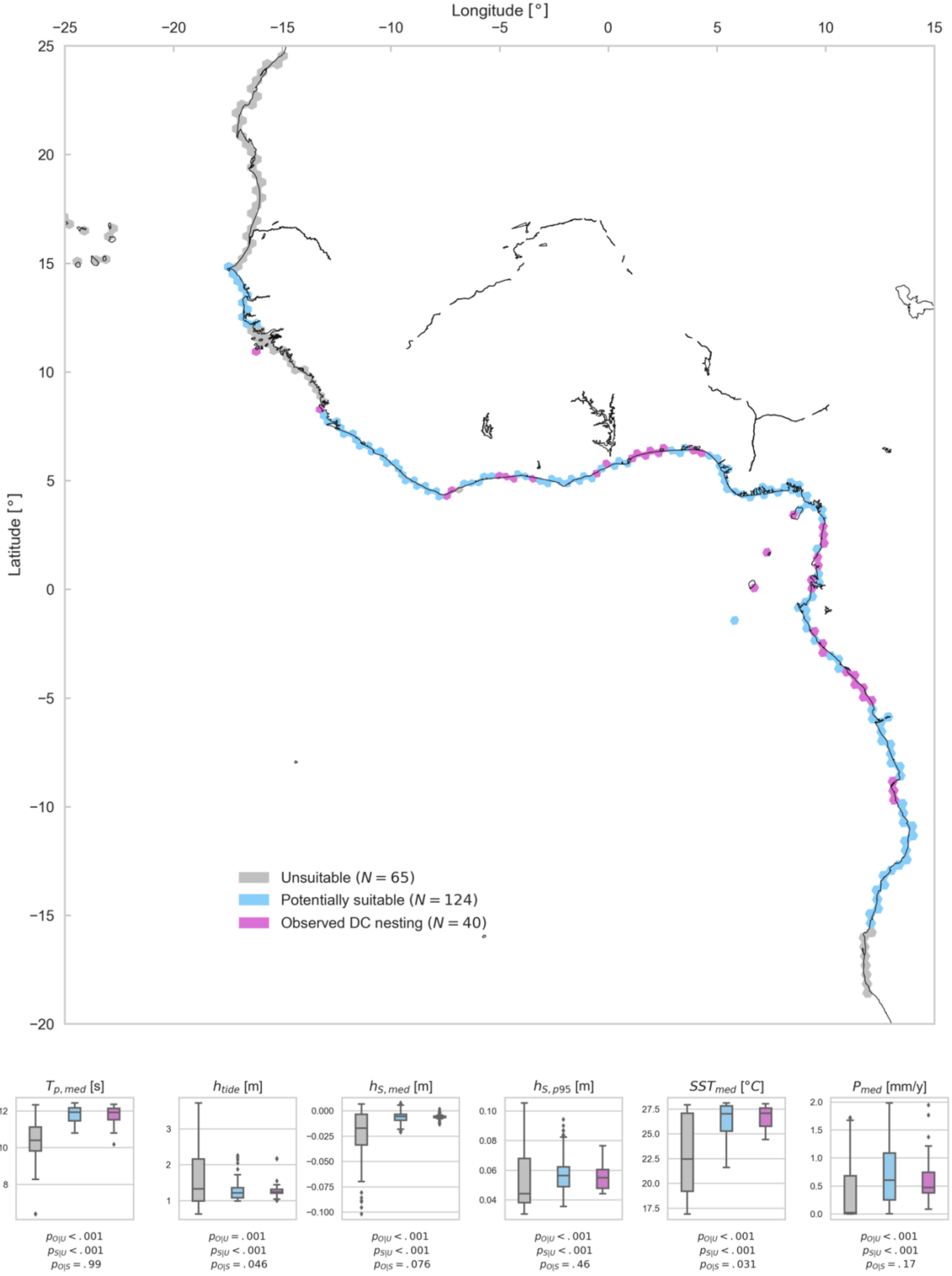


*Figure S29: Nesting suitability map for leatherback turtles (DC) in the Central East Atlantic*

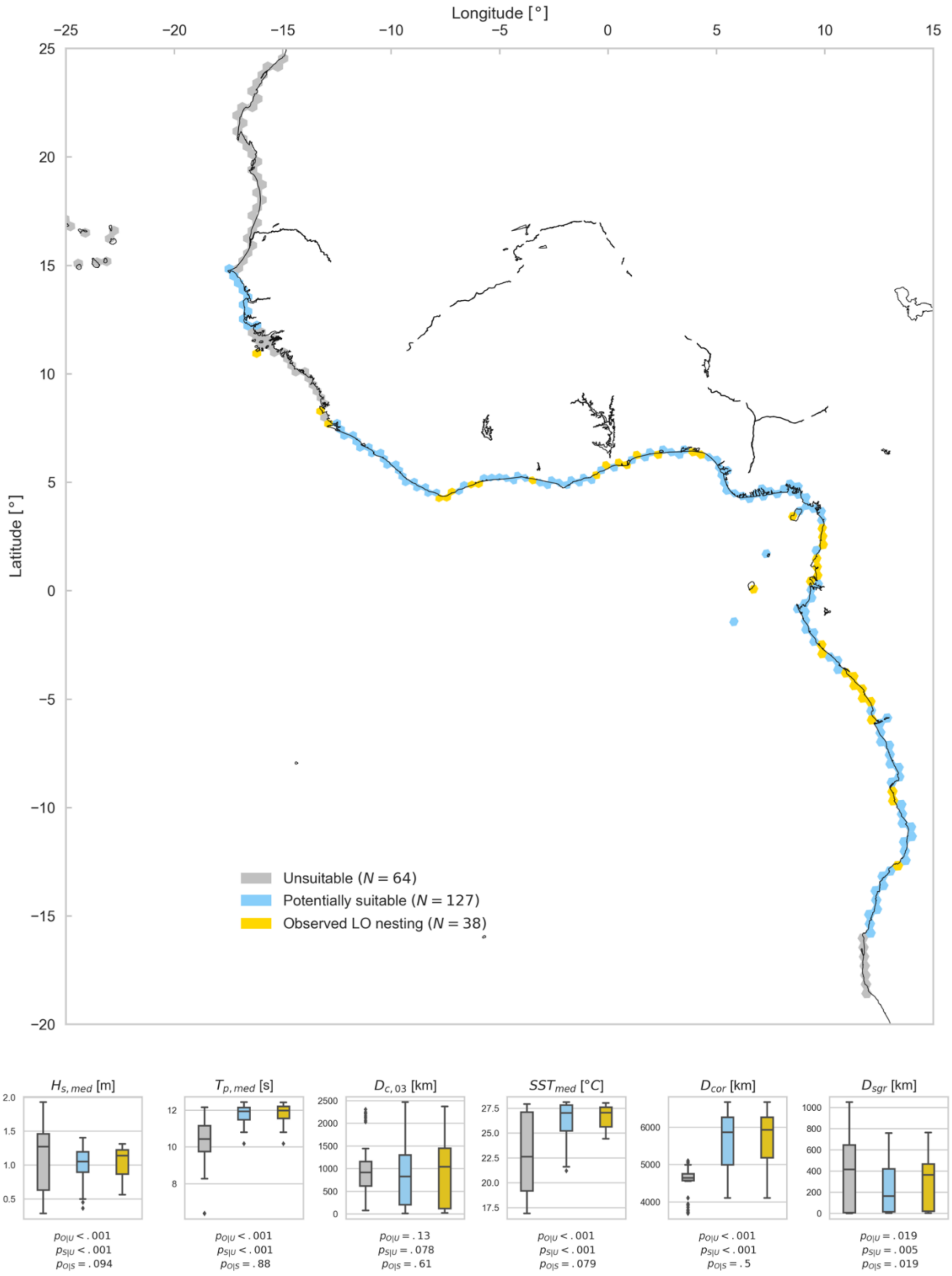


*Figure S30: Nesting suitability map for olive ridley turtles (LO) in the Central East Atlantic*

S6.6 Mediterranean (CC, CM)

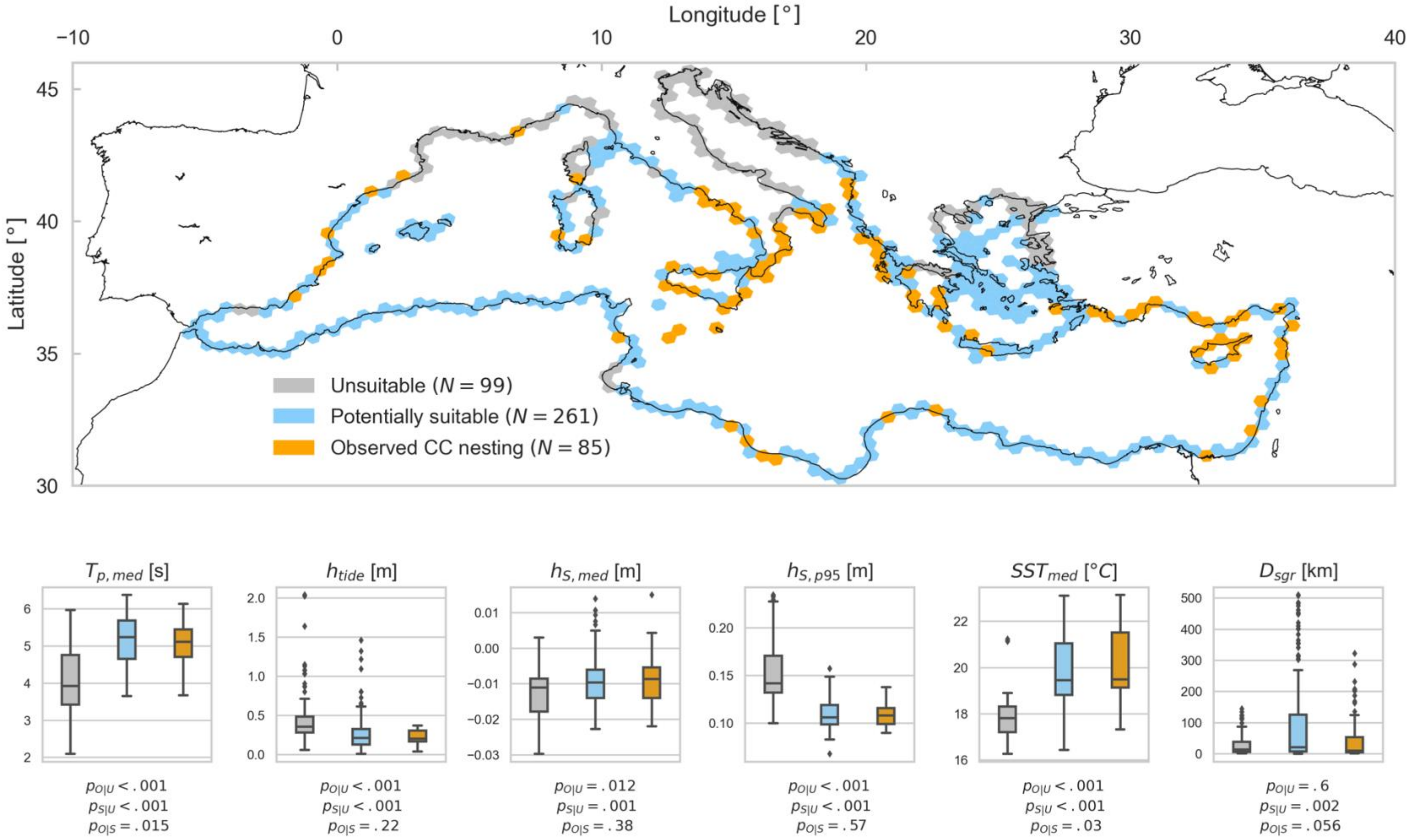


*Figure S31: Nesting suitability map for loggerhead turtles (CC) in the Mediterranean*

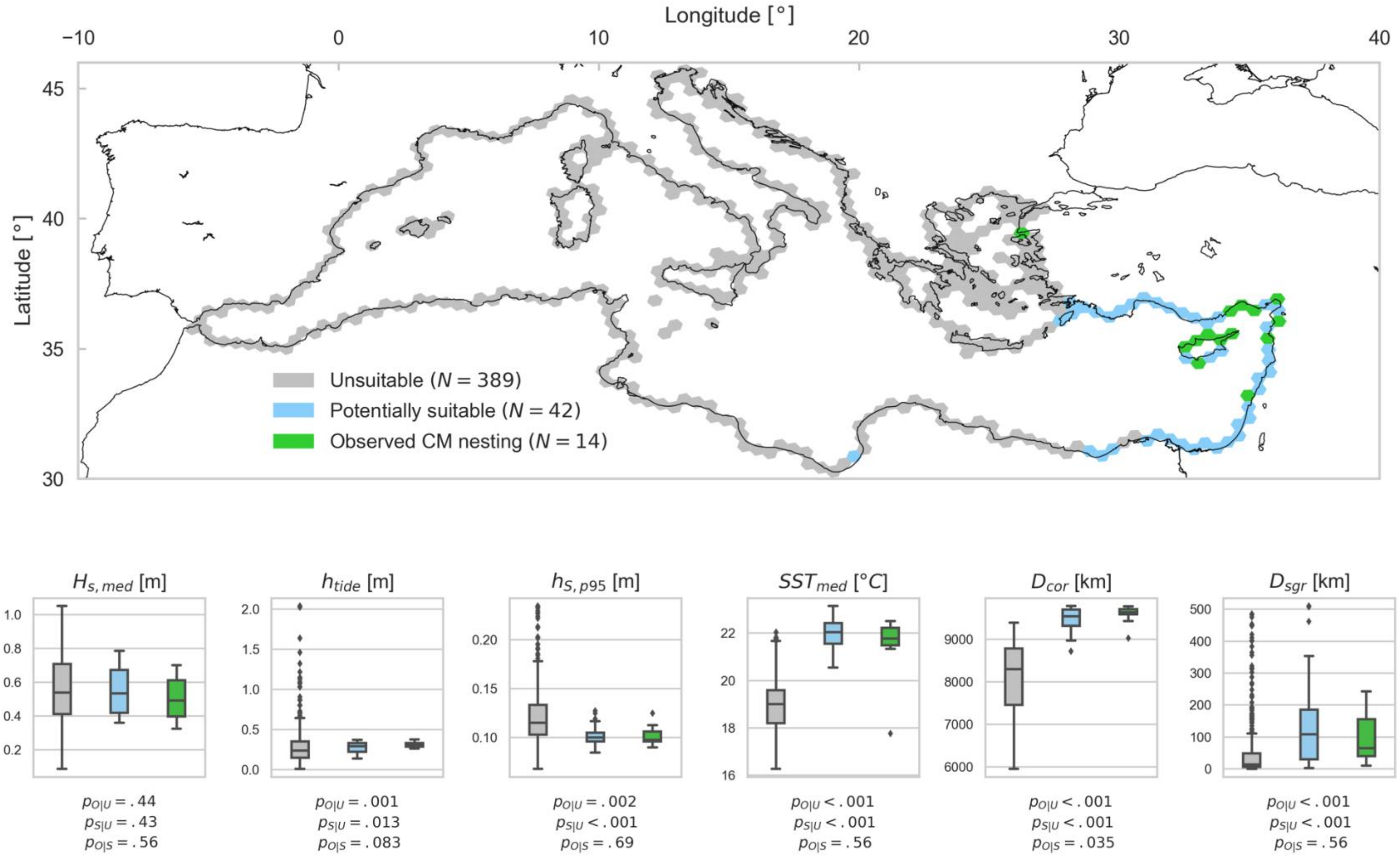


*Figure S32: Nesting suitability map for green turtles (CM) in the Mediterannean*

## S6.7 North West Indian (CC, CM, EI, LO)

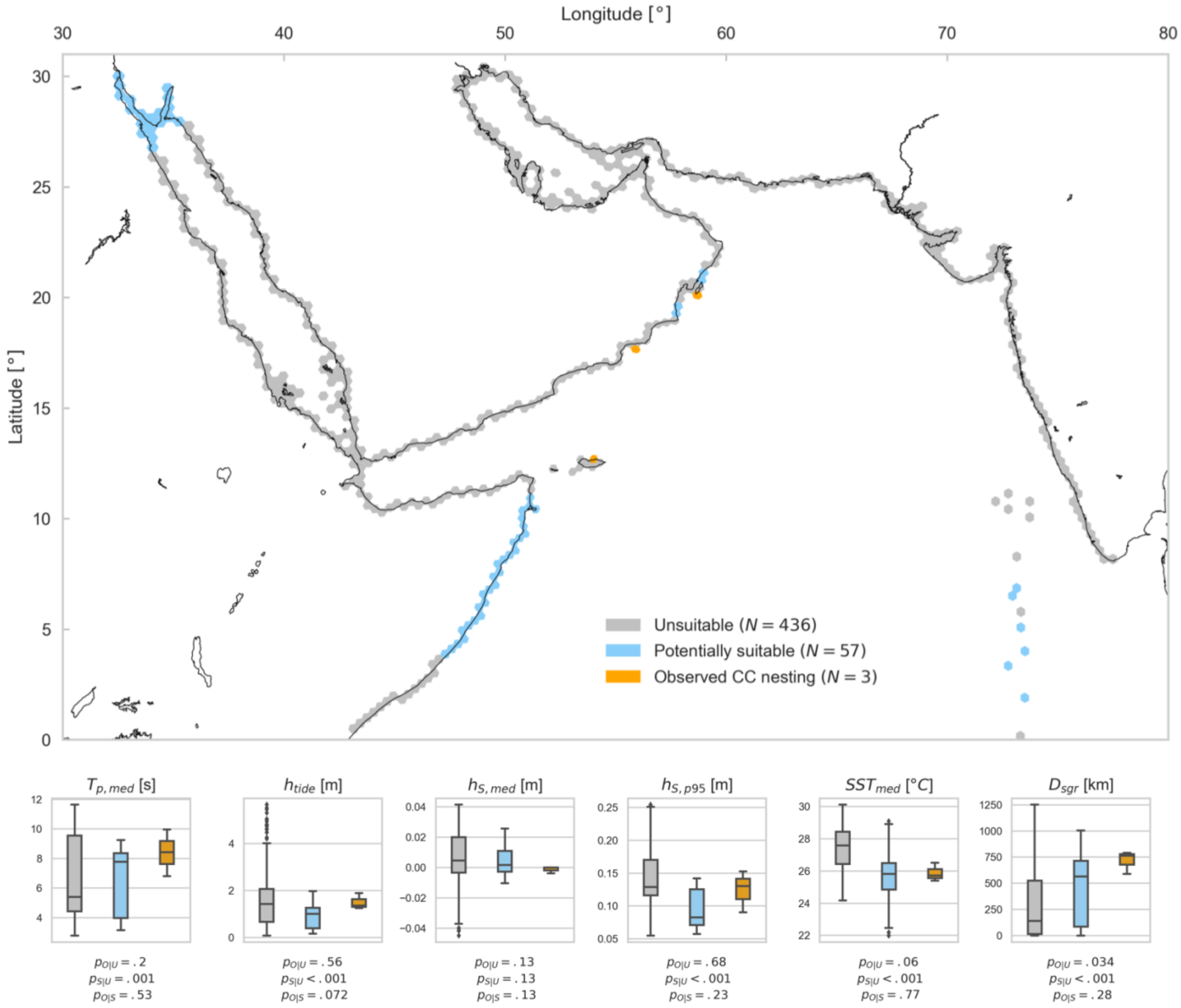


*Figure S33: Nesting suitability map for loggerhead turtles (CC) in the North West Indian*

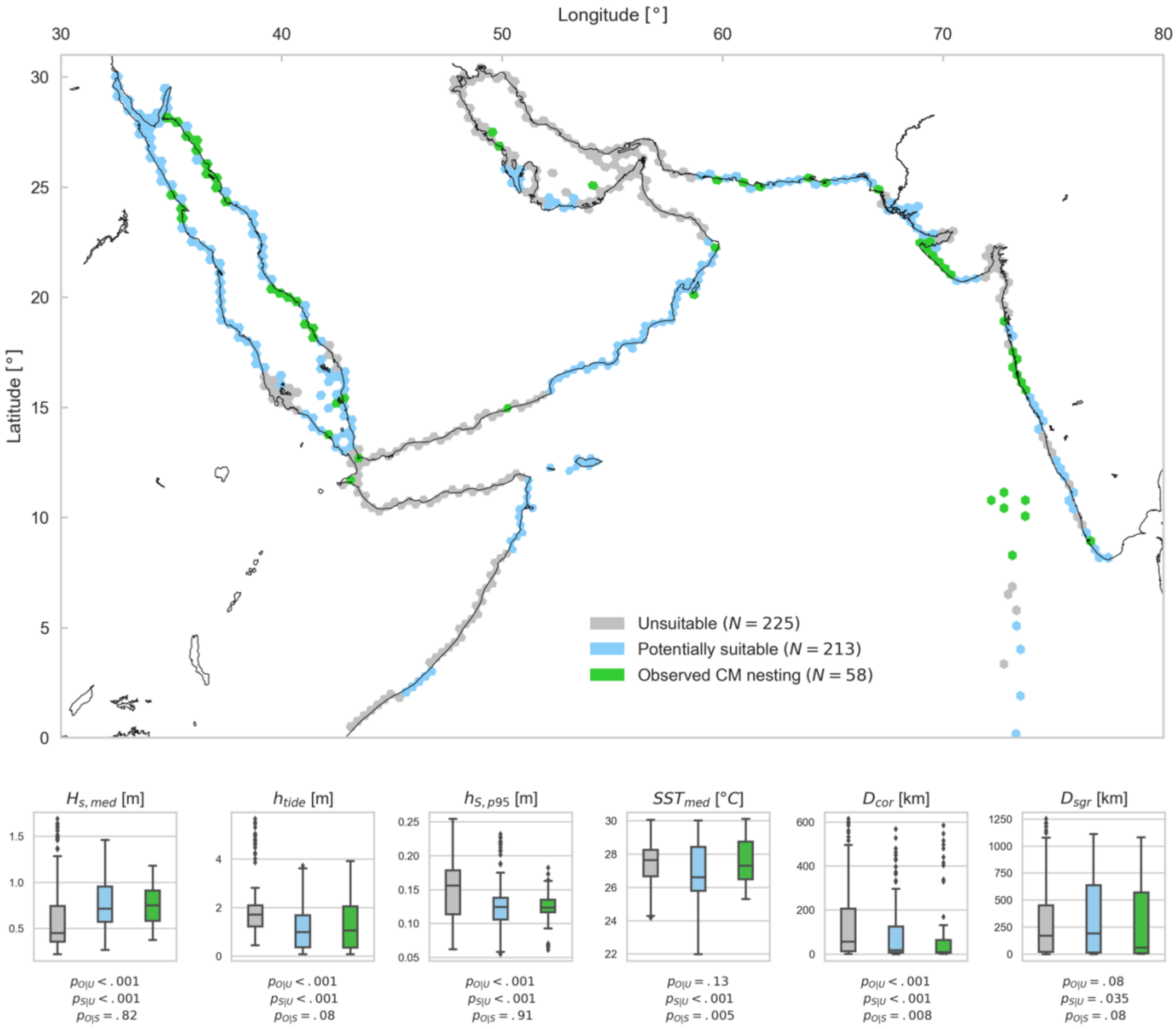


*Figure S34: Nesting suitability map for green turtles (CM) in the North West Indian*

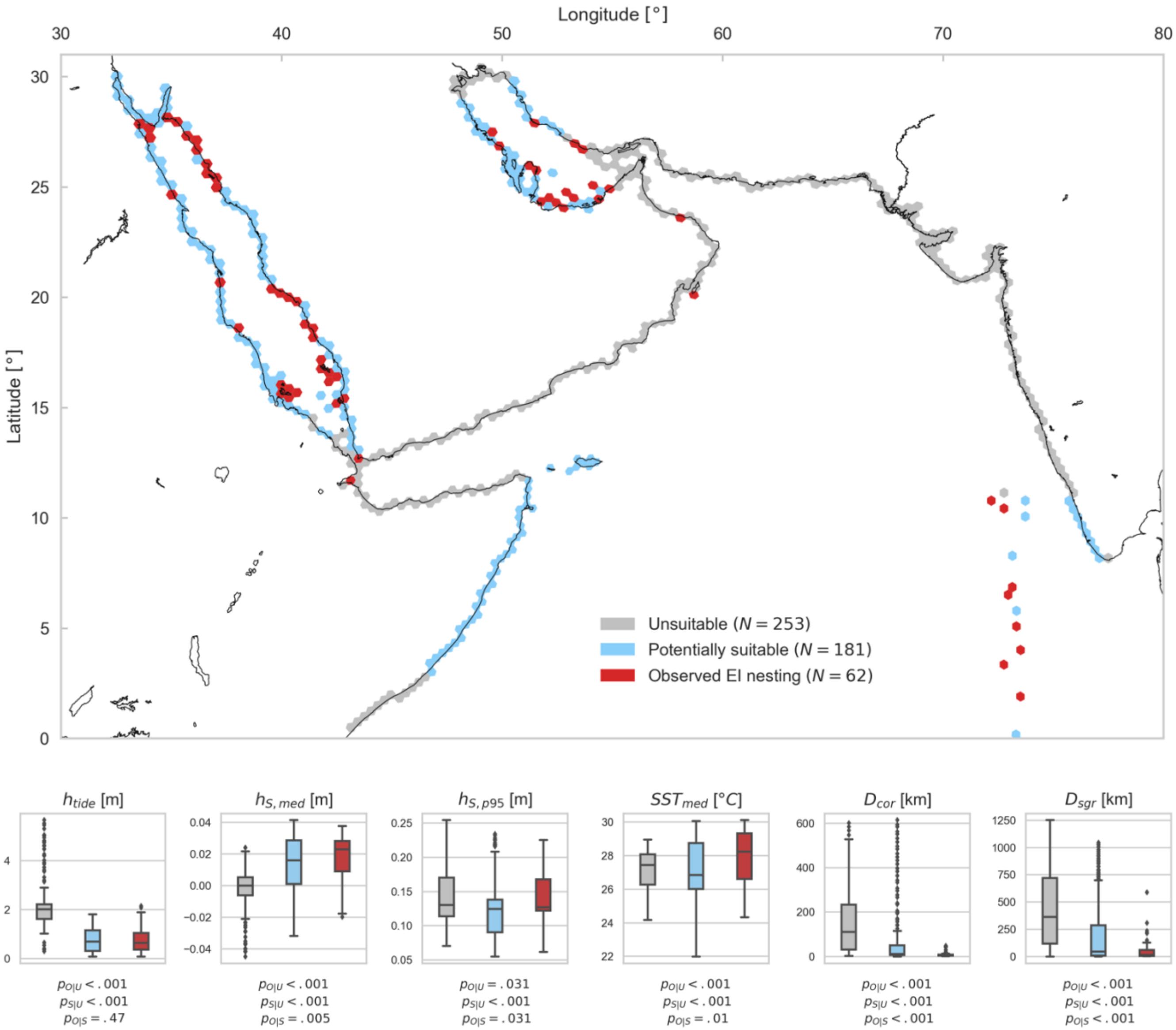


*Figure S35: Nesting suitability map for hawksbill turtles (EI) in the North West Indian*

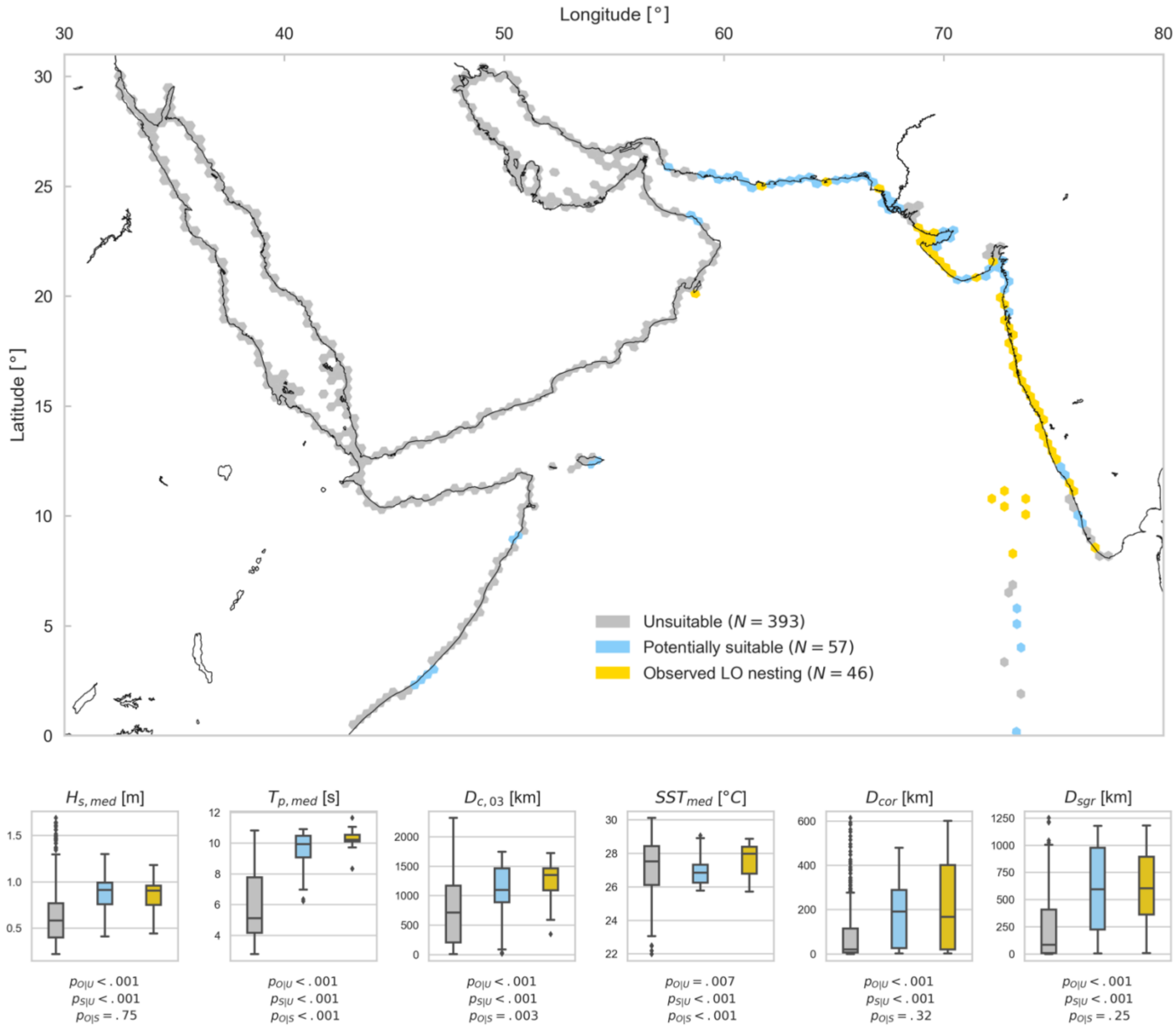


*Figure S36: Nesting suitability map for olive ridley turtles (LO) in the North West Indian*

S6.8 South West Indian (all species)

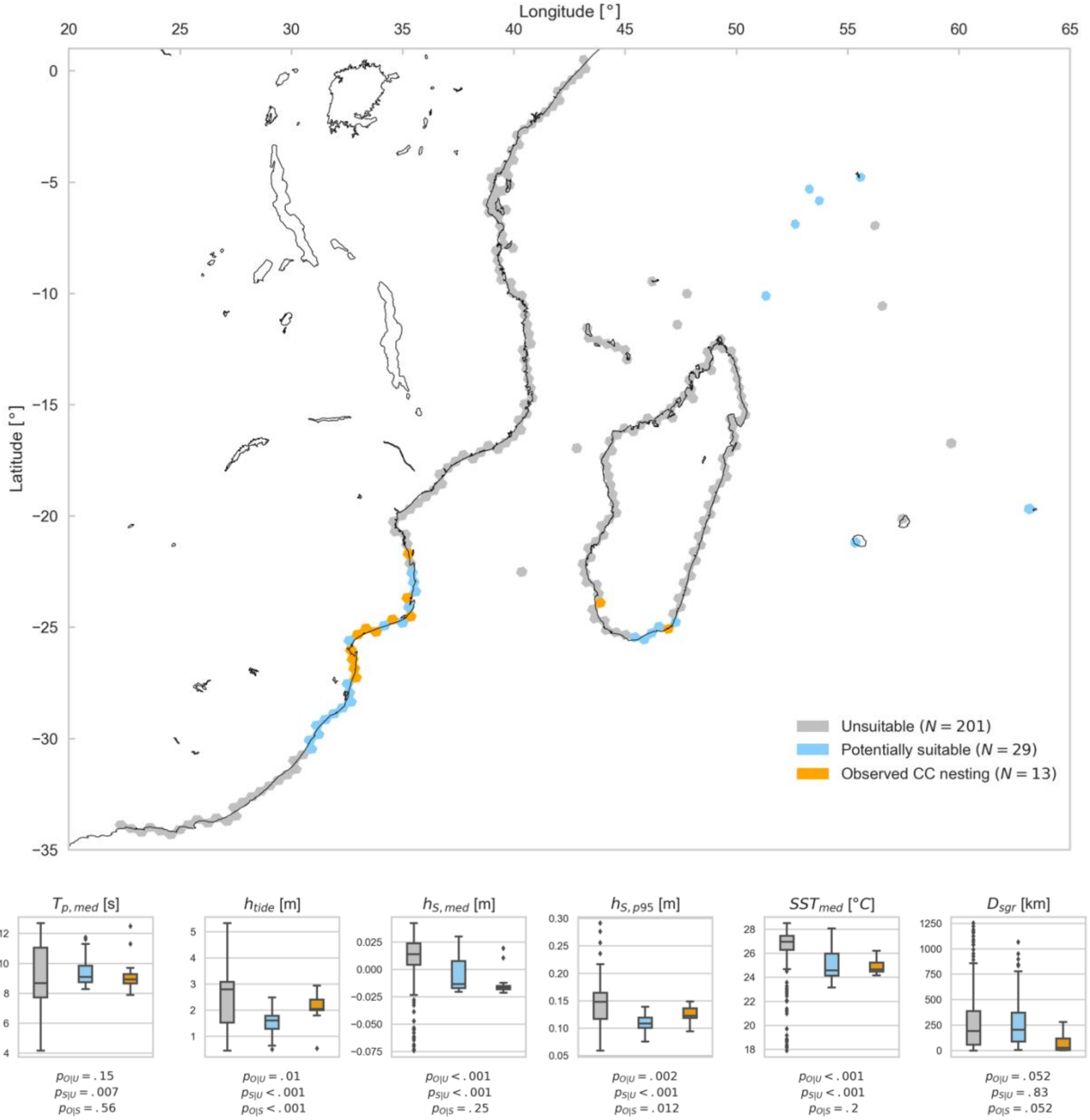


*Figure S37: Nesting suitability map for loggerhead turtles (CC) in the South West Indian*

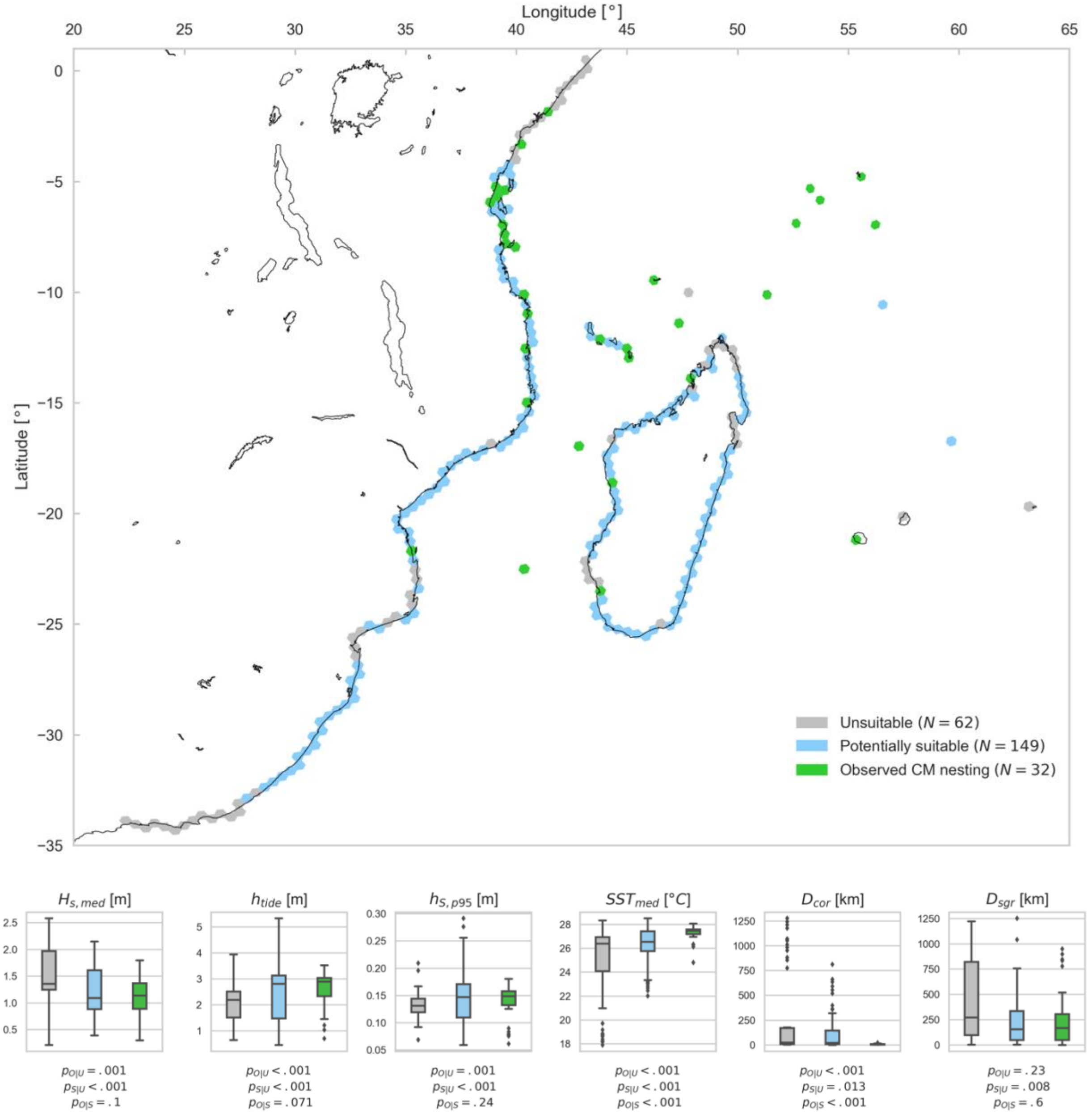


*Figure S38: Nesting suitability map for green turtles (CM) in the South West Indian*

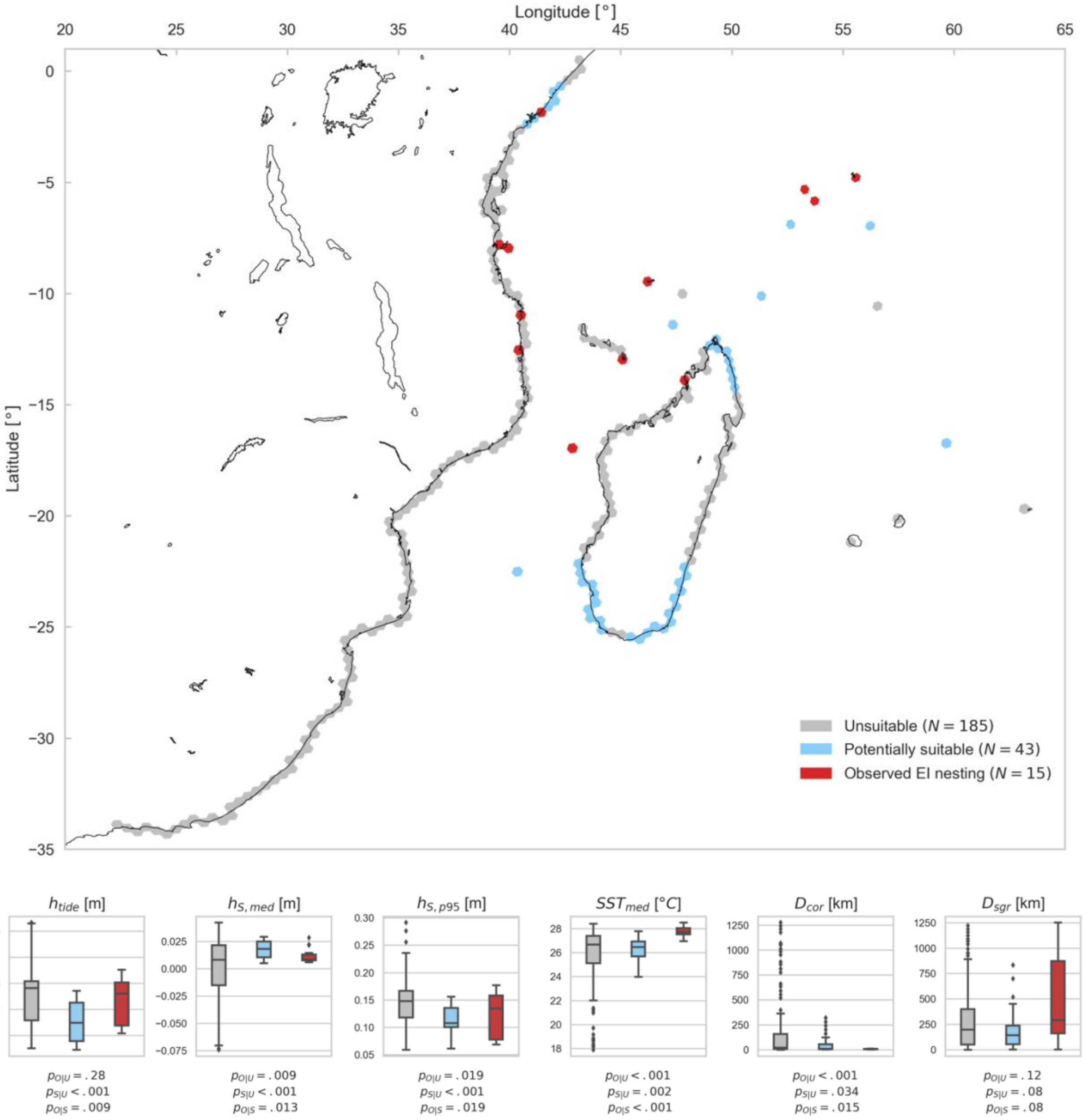


*Figure S39: Nesting suitability map for hawksbill turtles (EI) in the South West Indian*

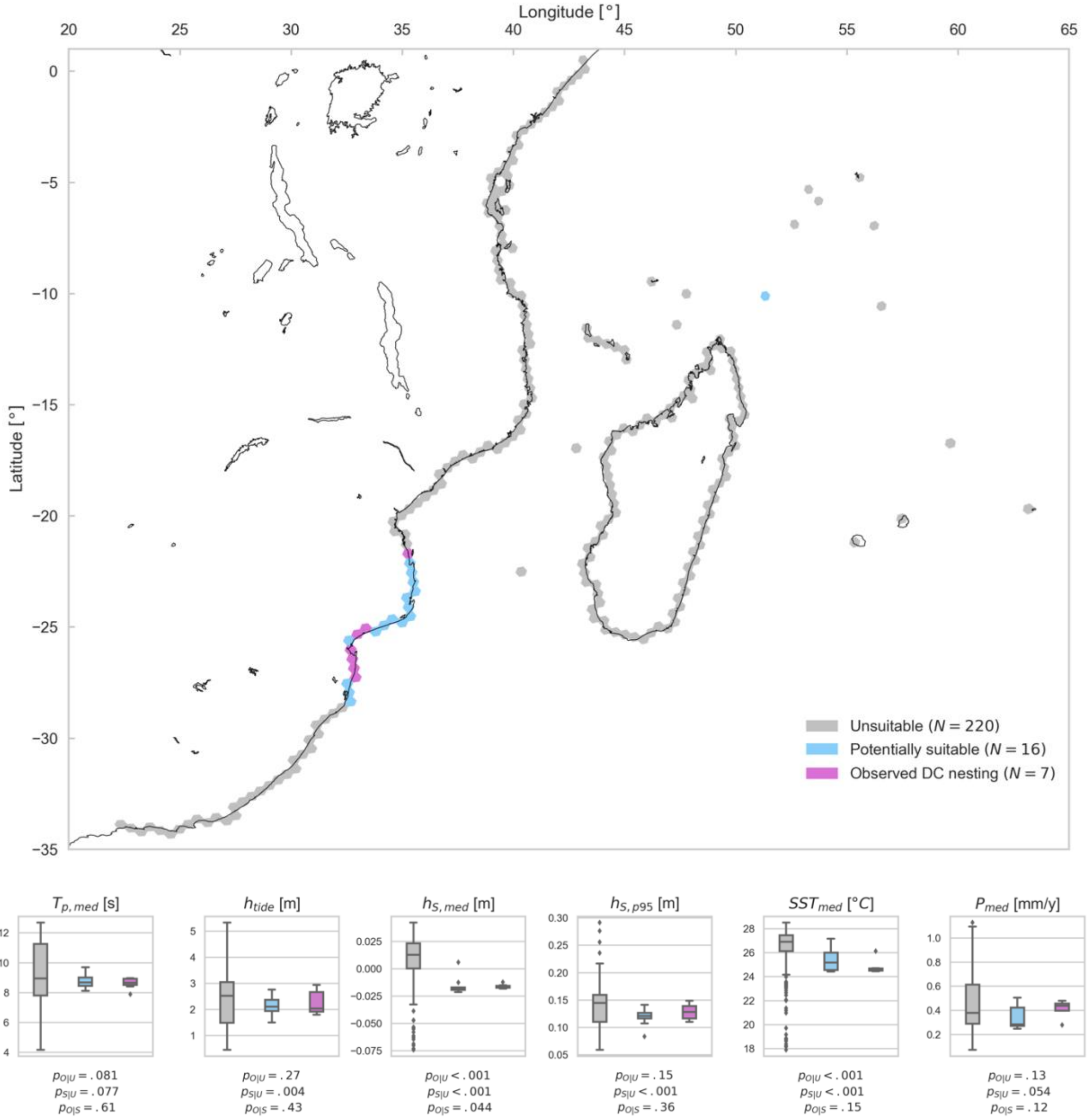


*Figure S40: Nesting suitability map for leatherback turtles (DC) in the South West Indian*

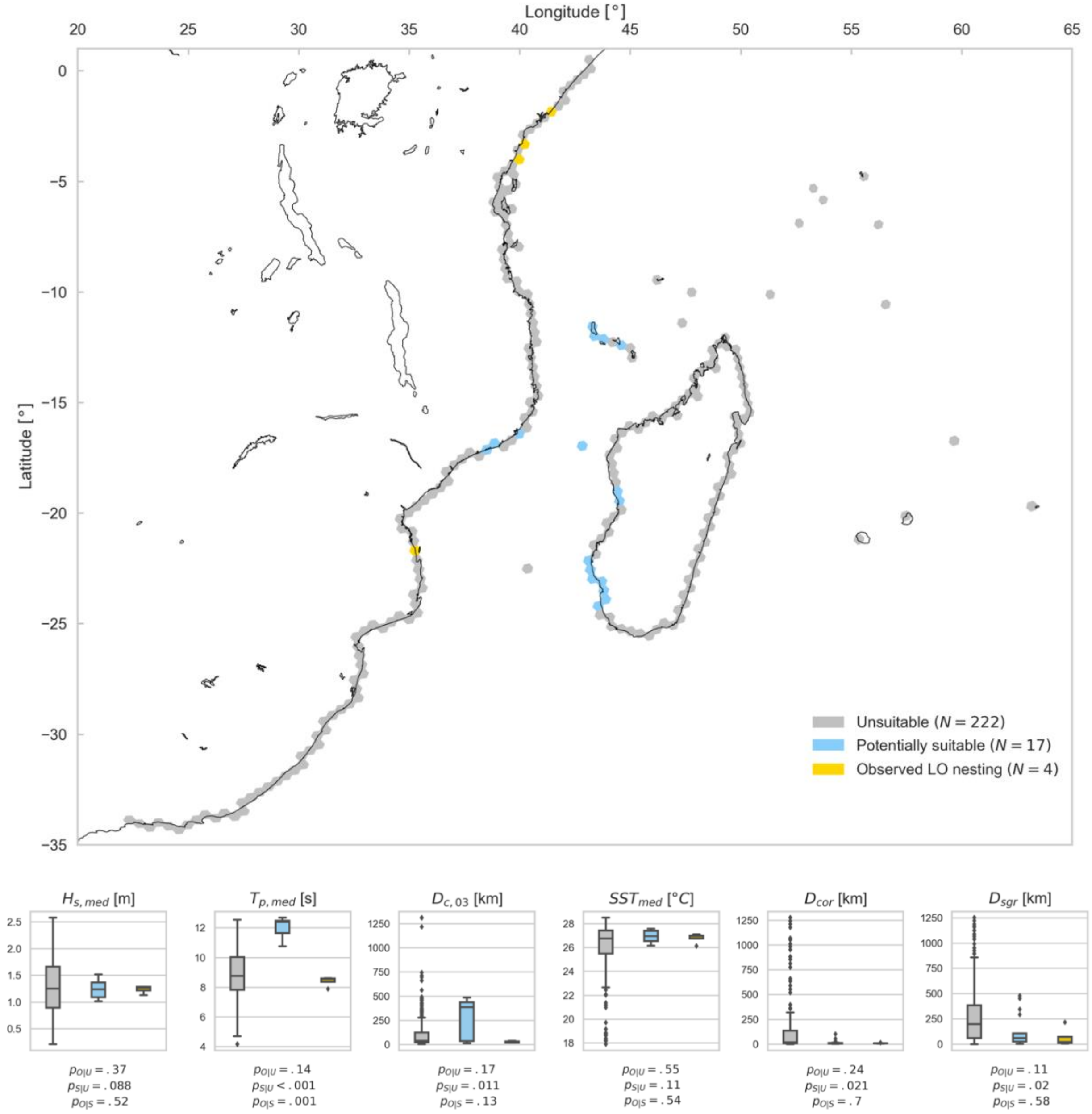


*Figure S41: Nesting suitability map for olive ridley turtles (LO) in the South West Indian*

S6.9 North East Indian (all species)

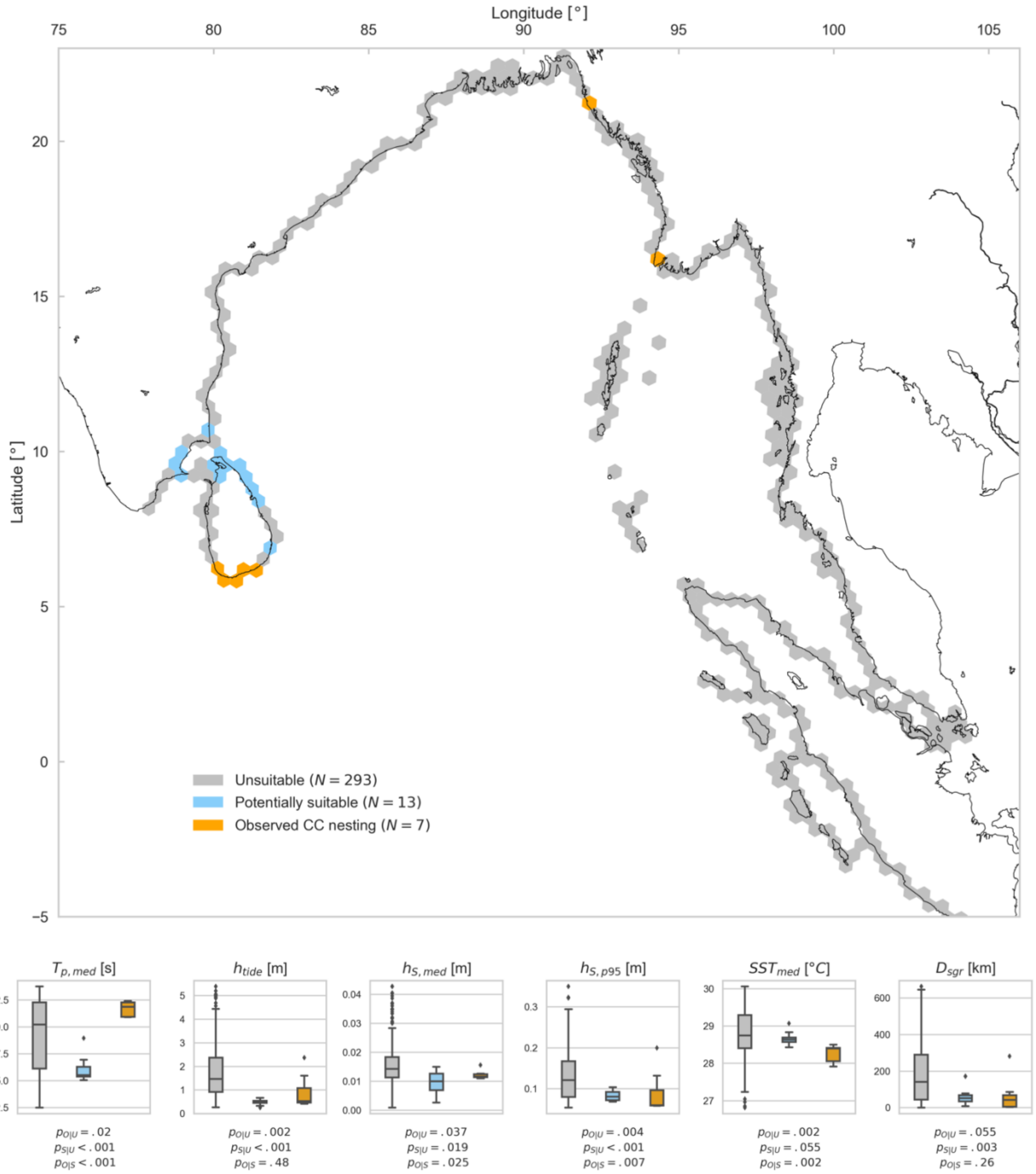


*Figure S42: Nesting suitability map for loggerhead turtles (CC) in the North East Indian*

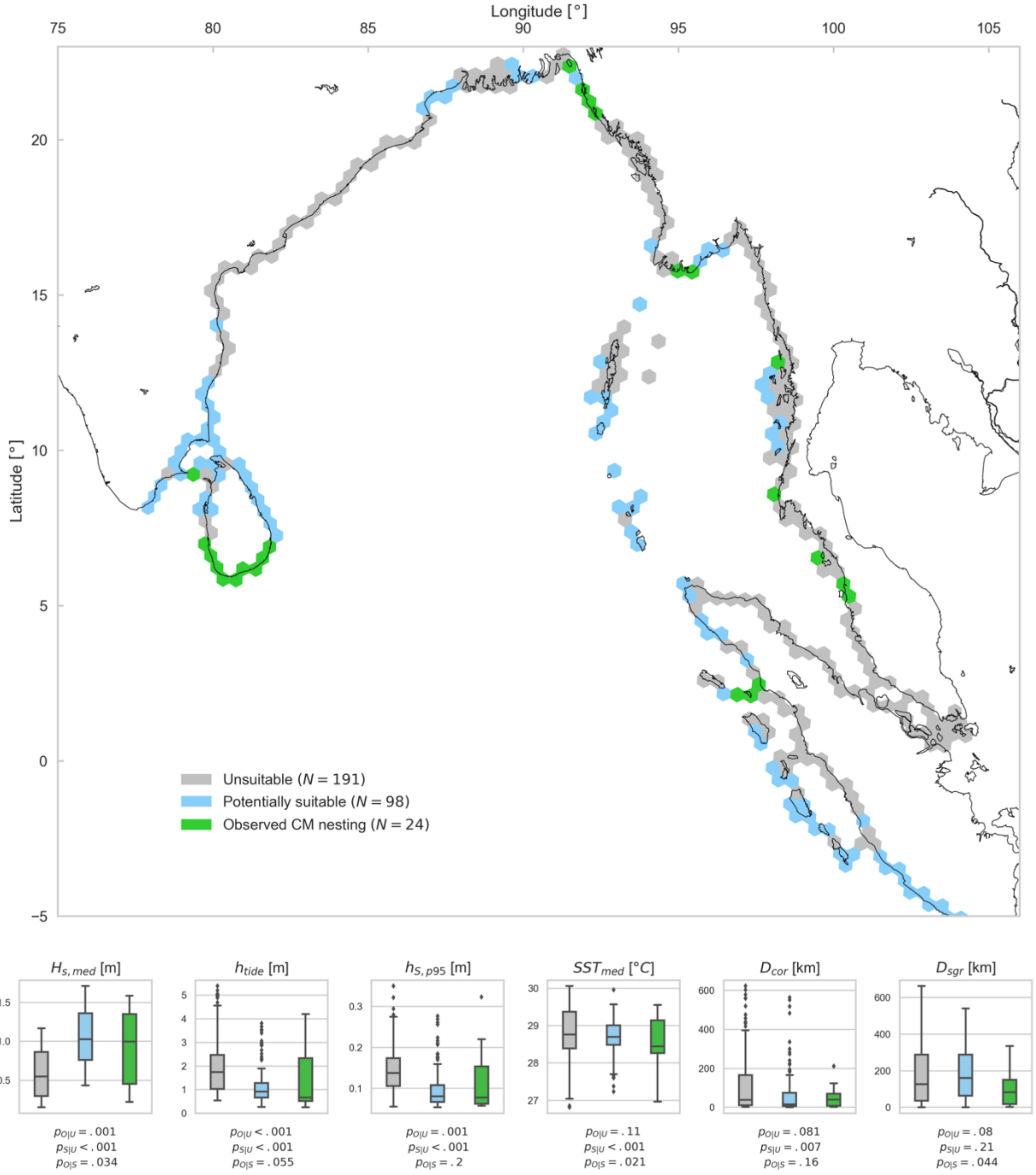


*Figure S43: Nesting suitability map for green turtles (CM) in the North East Indian*

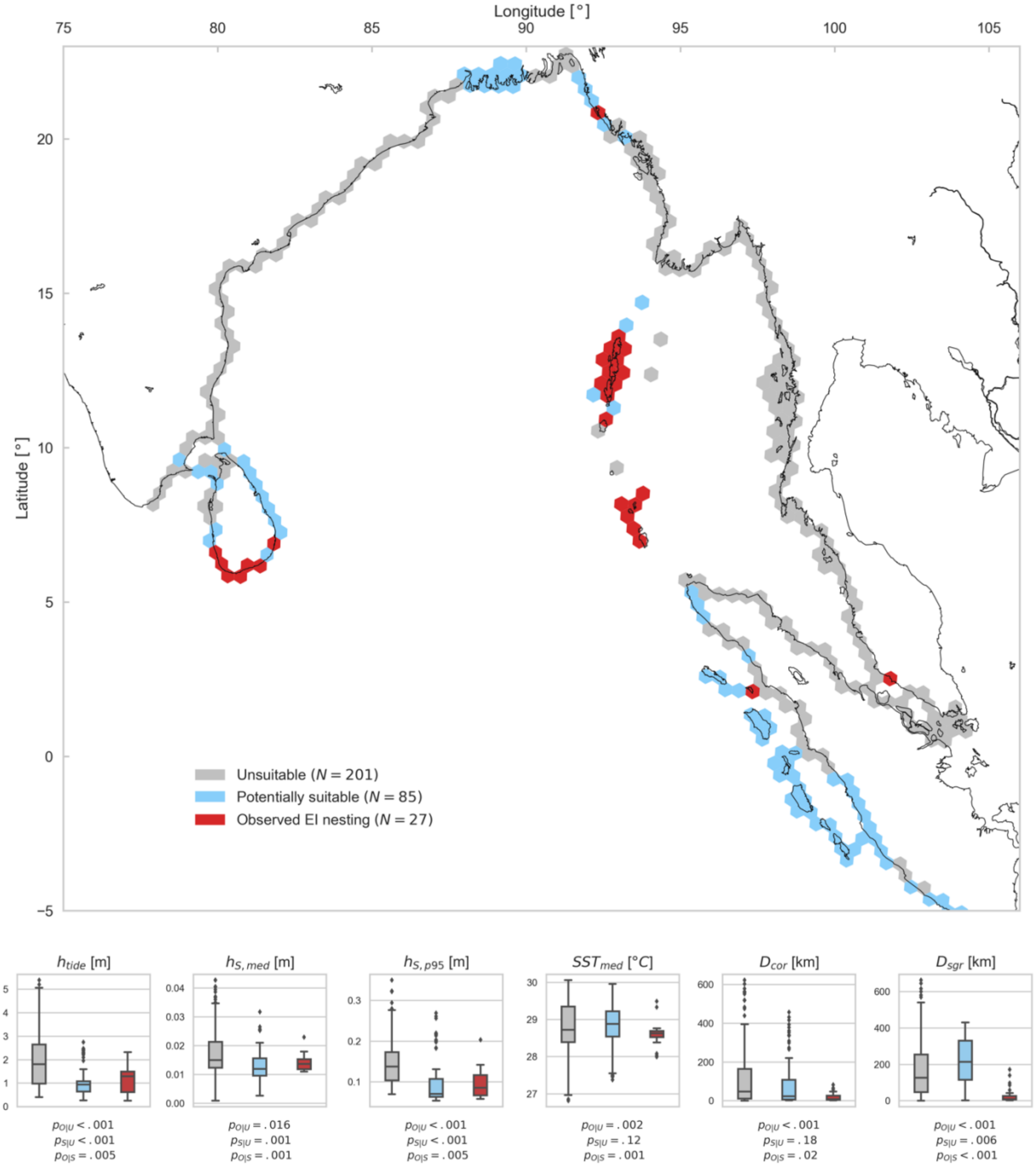


*Figure S44: Nesting suitability map for hawksbill turtles (EI) in the North East Indian*

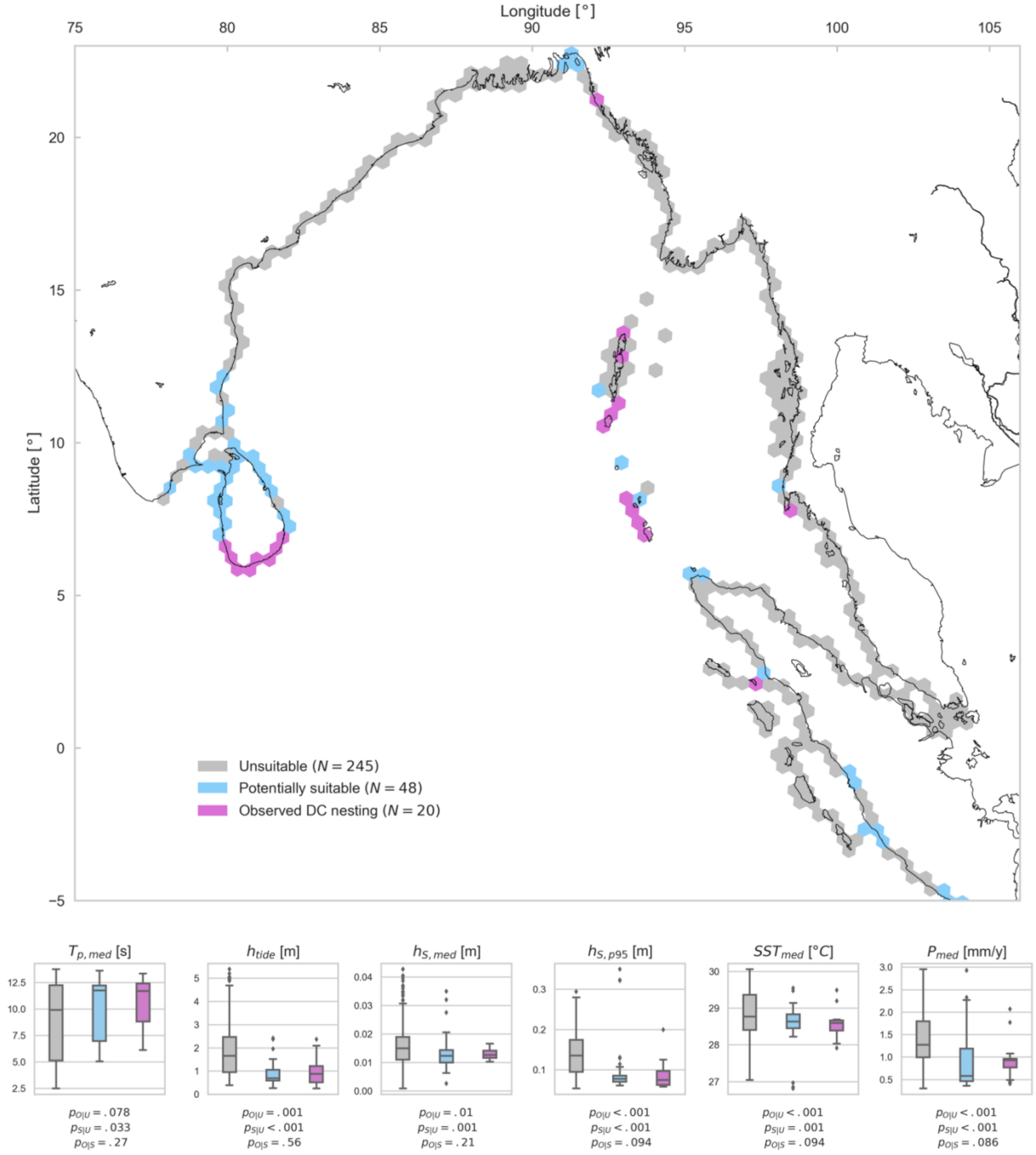


*Figure S45: Nesting suitability map for leatherback turtles (DC) in the North East Indian*

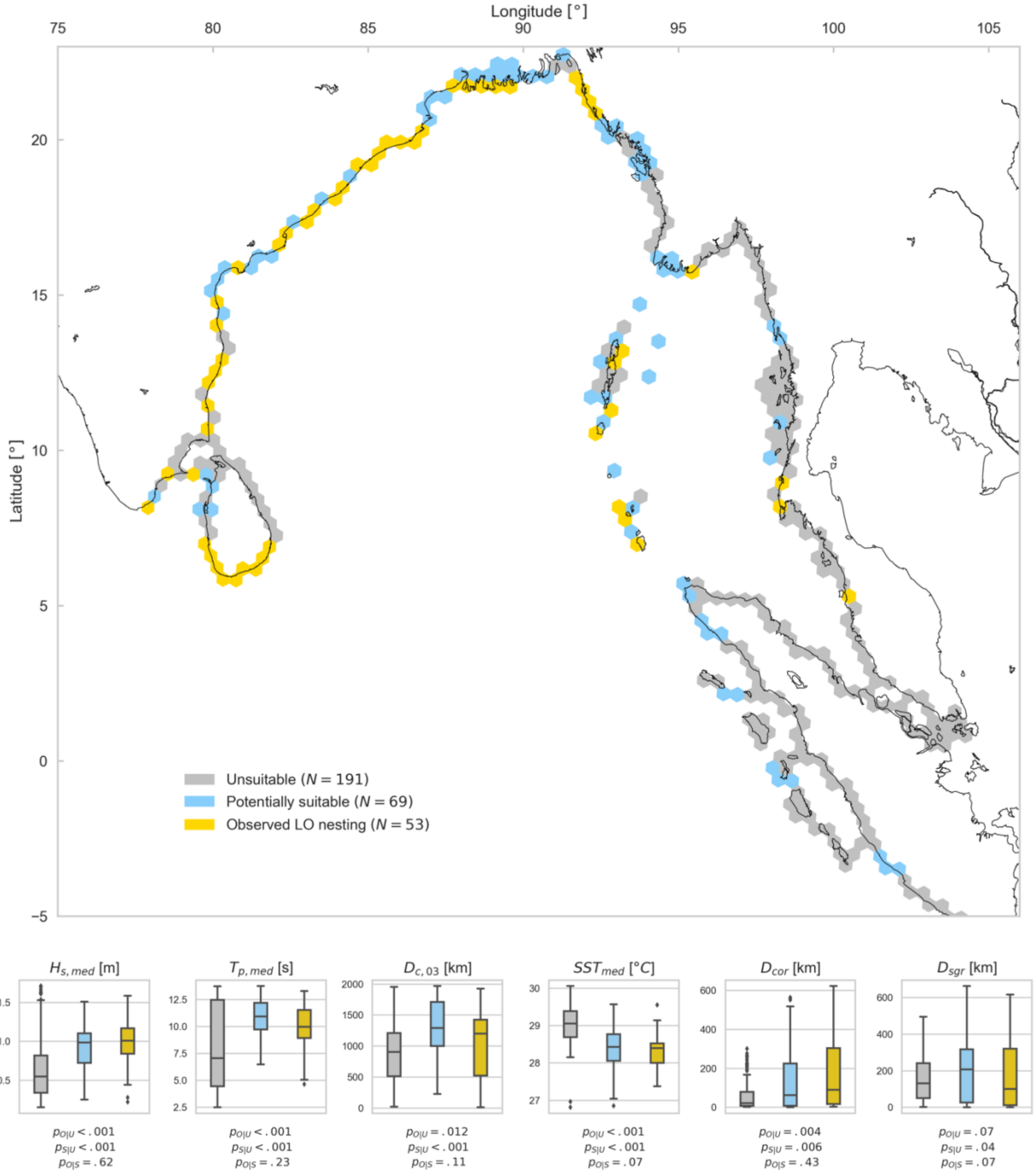


*Figure S46: Nesting suitability map for olive ridley turtles (LO) in the North East Indian*

## References


1. Hersbach, H. *et al.* ERA5 hourly data on single levels from 1979 to present. *Copernicus Climate Change Service (C3S) Climate Data Store (CDS)* https://cds.climate.copernicus.eu/cdsapp#!/dataset/reanalysis-era5-single-levels?tab=overview (2018) doi:10.24381/cds.adbb2d47.
2. Muis, S. *et al.* A High-Resolution Global Dataset of Extreme Sea Levels, Tides, and Storm Surges, Including Future Projections. *Front Mar Sci* **7**, 512955 (2020).
3. Copernicus Climate Change Service Climate Data Store. ORAS5 global ocean reanalysis monthly data from 1958 to present. *Copernicus Climate Change Service (C3S) Climate Data Store (CDS)* Preprint at https://doi.org/10.24381/cds.67e8eeb7 (2021).
4. Athanasiou, P. *et al.* Global Coastal Characteristics (GCC): A global dataset of geophysical, hydrodynamic and socioeconomic coastal indicators (submitted for publication). *Earth Syst Sci Data*.
5. Li, J. *et al.* A global coral reef probability map generated using convolutional neural networks. *Coral Reefs* **39**, 1805–1815 (2020).
6. UNEP-WCMC & Short, F. T. Global distribution of seagrasses (version 7.1). Seventh update to the data layer used in Green and Short (2003). *Cambridge (UK): UN Environment Programme World Conservation Monitoring Centre* Preprint at https://doi.org/10.34892/x6r3-d211 (2021).
7. Halpin, P. N. *et al.* OBIS-SEAMAP: The world data center for marine mammal, sea bird, and sea turtle distributions. *Oceanography* **22**, 104–115 (2009).
8. Kot, C. Y. *et al.* The State of the World's Sea Turtles Online Database: Data provided by the SWOT Team and hosted on OBIS-SEAMAP. *Oceanic Society, Conservation International, IUCN Marine Turtle Specialist Group (MTSG), and Marine Geospatial Ecology Lab, Duke University* http://seamap.env.duke.edu/swot (2021).
9. Christiaanse, J. C. & Antolínez, J. A. A. Coastgons: regional coastal characteristics along a global hexagonal coastline grid. https://doi.org/10.4121/68377ee4-892d-40f0-a490-29f2601e6825 (2023).
10. Wallace, B. P. *et al.* Regional Management Units for Marine Turtles: A Novel Framework for Prioritizing Conservation and Research across Multiple Scales. *PLoS One* **5**, e15465 (2010).
